# EBP1 nuclear accumulation negatively feeds back on FERONIA-mediated RALF1 signaling

**DOI:** 10.1371/journal.pbio.2006340

**Published:** 2018-10-19

**Authors:** Chiyu Li, Xuanming Liu, Xiaonan Qiang, Xiaoyan Li, Xiushan Li, Sirui Zhu, Long Wang, Yuan Wang, Hongdong Liao, Sheng Luan, Feng Yu

**Affiliations:** 1 State Key Laboratory of Chemo/Biosensing and Chemometrics, College of Biology, Hunan Province Key Laboratory of Plant Functional Genomics and Developmental Regulation, Hunan University, Changsha, People’s Republic of China; 2 Department of Plant and Microbial Biology, University of California, Berkeley, California, United States of America; 3 State Key Laboratory of Hybrid Rice, Hunan Hybrid Rice Research Center, Changsha, China; The Sainsbury Laboratory, United Kingdom of Great Britain and Northern Ireland

## Abstract

FERONIA (FER), a plasma membrane receptor-like kinase, is a central regulator of cell growth that integrates environmental and endogenous signals. A peptide ligand rapid alkalinization factor 1 (RALF1) binds to FER and triggers a series of downstream events, including inhibition of *Arabidopsis* H^+^-ATPase 2 activity at the cell surface and regulation of gene expression in the nucleus. We report here that, upon RALF1 binding, FER first promotes *ErbB3-binding protein 1* (*EBP1*) mRNA translation and then interacts with and phosphorylates the EBP1 protein, leading to EBP1 accumulation in the nucleus. There, EBP1 associates with the promoters of previously identified RALF1-regulated genes, such as *CML38*, and regulates gene transcription in response to RALF1 signaling. EBP1 appears to inhibit the RALF1 peptide response, thus forming a transcription–translation feedback loop (TTFL) similar to that found in circadian rhythm control. The plant RALF1-FER-EBP1 axis is reminiscent of animal epidermal growth factor receptor (EGFR) signaling, in which EGF peptide induces EGFR to interact with and phosphorylate EBP1, promoting EBP1 nuclear accumulation to control cell growth. Thus, we suggest that in response to peptide signals, plant FER and animal EGFR use the conserved key regulator EBP1 to control cell growth in the nucleus.

## Author summary

Receptor-like kinase FERONIA (FER) is an important regulator of plant growth and stress response and is activated by binding its peptide ligand, rapid alkalinization factor 1 (RALF1). However, how FER, a plasma membrane–localized receptor protein, regulates gene expression in the nucleus remains unclear. Here, we show that RALF1-FER signaling increases the abundance of ErbB3-binding protein 1 (EBP1) protein, which then accumulates in the nucleus and controls gene expression. The receptor kinase FER also directly interacts with and phosphorylates EBP1, a required step for EBP1 accumulation in the nucleus. Ultimately, EBP1 protein binds to the promoters of some RALF1-FER-regulated genes and inhibits their expression, leading to a negative regulation of RALF1-FER response. This study thus reveals a link between a plasma membrane receptor and the control of gene expression in the nucleus and establishes a similar mode of action for EBP1 in both animals and plants.

## Introduction

FERONIA (FER) is a versatile receptor-like kinase (RLK) that controls nearly all aspects of plant cellular activity [1,2]. FER was originally reported to regulate pollen tube reception for successful double fertilization [3,4]. Further studies have revealed its multiple roles in regulating vegetative cell growth. For instance, FER is required for root hair elongation in response to auxin [5]. FER is also essential for the expansion of leaf cells associated with brassinolide (BR) response [6] and for hypocotyl cell elongation in relation to ethylene biosynthesis [7] and signal transduction [8]. FER also regulates fruit ripening in strawberry [9] and tomato [10] via ethylene biosynthesis regulation [7]. Our recent work has shown that two *FER-like receptor* (*FLR*) genes in rice are crucial for cell growth [11]. In addition, FER is involved in biotic and abiotic stress responses. *fer* mutants are hypersensitive to salt [12,13,14], cold, and heat stress [12] and are hypersensitive to nickel (Ni) ions but are tolerant to the heavy metals cadmium (Cd), copper (Cu), lead (Pb), and zinc (Zn) [15]. The roles of FER in stress response might be particularly attributable to its function in regulating abscisic acid (ABA) and rapid alkalinization factors (RALFs) signal transduction [12,16,17]. Studies have shown that *fer* mutants show a hypersensitive response to exogenous ABA with respect to stomatal closure and primary root growth [17]. FER suppresses ABA signaling by activating an A-type protein phosphatase 2C (PP2C), ABA insensitive 2 (ABI2), through the guanine nucleotide exchange factor–plant RHO-related GTPases (GEF-ROP/RAC) pathway, which in turn inhibits the ABA response mediated by sucrose nonfermenting 1–related protein kinase 2 (SnRK2) [17]. The activated ABI2 phosphatase interacts with and dephosphorylates FER to reduce FER activity, providing a cross-talk node between ABA and the RALF1 (a ligand of FER) peptide [12]. RALF1 is a 5-kDa peptide that rapidly induces alkalinization of cell culture medium and inhibits cell growth [18,19]. RALF1 binds to FER, increases FER phosphorylation, and further inhibits *Arabidopsis* H^+^-ATPase 2 (AHA2) activity, leading to inhibition of root cell elongation [20]. Previous studies have shown that mutations in FER can alter fungal invasion [21,22], implicating FER in immune responses. Along this line, other RALF-family peptides—including RALF17, RALF23, and RALF33—may work with FER to regulate 22 amino acid fragment of bacterial flagellin (flg22)-triggered reactive oxygen species (ROS) burst [16]. It is further shown that FER exerts its control on immune signaling through a scaffold function to facilitate the complex formation of the immune receptor complexes kinases EF-Tu receptor (EFR) and flagellin-sensing 2 (FLS2) with their coreceptor brassinosteroid insensitive 1–associated kinase 1 (BAK1) to initiate immune signaling [16]. FER also works with several other proteins, such as RPM1-induced protein kinase (RIPK) [23] and LLG1 [24], to transmit RALF1 signal.

Although a handful of components have been identified in the RALF1 peptide signaling pathway, the mechanism by which FER regulates nuclear events remains unknown. A common theme of signal transduction from the cell surface to the nucleus involves the modification of cytoplasmic proteins by membrane receptors and then their accumulation in the nucleus to alter gene expression [25,26]. In animals, this scheme has been well demonstrated by studies of the epidermal growth factor receptor (EGFR) family [27]. EGFRs consist of four distinct receptors: erythroblastic leukemia viral oncogene homolog 1 (ErbB1), ErbB2, ErbB3, and ErbB4 [28]. ErbB3 is the receptor of heregulin (HRG)/neuregulin (NRG) peptides [29,30]. When the HRG ligand peptide binds the ErbB3 receptor in breast cancer cells, ErbB3-binding protein 1 (EBP1) is phosphorylated by ErbB3 and accumulates in the nucleus [31]. Thus, in the breast cancer cell, EBP1 functions as a negative regulator of HRG-ErbB3 signal transduction [31], and overexpression of EBP1 results in reduced cell growth and increased differentiation [32]. EBP1 is a DNA- [33] and RNA-binding [34,35] protein, and the proper localization of EBP1 is critical for its growth-suppressive properties [34]. In HeLa cell nuclei, EBP1 interacts with the E2F1 complex to suppress E2F1-regulated gene transcription [33]. Meanwhile, EBP1 also interacts with other transcriptional repressors—such as retinoblastoma gene (Rb) [36], Sin3A [37], and histone deacetylase 2 (HDAC2) [38]—to suppress downstream gene transcription. EBP1 is also an RNA-binding protein [34,35] as a part of ribonucleoprotein (RNP) complexes. EBP1 not only associates with mature and precursor rRNA species [34,35] but also directly binds to some mRNA species and regulates their translation [35,39].

EBP1 is evolutionarily conserved in both animals and plants. *Arabidopsis* EBP1 has been previously identified as a protein that controls cell size and was named *At*CPR [40]. For simplicity, hereafter, we will use the name EBP1 in this report. Overexpression of EBP1 homologs in *Solanum tuberosum* [41], *Arabidopsis* [41], *Zea mays* [42], and *Hevea brasiliensis* [43] regulate organ size in a dose-dependent manner [41], suggesting that the abundance of EBP1 is critical for its function in plant growth regulation. Plant EBP1 is also implicated in stress responses. For example, overexpression of *AcEBP1* (*Atriplex canescens*) increases plant low temperature, NaCl and ABA sensitivity [44], and drought stress resistance [43,44]. However, despite these overexpression analyses, it remains unknown how EBP1 is regulated by upstream signaling events. In search of FER partner proteins, we identified EBP1 as one of the interacting proteins. We found that RALF1-FER signaling enhanced *EBP1* mRNA translation and further promoted its phosphorylation and nuclear accumulation in plants. EBP1 can directly bind to some chromatin loci and regulate their expression, thus connecting RALF1-FER signaling with gene regulation in the nucleus.

## Results

### EBP1 physically associates with FER RLK

**Fig 1:**
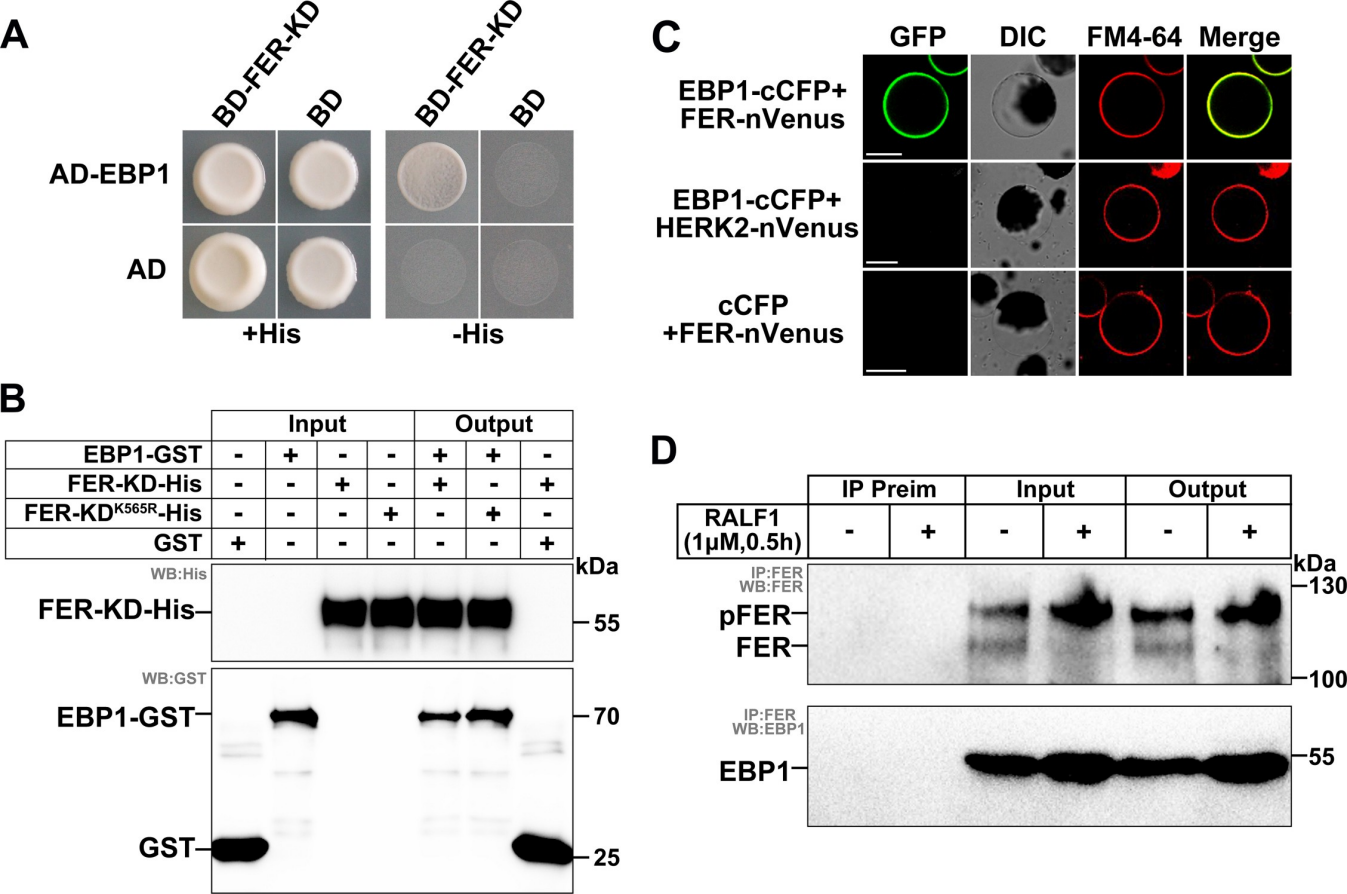
EBP1 physically interacts with FER. (A) Y2H assays show interaction between EBP1 and FER. SD/-Ade/-Leu/-His selection medium containing 20 mM 3-AT was used for screening yeast growth. (B) GST pull-down assays. The indicated GST-tag and His-tag were detected by anti-GST and anti-His, respectively. (C) EBP1 interacts with FER in BiFC assay in *Arabidopsis* protoplasts. Negative controls (EBP1-cCFP+HERK2-nVenus and cCFP+FER-nVenus) are shown. GFP, FM4-64, DIC, and merged images are shown, bar = 20 μm. (D) Co-IP assays. The immunoprecipitated FER and coimmunoprecipitated EBP1 were indicated using anti-FER and anti-EBP1 antibodies. pFER indicates the phosphorylated FER. The dephosphorylated form of FER is labeled as FER. Input lanes are indicated. Preimmune serum (“Preim”) was used as negative control. Seedlings were treated with or without 1 μM RALF1 for 30 minutes. All assays were performed on at least three biological replicates, and similar results were obtained. AD active domain; BD, binding domain; BiFC, bimolecular fluorescence complementation; cCFP, C-terminal cyan fluorescent protein; Co-IP, coimmunoprecipitation; DIC, differential interference contrast; EBP1, ErbB3-binding protein 1; FER, FERONIA; GFP, green fluorescent protein; GST, glutathione S-transferase; pFER, phosphorylated FER; RALF1, rapid alkalinization factor 1; WB, western blot; Y2H, yeast two-hybrid.

To identify other components in the RALF1-FER/RIPK signaling pathway, a yeast two-hybrid (Y2H) screen was performed using the FER kinase domain (FER-KD, 469–896 amino acids [aa]) as a bait against an *Arabidopsis* cDNA library [12,17,23]. One truncated version (229–401 aa) of EBP1 was identified as an interacting clone. To confirm this interaction, we cloned the full-length protein of EBP1 into an active domain (AD) vector and FER-KD into a binding domain (BD) vector. The Y2H assay showed that the full-length protein of EBP1 indeed interacted with the FER-KD in yeast cells (Fig 1A). We also found that EBP1 interacted with other FER relatives that belong to the *Catharanthus roseus* receptor-like kinase 1-like kinase (*Cr*RLK1L) family, such as ANXUR 1 (ANX1), AT5G24010, and CURVY 1 (CVY1), in the yeast system (S1 Fig). A glutathione S-transferase (GST) pull-down assay showed that the full-length EBP1 protein tagged with GST (S2A Fig) and FER-KD tagged with 6 × His [12] were copurified (Fig 1B). EBP1 also interacted with a kinase-dead version of FER-KD containing the Lys^565^-to-Arg mutation (FER-KD^K565R^-His) [4] in a GST pull-down assay (Fig 1B). We further confirmed the interaction between EBP1 and FER *in planta* by a bimolecular fluorescence complementation (BiFC) assay in *Arabidopsis* protoplasts. We simultaneously transferred EBP1–C-terminal cyan fluorescent protein (cCFP) and FER-nVenus (or HERCULES2 [HERK2]-nVenus as negative control) into *Arabidopsis* protoplasts to observe the reconstituted fluorescence. We found that EBP1 interacted with FER in the plasma membranes (indicated by the styryl dye FM4-64) of *Arabidopsis* protoplasts but did not interact with HERK2 (Fig 1C). We performed the western blot to show that the proteins in the BiFC assay were expressed (S2B Fig). We also performed a coimmunoprecipitation (Co-IP) assay to examine the interaction between EBP1 and FER *in planta* using polyclonal antibodies against FER [12,23] and EBP1 (S2C–S2E Fig). We used plant materials with or without RALF1 treatment and found that FER and EBP1 interaction was detected in both RALF1-treated and untreated samples (Fig 1D). Using *Ubi*::FER-FLAG transgenic plants [12], the interaction between EBP1 and FER was also confirmed via Co-IP assay (S3 Fig). Taken together, these data indicate that FER physically interacts with EBP1.

EBP1 controls cell growth and proliferation in human cancer cell lines [32,45] and some higher plants [41,42,43]. To investigate whether EBP1 is evolutionarily conserved, we assessed 823 EBP1-like protein sequences in Animalia, Plantae, Fungi, and Protista (S4 Fig). In *Arabidopsis*, we found only one EBP1 or EBP1-like protein (S4 Fig). Plant EBP1s showed high sequence similarity (S5 Fig). Using the UniProt Knowledgebase [46], we further noticed that 10 α-helixes, 12 β-strands, and one turn might exist in the EBP1 secondary structure (S5 Fig). An additional putative nucleus-localization sequence (NLS) and two regions that resemble nuclear localization signals are conserved in *Arabidopsis* EBP1 (S5 Fig).

### *EBP1* mRNA is expressed in root

To investigate the tissues and organs in which *EBP1* mRNA may be expressed, we constructed *proEBP1*::*GUS* transgenic *Arabidopsis* and examined the pattern of β-glucuronidase (GUS) activity at different growth stages (S6A–S6J Fig). In seedlings that were 7 days after germination (DAG), *proEBP1*::GUS was expressed mainly in cotyledons and roots (S6B, S6F and S6H Fig). GUS activity was also detected at the radicle tip (S6D Fig). In 4-week-old *proEBP1*::GUS rosettes, the GUS activity was lower than that in 7-DAG seedlings, and slight GUS activity was detected in the vascular tissues and the mesophyll cells (S6J Fig). No GUS activity was detected in nontransgenic plants (S6A, S6C, S6E, S6G and S6I Fig). We further analyzed the *EBP1* mRNA expression pattern using ePlant [47] and obtained similar expression patterns to those in the GUS staining assays (S6K Fig). These data showed that *EBP1* was expressed in root and cotyledon at the early stage of plant growth. However, the EBP1 protein level and localization in the next experiment showed that *EBP1* mRNA translation is tightly regulated.

### RALF1-FER enhances *EBP1* mRNA translation, leading to increased EBP1 protein level

**Fig 2:**
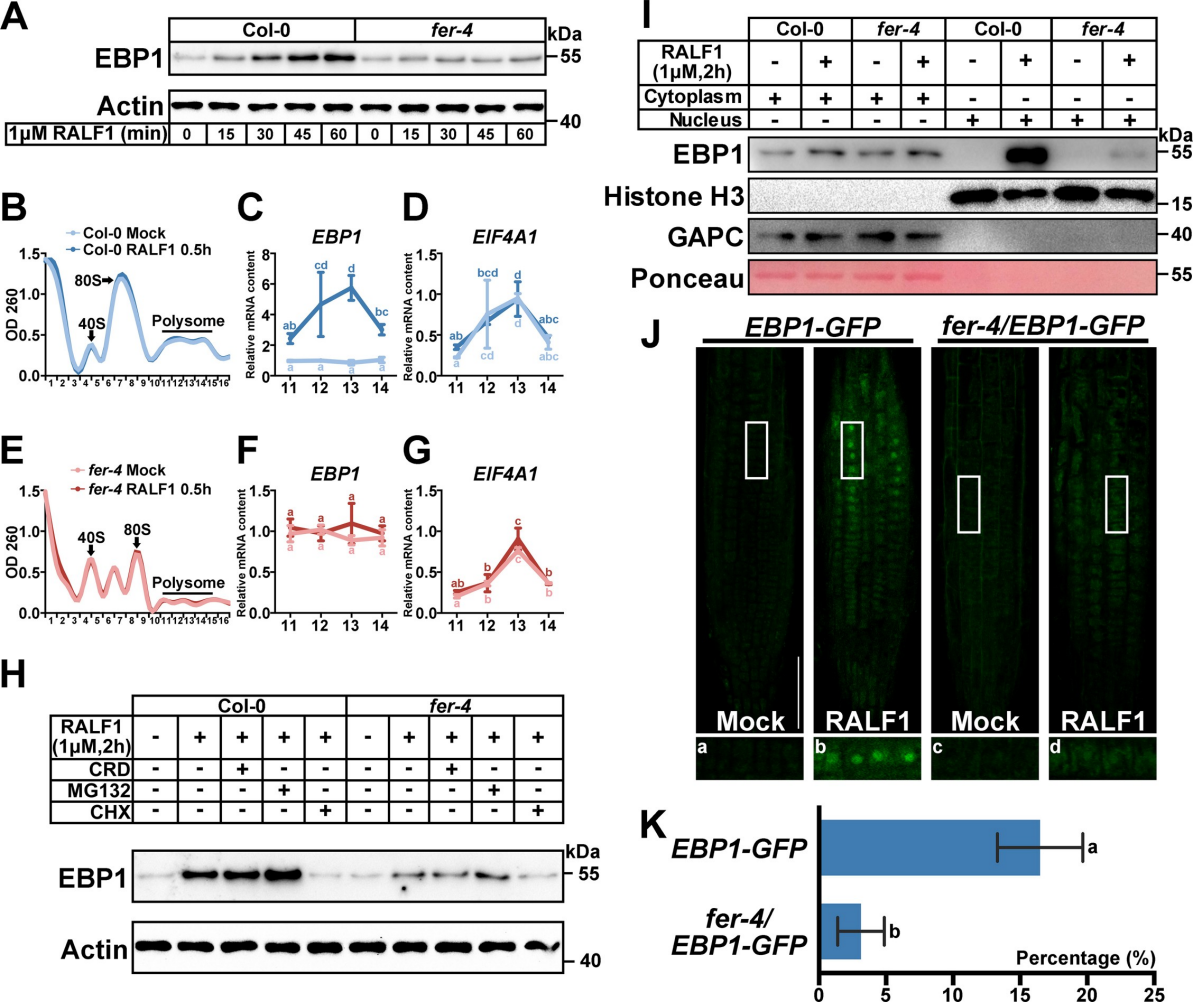
RALF1-induced translation and nuclear accumulation of EBP1 are partially FER-dependent. (A) Time course of EBP1 accumulation in Col-0 and *fer-4* plants after 1 μM RALF1 treatment. Actin was used as loading control. (B-G) Polysome profiling assay with sucrose density gradient accompanied by qRT-PCR to analyze the translational status of *EBP1* and *EIF4A1* mRNA. The OD260 absorption was monitored together with fractionation. The fractions containing 40S, 80S of ribosome, and polysomes in Col-0 (B) and *fer-4* (E) (with or without 1 μM RALF1 treatment for 30 minutes, respectively) are indicated. The abundance mRNA in the fractions (11–14) of *EBP1* (C, F) and *EIF4A1* (D, G) were detected by qRT-PCR. *Actin* was used as a reference gene. Three biological replicates were performed, and similar results were obtained. (H) RALF1 (1 μM for 2 hours) accelerates *EBP1* mRNA translation, which was abolished by CHX. Actin was indicated as a loading control. (I) Immunoblot analyses of EBP1 in both nuclear and nuclei-depleted soluble fractions from RALF1 treated (1 μM for 2 hours) or untreated Col-0 and *fer-4*, respectively. Antibody against Histone H3 was used to mark the nucleus fraction. Antibody against GAPC and Ponceau S staining was used to mark cytosolic fraction. (J) RALF1-induced nucleus accumulation of EBP1-GFP in root cells of 7-DAG seedlings harboring *35S*::*EBP1-GFP* (*EBP1-GFP* for short) and *fer-4*/*EBP1-GFP*, respectively. Seedlings were treated with 0 μM or 1 μM RALF1 peptide for 2 hours. Close-up of the pictures are selected (with white frames) and displayed in a–d, respectively. All the fluorescence images were taken using the same parameter of laser scanning confocal microscope. Three independent lines of *35S*::*EBP1-GFP* were analyzed, and similar results were obtained. Bar = 50 μm. (K) Percentages of cells with GFP signals in the nucleus. At least 2,300 cells were quantified from 19 independent roots for each genotype per experiments. Three biological replicates were done independently, and similar results were obtained. At least five biological replicates of panels A, H, and I were performed, and similar results were obtained. Data represent means. Data points are means +/− SD. Values with different letters are significantly different (*P* < 0.05) from each other, tested by one-way ANOVA. Numerical data used to generate the plot in B-G and K are provided in S1 Data. CHX, cycloheximide; CRD, cordycepin; DAG, day after germination; EBP1, ErbB3-binding protein 1; FER, FERONIA; GAPC, cytosolic glycolytic GAPDHs; GFP, green fluorescent protein; qRT-PCR, quantitative reverse transcription PCR; RALF1, rapid alkalinization factor 1.

In the Co-IP assays (Fig 1D), we noticed that RALF1 may enhance the accumulation of EBP1 protein. We investigated this possibility using western blotting. Both Col-0 and the *fer-4* mutant were treated with RALF1, and total protein was collected at the indicated time points (Fig 2A). Although the protein content of EBP1 increased in both Col-0 and the *fer-4* mutant after RALF1 treatment (Fig 2A), the RALF1-induced accumulation of EBP1 protein was more significant in Col-0 than in *fer-4* (Fig 2A), indicating that RALF1 induces EBP1 protein accumulation in a FER-dependent manner.

At three levels (*EBP1* mRNA transcription, *EBP1* mRNA stability, and *EBP1* mRNA translation), we investigated how RALF1 peptide elevates EBP1 protein content (Fig 2B–2H, S7A and S7B Fig). We first investigated whether RALF1 induces EBP1 protein accumulation at the mRNA transcript level. The results of the quantitative reverse transcription PCR (qRT-PCR) analysis showed that the *EBP1* mRNA level was not significantly affected by RALF1 (S7A Fig), indicating that EBP1 protein levels are subject to tight posttranscriptional control through an unknown mechanism. Using cordycepin (CRD; an mRNA transcription inhibitor) to stop the *EBP1* mRNA synthesis, we measured the rate of *EBP1* mRNA decay and found no significant difference in RNA decay rate with or without RALF1 treatment over 2 hours (S7B Fig). *ATHSPRO2* (a reported gene with highly unstable mRNA [48]) was used as positive control (S7B Fig). We next investigated whether RALF1 promoted *EBP1* mRNA translation by increasing its polysome profiles (Fig 2B–2G). We performed polysome profiling assays and found that the portion of polysome-bound *EBP1* mRNA was increased upon RALF1 treatment for 30 minutes in Col-0 (Fig 2B and 2C). However, in the *fer-4* mutant, the *EBP1* mRNA binding to polysomes was not increased significantly (Fig 2E and 2F), suggesting that RALF1-enhanced EBP1 mRNA translation was dependent on FER. The polysome-bound *EIF4A1* mRNA content was measured as a control, showing that RALF1 did not induce polysome binding of *EIF4A1* mRNA (Fig 2D and 2G). To gain further evidence for RALF1-induced *EBP1* mRNA translation, cycloheximide (CHX; an mRNA translation inhibitor) was used to block new protein synthesis. Western blots indicated that RALF1-induced EBP1 protein accumulation was abolished by CHX (Fig 2H). When MG132 (an inhibitor of the 26S proteasome) was used, we did not observe significant change in RALF1-induced EBP1 protein accumulation (Fig 2H). These data suggest that RALF1 affected EBP1 protein accumulation through altering the translation of *EBP1* mRNA.

### RALF1-induced accumulation of EBP1 in the nucleus

To investigate the subcellular localization of accumulated EBP1, Col-0 and *fer-4* plants were collected (with or without RALF1 treatment), and a nuclear fractionation assay was performed. Immunoblot assays were performed to measure the EBP1 protein content in both the cytoplasmic and nuclear fractions. In the cytoplasmic fractions of both Col-0 and *fer-4* plants (with or without RALF1 treatment), EBP1 was detected at a low level (Fig 2I). RALF1 treatment only slightly increased the content of EBP1 in the cytoplasm (Fig 2I). However, in the nuclear fraction, EBP1 was hardly detected in Col-0 and *fer-4* plants without RALF1 treatment (Fig 2I). Accumulation of EBP1 in the nucleus increased remarkably after RALF1 treatment in both Col-0 (lane 6) and *fer-4* (lane 8) plants (Fig 2I). However, the nuclear EBP1 level increased more in Col-0 than in *fer-4*, suggesting that RALF1-induced nuclear accumulation of EBP1 is partially dependent on functional FER (Fig 2I). We further investigated whether EBP1 accumulation in the nucleus was RALF1 specific (S7C Fig). When treated with PEP1 peptide (AT5G64900), ABA, and 1-naphthaleneacetic acid (NAA), no distinct EBP1 nuclear accumulation was detected (S7C Fig).

EBP1 is localized in the nucleus upon activation of the EGFR pathway in animals [31]. In plants, a previous work reported that EBP1–green fluorescent protein (GFP) (*At*CPR-GFP) can be detected by GFP fluorescence in the guard cells but not in other cell types, even when its expression is driven by the *35S* promoter [40]. We made overexpressing transgenic plants harboring a *35S*::EBP1-GFP construct (S7D–S7F Fig) and further obtained *fer-4*/*EBP1-GFP* hybrid plants. Consistent with the previous work [40], we observed GFP fluorescence in the *EBP1-GFP* transgenic plant guard cells (S7G Fig). However, we detected only weak GFP fluorescence in roots (Fig 2J), although we detected a high level of *EBP1-GFP* mRNA expression (S7E Fig). Driven by these results, we further visualized GFP signaling in 7-DAG *EBP1-GFP* and *fer-4*/*EBP1-GFP* seedlings (treated with or without 1 μM RALF1 peptide in 1/2 Murashige and Skoog [MS] liquid medium) (Fig 2J). Without the RALF1 peptide treatment, we observed only dim fluorescence in the roots of *EBP1*-*GFP* and *fer-4*/*EBP1-GFP* hybrid plants (Fig 2J). A strong fluorescent signal was observed in the nuclei of the RALF1-treated *EBP1-GFP* plants (Fig 2J). The nuclear localization of the EBP1-GFP fluorescent signal was verified by using the nuclear staining dye Hoechst 33258 (S8A Fig). In RALF1-treated *fer-4*/*EBP1-GFP* seedlings, the fluorescent signal in the nucleus was observed but was weaker than that in the RALF1-treated *EBP1-GFP* plants (Fig 2J). The percentage of cells having nuclear fluorescent signals in RALF1-treated *EBP1-GFP* plants was also higher than that in RALF1-treated *fer4*/*EBP1-GFP* plants (Fig 2K). However, RALF1 did not enhance GFP intensity in the roots of *35S*::*GFP Arabidopsis* plants (S8B Fig), and free GFP was not detected in plant sample expressing EBP1-GFP (S2D Fig), suggesting that the RALF1-induced fluorescence enhancement is attributed to EBP1-GFP fusion protein rather than free GFP accumulation.

To further prove that RALF1 could promote EBP1 protein accumulation in the nucleus in an FER-dependent manner, an immunofluorescence assay was performed using EBP1 antibody (S8C Fig). Without RALF1 treatment, weak fluorescence was observed both in Col-0 and *fer-4* root cells (S8C Fig). Before RALF1 treatment, we noted that EBP1 protein level was slightly higher in the *fer-4* background when compared with wild type (WT), which is briefly commented on in the Discussion section. With RALF1 treatment, the EBP1 protein was accumulated in the nucleus in Col-0 (S8C Fig). The nuclear staining dye DAPI was used to confirm the localization of the nucleus (S8C Fig). However, in the RALF1-treated *fer-4* mutant, weak fluorescence was observed in the nucleus (S8C Fig). These results suggest that RALF1 peptide promoted *EBP1* mRNA translation and protein accumulation in the nucleus in an FER-dependent manner.

### EBP1 nuclear accumulation and phosphorylation in response to the RALF1-FER signaling pathway

**Fig 3:**
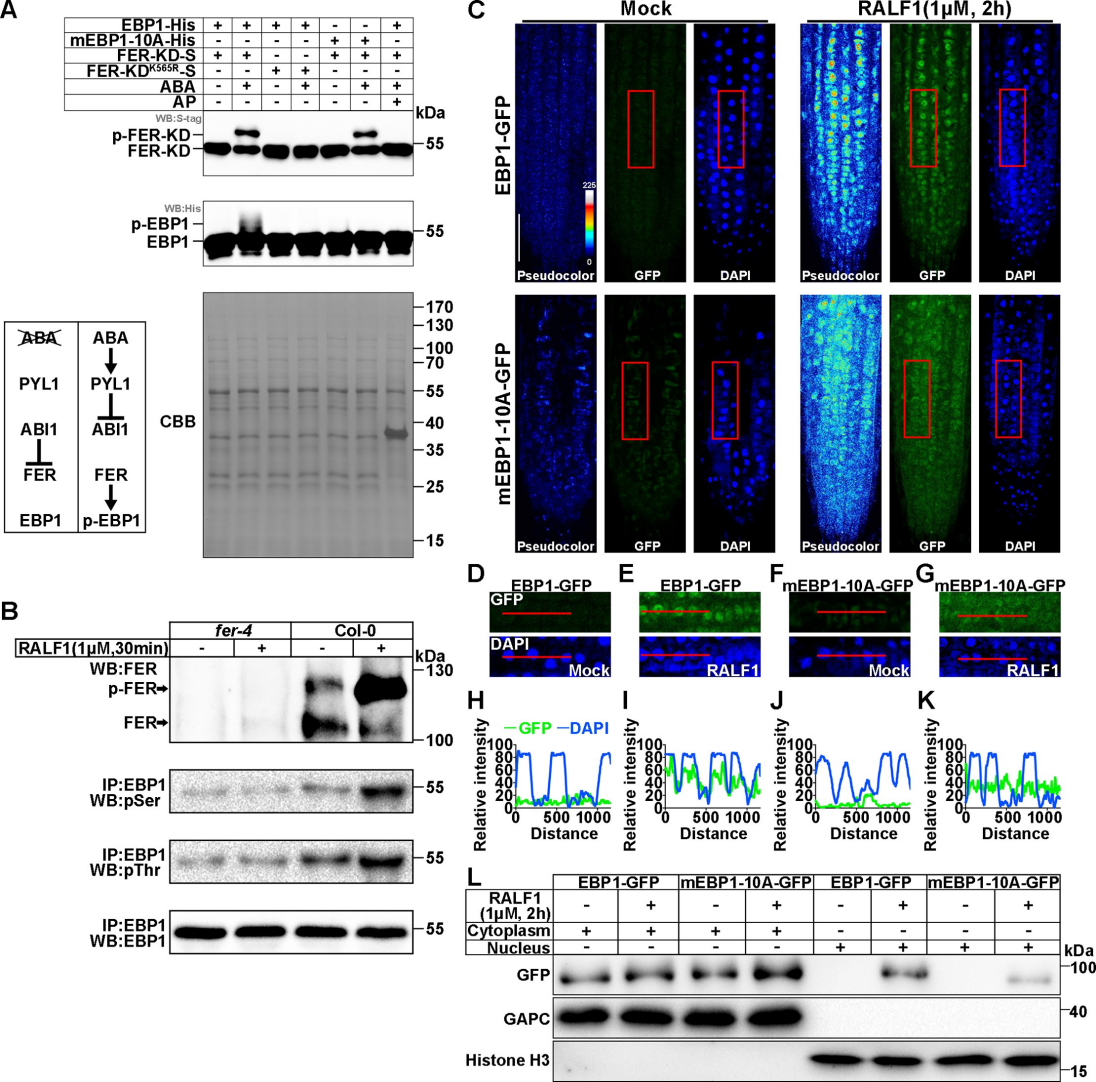
EBP1 nucleus accumulation and phosphorylation in response to RALF1-FER signaling pathway. (A) In vitro phosphorylation assay of EBP1. The phosphorylated and dephosphorylated FER-KD-S and EBP1-His are indicated. Anti-S-tag was used to detect FER-KD-S. Anti-His was used to detect EBP1-His. CBB staining is displayed as loading control. The diagram shows the skeleton of an ABA-triggered phosphorylation system. (B) Phosphorylation assay of the IP EBP1. The phosphorylation level of FER is measured by the intensity of the phosphorylated and dephosphorylated FER bands detected with FER antibody. pSer and pThr antibodies were used to detect the phosphorylated EBP1. For RALF1 treatment, 4-week-old Col-0 and *fer-4* plants were soaked in 1/2 MS liquid medium with or without 1 μM RALF1 for 30 minutes. EBP1 antibody was used to indicate loading control. We adjusted the level of total EBP1 protein to be same so that the changes in phosphorylation level of EBP1 can be easily visible. Three independent experiments of (A) and (B) were performed, and similar results were obtained. (C) Subcellular localizations of EBP1-GFP and mEBP1-10A-GFP in the root tip. Pseudocolor images show the signal intensities of the GFP signal. Color code bar indicates the relative intensities. The original pictures of GFP and DAPI signals are shown. Seedlings (with or without 1 μM RALF1 treatment for 2 hours) that were 7 DAG were used for observation. Bar = 50 μm. Five independent assays were performed, and similar results were obtained. (D-G) Close-ups of the GFP and DAPI signals in C (with red frames). (H-K) The selected areas (as indicated by the red lines in D-G) were analyzed for fluorescence intensity using ImageJ, and the yielded plot profiles are shown. All the fluorescence images were recorded using the same parameters under laser scanning confocal microscope. Three independent lines of *EBP1-GFP* and *mEBP1-10A-GFP* were analyzed, and similar results were obtained. (L) Abundance of EBP1-GFP and mEBP1-10A-GFP in the nucleus fraction. Immunoblot analyses of EBP1-GFP or mEBP1-10A-GFP in both the nuclear and nuclei-depleted soluble fractions from RALF1-treated (1 μM for 2 hours) or untreated plant. Antibody against GFP was used to detect EBP1-GFP or mEBP1-10A-GFP protein. Antibodies against GAPC or Histone H3 were used to mark cytosolic or nucleus fractions respectively. Similar results were obtained in three independent assays. Numerical data used to generate the plot in H-K are provided in S1 Data. ABA, abscisic acid; AP, alkaline phosphatase; CBB, Coomassie Brilliant Blue; DAG, day after germination; EBP1, ErbB3-binding protein 1; FER, FERONIA; FER-KD-S, FER kinase domain fused to S-tag; GAPC, cytosolic glycolytic GAPDHs; GFP, green fluorescent protein; IP, immunoprecipitated; MS, Murashige and Skoog; pSer, phosphoserine; pThr, phosphothreonine; RALF1, rapid alkalinization factor 1; WB, western blot.

As a kinase-interacting protein, EBP1 could potentially be phosphorylated by FER. A coexpression system in *Escherichia coli* was designed to examine the dynamics and the in vivo phosphorylation process. This system has been used successfully to study RLK-mediated phosphorylation reaction in *E*. *coli* [49]. Our previous work has shown that PP2C-A-type protein phosphatases (such as ABI1 and ABI2) interact with and dephosphorylate FER, thus inhibiting the kinase activity of FER [12]. It is also known that the ABA receptor pyrabactin resistance 1-like 1 (PYL1) attenuates ABI1’s inhibitory effect on FER in the presence of ABA [12]. Based on these findings, we constructed an ABA-induced in vitro phosphorylation system to investigate whether EBP1 is a phosphorylation substrate of FER (Fig 3A). We coexpressed PYL1, ABI1, FER-KD, and EBP1 (PYL1/ABI1/FER-KD/EBP1) in one *E*. *coli* strain. In parallel, we coexpressed the same proteins except for a mutant version of FER-KD that contained the Lys^565^-to-Arg mutation (FER-KD^K565R^) to abolish its kinase activity [4] as negative control. When ABA was not added to the culture, ABI1 would inhibit FER activity to avoid its phosphorylation ability toward to its substrate. After ABA was added to the culture medium, the PYL1-ABA complex interacted with and inhibited ABI1 activity, thus releasing the active FER-KD kinase. In the assay, EBP1 was fused with a His-tag, and FER-KD (or FER-KD^K565R^) was fused with an S-tag so that an immunoblot assay could be used to detect phosphorylation-related band shifts, as in our previous work [12].

First, we detected the phosphorylation status of FER-KD fused to S-tag (FER-KD-S) to ensure that this system worked. When no ABA was added, no band shift attributable to FER-KD phosphorylation was detected (lane 1) (Fig 3A). Once ABA was added, the band shift was detected (lane 2), and alkaline phosphatase (AP) dephosphorylated and eliminated band shifts in FER-KD (lane 7) (Fig 3A). This result proved that the addition of ABA activated FER-KD-S phosphorylation activity, and therefore, we investigated whether FER-KD could phosphorylate EBP1 in this system. Indeed, in the absence of ABA, no phosphorylation-dependent shift of EBP1 was detected (lane 1), whereas the addition of 50 μM ABA induced EBP1 phosphorylation (p-EBP1) (lane 2) (Fig 3A). The ABA-dependent shift of the EBP1 protein band resulted from phosphorylation, because AP dephosphorylated and eliminated this shift in the EBP1 band (lane 7) (Fig 3A). This EBP1 phosphorylation was dependent on active FER kinase, because ABA did not induce an EBP1 band shift when added into the culture medium of the cells containing the kinase-dead FER-KD^K565R^, PYL1, ABI1, and EBP1 (lane 3 and 4) (Fig 3A). To further investigate whether EBP1 was phosphorylated by FER in vivo, we immunoprecipitated the EBP1 protein from Col-0 and *fer-4* plants roots using an EBP1 antibody (Fig 3B). Because RALF1-FER pathway alters EBP1 abundance in plants, we had to adjust the total protein levels of EBP1 from different samples to be comparable so that the changes in the phosphorylation levels of EBP1 protein can be easily visible (Fig 3B). Because the mobility shift between EBP1 and p-EBP1 was small, we used phosphoserine (pSer) and phosphothreonine (pThr) antibodies to monitor the phosphorylation level of EBP1. Before RALF1 treatment, the EBP1 pSer and pThr phosphorylation levels in Col-0 were both higher than those in *fer-4* (Fig 3B). After RALF1 treatment, the EBP1 pSer and pThr phosphorylation levels were increased in Col-0 but not in the *fer-4* mutant (Fig 3B), and the FER phosphorylation level was up-regulated, as indicated by the band shift assays. Thus, the phosphorylation assays suggest that EBP1 was phosphorylated by FER kinase.

To identify the phosphorylation sites of EBP1, the phosphorylation assay shown in Fig 3A was performed at a larger scale, and EBP1 was purified and analyzed using mass spectrometry (S9 Fig). Ten EBP1 aa residues (Ser^13^, Thr^15^, Ser^16^, Thr^242^, Thr^243^, Tyr^245^, Ser^378^, Thr^379^, Ser^387^, and Ser^388^) were identified as phosphorylated in the sample containing ABA-incubated PYL1/ABI1/FER-KD/EBP1 system, but no phosphorylation was observed either in the absence of ABA or in the ABA-incubated PYL1/ABI1/FER-KD^K565R^/EBP1 system (S9 Fig). To further confirm these 10 phosphorylation sites, we simultaneously mutated all 10 identified phosphorylation sites to alanine residues (yielding mEBP1-10A) to constitutively inactivate these phosphorylation sites and tested whether mutated mEBP1-10A-His was phosphorylated by FER-KD (Fig 3A). The results showed that, although ABA activated FER-KD, mEBP1-10A-His was not phosphorylated by FER-KD (comparing lanes 5 and 6), suggesting that one or multiple of these residues may be phosphorylated by FER.

We next further analyzed the physiological roles of these 10 phosphorylation sites. Based on the domain structure of the *Arabidopsis* EBP1 protein, we analyzed the locations of the 10 phosphorylation sites of EBP1 protein (S5 Fig). We found that 7 phosphorylation sites were located at the N-terminal and C-terminal potential nuclear localization–related regions (NLRs) (S5 Fig). To investigate whether these p-EBP1 sites were critical for EBP1 to accumulate in the nucleus, we further tested the functional relevance of these 10 phosphorylated aa residues by examining RALF1-dependent nuclear accumulation of EBP1 (Fig 3C–3K). We cloned the EBP1 or mEBP1-10A sequences into a *35S* promoter-driven GFP fusion construct and obtained *EBP1-GFP* and *mEBP1-10A-GFP* (S2D Fig, S7D–S7F Fig) transgenic *Arabidopsis*. As in Fig 2J, RALF1 promoted EBP1-GFP protein accumulation in the nucleus (Fig 3C). RALF1 also promoted mEBP1-10A-GFP protein levels (S7F Fig); however, only weak fluorescence was detected in the nucleus, compared with that in unmutated EBP1-GFP plants (Fig 3C). DAPI staining and plot profile analysis showed that the enhanced fluorescence of RALF1-treated *EBP1-GFP* was mainly in the nucleus (Fig 3D–3K). We performed a nucleus fractionation assay to detect the accumulation of EBP1-GFP and mEBP1-10A-GFP in the nucleus (Fig 3L). RALF1 treatment increased the content of both EBP1-GFP and mEBP1-10A-GFP in nucleus (Fig 3L). However, a much weaker mEBP1-10A-GFP signal was detected in the nucleus when compared with EBP1-GFP (Fig 3L). Further, we simultaneously mutated the three phosphorylation sites (Ser^13^, Thr^15^, and Ser^16^, yielding mEBP1-N3A) located in the N-terminal NLR (N-NLR), the three phosphorylation sites in the middle section (Thr^242^, Thr^243^, and Tyr^245^, yielding mEBP1-M3A), the four sites located in the C-terminal NLR (C-NLR) (Ser^378^, Thr^379^, Ser^387^, and Ser^388^, yielding mEBP1-C4A), or all 10 phosphorylation sites (mEBP1-10A) to alanine residues to constitutively inactivate these phosphorylation sites and incorporated these mutations in the GFP fusion constructs (S10 Fig). We used an *Arabidopsis* protoplast transfection assay to investigate whether these mutant proteins showed altered localization in response to exogenous RALF1 peptide (S10 Fig). Without RALF1 treatment, the WT EBP1-GFP and all mutated versions showed similar nuclear localization (S10A and S10B Fig). After RALF1 treatment for approximately 1 hour, the percentage of cells with nuclear localization of WT EBP1-GFP was increased to 29.2% (approximately twice more as compared to those before RALF1 treatment, *P* < 0.001) (S10A and S10B Fig). However, mEBP1-C4A-GFP and mEBP1-10A-GFP did not show significant changes (*P* > 0.05) in nuclear localization upon RALF1 treatment (S10B Fig). mEBP1-N3-GFP and mEBP1-M3-GFP showed approximately 63.5% (*P* < 0.001) and 77.7% (*P* < 0.001) increases in cells with nuclear localization after RALF1 treatment, respectively (S10B Fig). These data suggest that these 10 phosphorylation sites might affect RALF1-induced EBP1 nuclear accumulation to different extents. The C-NLR-associated phosphorylation sites (Ser^378^, Thr^379^, Ser^387^, Ser^388^) seem to be more important for EBP1 nuclear accumulation, consistent with their close proximity to the NLS (S5 Fig). We further examined the effect of mutations in all 10 phosphorylation sites on nuclear accumulation of EBP1 by comparing the average nucleus/cytoplasm (N/C) fluorescence ratios of EBP1-GFP and mEBP1-10A-GFP upon RALF1 treatment (S10C–S10E Fig). The N/C fluorescence ratios were 1.63 for EBP1-GFP and 1.18 for mEBP1-10A-GFP, showing a significant difference (*P* < 0.001) (S10C–S10E Fig).

### *ebp1* mutants, similar to *fer*, display defects in cell growth in different cell types

FER plays a critical role in cell growth, and its function may vary in different cell types as well as in distinct hormonal responses [50]. To examine whether EBP1 functions in the FER pathway, we sought to directly compare the phenotypic defects in *ebp1* and *fer* mutants. To this end, we obtained three *ebp1* mutant lines and *EBP1-overexpression* (*EBP1-OE*) lines (S11 Fig), as described in the Methods.

One of the phenotypic changes in *fer* mutants is shorter hypocotyls when grown in the dark [7,8]. We compared the hypocotyl lengths of dark-grown Col-0, *fer-4*, and *ebp1* mutants. The three *ebp1* mutants, similar to *fer-4*, all showed shorter hypocotyls than Col-0 (*P* < 0.001), whereas the *EBP1-OE* lines showed longer hypocotyls than the WT (S12A and S12B Fig, *P* < 0.01). We stained the hypocotyls using propidium iodide (PI) (S12C Fig) and found that the cells in the *ebp1* mutant lines were shorter than those in the WT plants (S12D Fig, *P* < 0.001).

**Fig 4:**
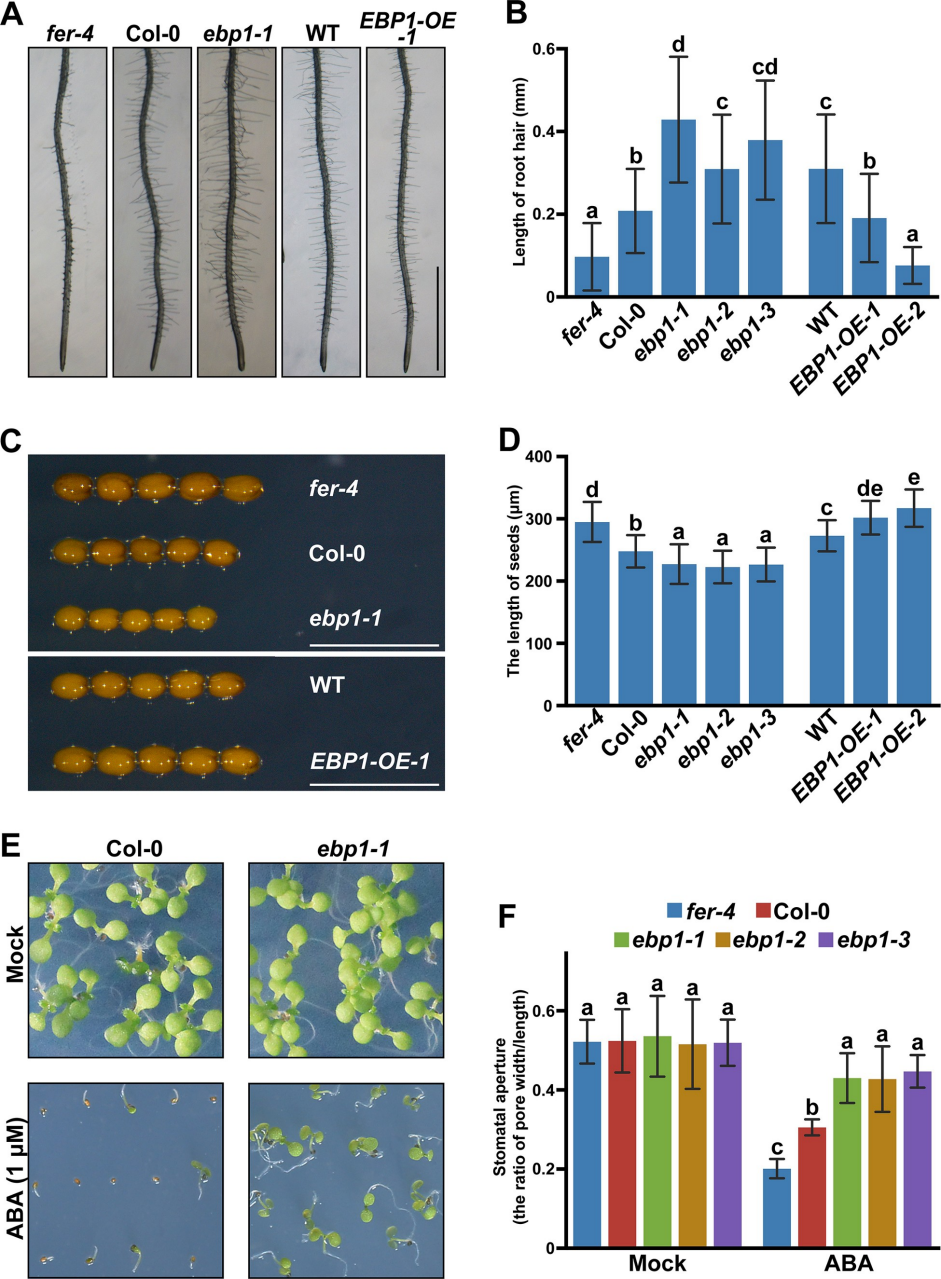
Comparison of *ebp1* and *fer4* mutant phenotypes in cell growth and ABA response. Phenotype (A, bar = 1 mm) and statistical analysis (B) show that *ebp1* plants have longer root hairs, whereas *EBP1-OE* plants show shorter root hairs as compared to the WT. At least 34 root hairs’ length of each plant line were measured. Phenotype (C, bar = 0.5 mm) and statistical analysis (D) show that *ebp1* mutants plants have smaller seeds, whereas *EBP1-OE* plants show larger seeds as compared to the WT. For phenotypes of mutants and overexpressing lines, only one of each line (*ebp1-1* and *EBP1-OE-1*) was chosen for displaying photos. Three lines of *ebp1* and two lines of *EBP1-OE* were used in statistical analysis. At least 21 seeds were measured in each plant line. (E, F) *ebp1* mutants are less sensitive to exogenous ABA as compared to WT. The images show 9-DAG plants grown on 1/2 MS agar medium supplemented with 0 μM ABA (Mock), or 1 μM ABA. (F) Stomatal aperture analysis in *fer-4*, Col-0, and *ebp1* mutants before and after ABA treatment. The ratio of stomatal pore width/length was used to represent the aperture. The pore width and length were measured using ImageJ software. Ten guard cells of each plant lines were measured for statistical analysis. For phenotypes of mutants and overexpressing lines, only one of each line (*ebp1-1* and *EBP1-OE-1*) was chosen for displaying. Data points are means +/− SD. Values with different letters are significantly different from each other, tested by one-way ANOVA. All assays were performed in three independent experiments, and similar results were obtained. Numerical data used to generate the plot in B, D, and F are provided in S1 Data. ABA, abscisic acid; DAG, day after germination; *EBP1*, *ErbB3-binding protein 1*; *EBP1-OE*, *EBP1-overexpression*; MS, Murashige and Skoog; WT, wild type.

Another hallmark of FER function is regulation of ROS-mediated root hair growth through the GEF-ROP/RAC pathways [5]. The *fer* mutants have shorter root hairs because of a reduced response to auxin. The *ebp1* mutants, unlike *fer* mutants, showed longer root hairs than those of the WT (Fig 4A and 4B, *P* < 0.001). In contrast, the root hairs in the *EBP1-OE* lines were shorter than those of the WT (Fig 4A and 4B, *P* < 0.001).

FER plays a negative role in the control of seed size, with the *fer-4* mutant producing larger seeds than those of the WT [51]. We measured the seed sizes of *ebp1* and *EBP1-OE* and found that *ebp1* had smaller seeds (*P* < 0.01) than those of the WT, whereas *EBP1-OE* seeds were larger (*P* < 0.01) (Fig 4C and 4D). The positive role of EBP1 in seed size control is also supported by a recent study showing that overexpression of maize EBP1 in transgenic *Arabidopsis* increases seed size [42].

*FER* is expressed in guard cells and plays an important role in ABA-induced stomatal closure through the GEF-ROP/RAC signaling network [12,17]. Previous research has shown that EBP1 is expressed in guard cells [40], suggesting that *EBP1* may also play a role in guard cells. When seedlings were assayed for greening response to ABA, *ebp1* mutant seeds germinated more rapidly than the WT and led to a higher percentage of green seedlings, indicating a lower sensitivity to ABA (Fig 4E). We further tested the stomatal response to ABA in *ebp1* and WT plants and found that *ebp1* mutants were less sensitive to ABA than Col-0 seedlings (Fig 4F, *P* < 0.05).

### EBP1 plays negative roles in RALF1 peptide signaling

**Fig 5:**
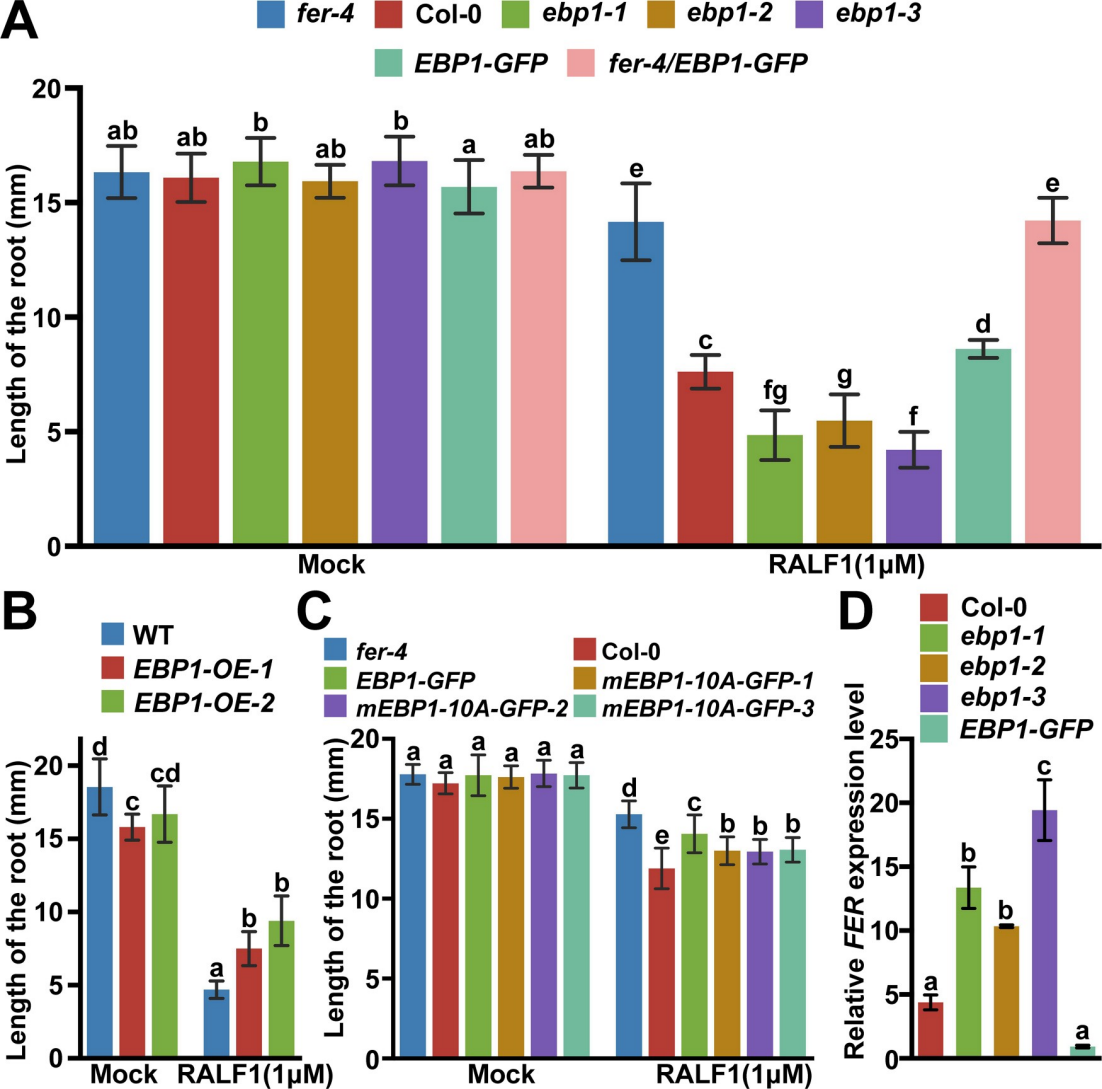
EBP1 inhibits the RALF1 peptide response. (A) *ebp1* mutants are hypersensitive, whereas *fer-4*, *EBP1-GFP* and *fer-4/EBP1-GFP* plants are less sensitive to RALF1 peptide in root growth assay as compared to the WT. *n* = 10. (B) *EBP1-OE* lines (*rdr6* background) are less sensitive to RALF1 than the WT in root elongation assay. *n* = 10. (C) *EBP1-GFP* is less sensitive to RALF1 than Col-0, whereas *mEBP1-10A-GFP* lines show more sensitivity to RALF1 than *EBP1-GFP* but less sensitivity than Col-0. *n* = 9. Three independent lines of *mEBP1-10A-GFP* were gained respectively, and similar results were obtained. Root growth assays were performed in four independent experiments, and similar results were obtained. (D) *FER* mRNA levels in *ebp1* mutants and *EBP1-GFP* lines. Quantification of *FER* mRNA levels relative to *Actin* was done using qRT-PCR. Data points are means +/− SD of two technical replicates. Values with different letters are significantly different (*P* < 0.05) from each other, tested by one-way ANOVA. Similar results were obtained in two independent experiments. Numerical data used to generate the plot are provided in S1 Data. EBP1, ErbB3-binding protein 1; *EBP1-OE*, *EBP1-overexpression*; qRT-PCR, quantitative reverse transcription PCR; RALF1, rapid alkalinization factor 1; WT, wild type.

The above phenotypic comparisons implicated EBP1 in FER-regulated cellular activities. Next, we performed RALF1 peptide response assays [20] to compare directly if *ebp1* mutant and *fer* mutant displayed related phenotypes. First, we investigated root elongation inhibition in response to RALF1 [23] in plants of various genotypes, including *fer-4*, *ebp1*, *EBP1-GFP* and *fer-4/EBP1-GFP* (Fig 5A). Consistent with previous research [20], *fer-4* was less sensitive to RALF1 than the WT (Fig 5A, *P* < 0.001). Compared to the mock treatment, Col-0 root length was reduced approximately 52.6% in the presence of RALF1 peptide (*P* < 0.001), whereas *fer-4* was reduced approximately 13.3% (Fig 5A, *P* < 0.001). The *ebp1* was more sensitive than the WT to RALF1 treatment, as reflected by more severe reduction in root elongation (71.1% for *ebp1-1*, 65.6% for *ebp1-2*, and 75.0% for *ebp1-3*) (Fig 5A, *P* < 0.001). In contrast, *EBP1-GFP* was less sensitive to RALF1 than the WT (45.1% inhibition) (Fig 5A, *P* < 0.05). An additive phenotype of RALF1 insensitivity in *fer-4/EBP1-GFP* (13.1% inhibition) compared to *EBP1-GFP* was observed (Fig 5A, *P* < 0.001). However, no significant additive RALF1 insensitivity was observed in *fer-4/EBP1-GFP* relative to *fer-4* (Fig 5A, *P* > 0.05), suggesting that EBP1 played roles downstream of the RALF1-FER signaling pathway, and EBP1 inhibited RALF1 response in roots. The RALF1 responses in the other *EBP1-OE* lines (*rdr6* background) and *rdr6* control were analyzed (Fig 5B), and we found RALF1 had lower sensitivity in the *EBP1-OE* lines than that in the *rdr6* control (Fig 5B, *P* < 0.001). The *mEBP1-10A-GFP* lines also showed lower sensitivity than that of Col-0 (Fig 5C, *P* < 0.05), albeit to a lesser extent than *EBP1-GFP* (Fig 5C, *P* < 0.05). Meanwhile, we crossed the *ebp1-1* mutant with plants expressing *EBP1-GFP* or *mEBP1-10A-GFP* (S13 Fig) and performed root growth response to RALF1. *EBP1-GFP* rescued the *ebp1-1* mutant in the root growth assay (S13 Fig, *P* > 0.05), showing the functionality of the EBP1-GFP fusion protein. However, compared with EBP1-GFP, mEBP1-10A-GFP only partially complemented the *ebp1-1* mutant phenotype in the same assay (S13 Fig, *P* < 0.001), again suggesting that the phosphorylation sites are important for EBP1’s role in plants.

A study shows that some RALFs (e.g., RALF23, RALF33, and RALF34) inhibit flg22- or 18 amino acid fragment of bacterial elongation factor Tu (elf18)-triggered ROS bursts [16]. Here, we checked the RALF1 response in *ebp1* mutant plants using the similar assays. In response to flg22, the WT and *ebp1* mutant plants show a similar level of ROS burst that has been reduced in the *fer-4* mutant (S14A Fig). When treated with flg22 and RALF1 combined, the ROS burst in the WT was partially inhibited (S14A Fig, *P* < 0.001), suggesting that RALF1 suppressed flg22-triggered ROS burst. In *ebp1* mutant lines, RALF1 more severely impaired the flg22-triggered ROS burst, again suggesting that *ebp1* mutants were more sensitive to the RALF1 peptide than Col-0 (S14A Fig, *P* < 0.05).

The activation of mitogen-activated protein kinase (MAPK) cascade is another RALF response indicator [52]. We found that RALF1-induced MAPK activities were higher in *ebp1* mutant lines than in Col-0, further suggesting that *ebp1* mutants are more sensitive to RALF1 peptide (S14B Fig).

RALF1 induces rapid alkalinization of culture media [18] via the active FER kinase receptor by inhibiting AHA2 activity [20]. Thus, we measured pH variations in culture medium before and after the addition of RALF1 (S14C and S14D Fig). Within 10 minutes, RALF1 significantly increased the medium pH in the WT plants (S14D Fig), similar as reported earlier [18]. The pH values were approximately 7.05, 6.27, 8.22, 7.62, and 7.51 in Col-0, *fer-4*, *ebp1-1*, *ebp1-2*, and *ebp1-3*, respectively, after 10 minutes (S14D Fig). Because of the subsequent H^+^ efflux, the medium pH started to decrease, but the medium pH values of the *ebp1* mutants were still higher than that of Col-0, and the medium pH of the *fer-4* plants was the lowest (S14D Fig), suggesting that RALF1 has strong effects on *ebp1* mutants, in contrast to Col-0 and *fer-4*. As EBP1 affected many aspects of FER function (especially several nonnuclear RALF1 responses, including RALF1-mediated inhibition of flg22-triggered ROS, MAPK phosphorylation, and proton secretion), we tested the possibility that EBP1 might affect *FER* gene expression. We examined *FER* mRNA levels in *ebp1* mutants and *EBP1-OE* and found that *FER* was up-regulated in *ebp1* mutants and was down-regulated in *EBP1-OE* (Fig 5D). Taken together, these assays suggested that EBP1 negatively regulates RALF1 response.

### EBP1 associates with gene promoters in response to RALF1 signaling

**Fig 6:**
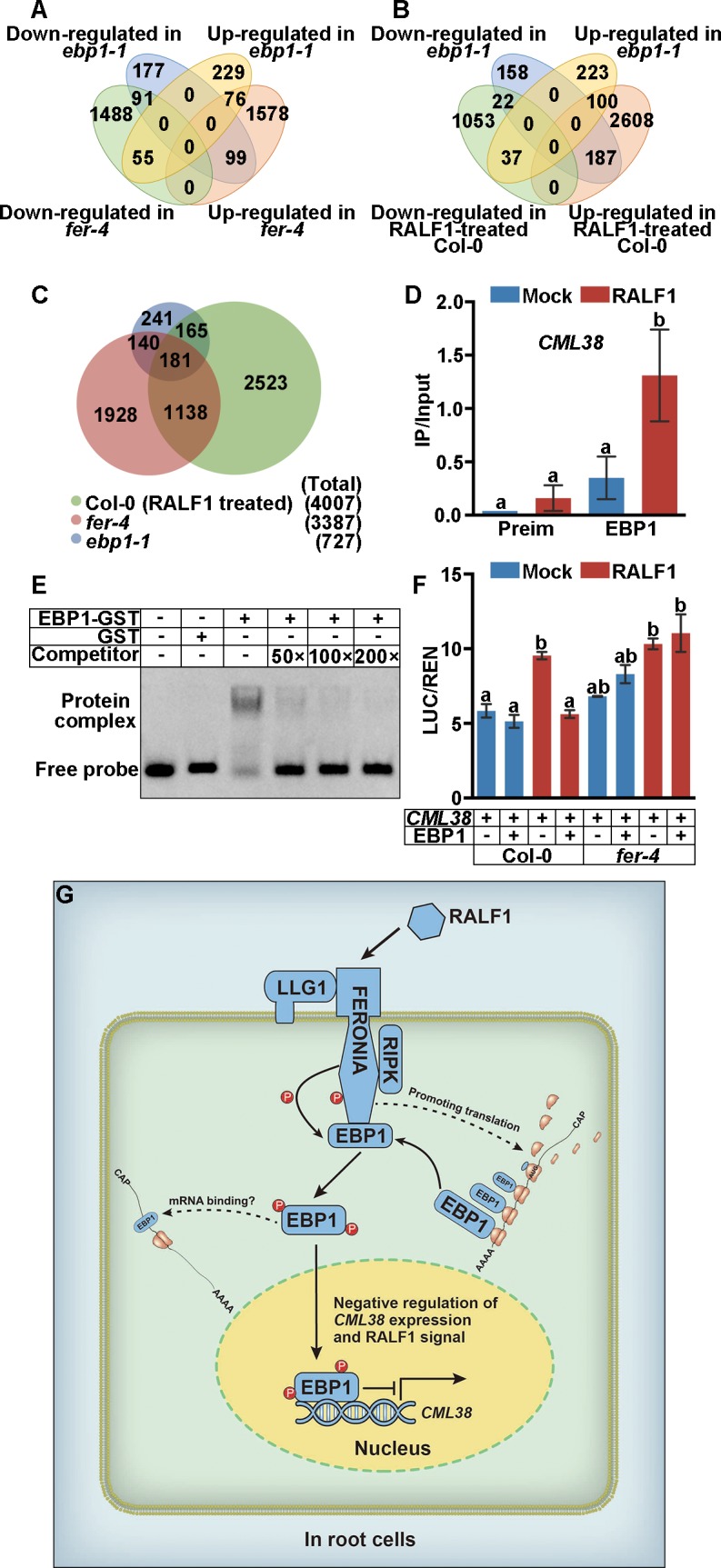
RALF1-FER-EBP1 signaling pathway regulates gene expression. (A, B) Venn diagram shows the overlapping numbers of up- or down-regulated genes in *ebp1-1* and *fer-4* mutant (A), or *ebp1-1* and RALF1-treated Col-0 (B), comparing with Col-0 without RALF1 treatment. (C) Venn diagram shows the total and overlapping numbers of EBP1-, FER-, and RALF1-regulated genes. (D) *CML38* promoter was immunoprecipitated by EBP1 via ChIP assay. ChIP-qPCR results were quantified by normalization of the EBP1-IP signal with the corresponding Input signal (IP/Input). RALF1 treatment (1 μM) was performed for 2 hours on 7-DAG Col-0. Preimmune serum (“Preim”) was used for negative control. Data shown are representative of three independent experiments with similar results. (E) Competitive EMSA shows interaction of EBP1 with the FITC-labeled “CCACGTC” DNA directly. Unlabeled “CCACGTC” motif was used as the competitor. (F) Dual-LUC shows relative reporter activity (LUC/REN) of indicated genotypes (Col-0 or *fer-4*). RALF1 treatment (0.1 μM RALF1) condition, *proCML38*::LUC (“*CML38*”), and EBP1 protein expression are shown. Quantification of LUC relative to REN levels (“LUC/REN”) was performed on three replicates. Data shown in (E) and (F) are representative of four independent experiments with similar results. Data points show means +/− SD. Values with different letters are significantly different (*P* < 0.05) from each other, tested by one-way ANOVA. (G) A working model of EBP1 function in the regulation of root cell growth in response to peptide signals. In root cells, FER-LLG1 complex interacts with RALF1 peptide. FER somehow promotes EBP1 protein translation. FER also directly interacts with and phosphorylates EBP1. The phosphorylated EBP1 accumulates in the nucleus, where it regulates gene expression. Numerical data used to generate the plot in D and F are provided in S1 Data. ChIP, chromatin immunoprecipitation; DAG, day after gemination; Dual-LUC, transient transcription dual-luciferase assay; EBP1, ErbB3-binding protein 1; EMSA, electrophoretic mobility shift assay; FER, FERONIA; FITC, fluorescein isothiocyanate; GST, glutathione S-transferase; LUC, luciferase; qPCR, quantitative PCR; RALF1, rapid alkalinization factor 1; REN, Renilla luciferase; RIPK, RPM1-induced protein kinase.

EBP1 is a DNA- [33] and RNA-binding [34,35] protein. Thus, we wondered whether the RALF1-FER pathway regulates the association of EBP1 with DNA to control gene transcription after its accumulation in the nucleus. First, RNA sequencing (RNA-seq) was performed to screen EBP1-regulated genes. We prepared RNA-seq libraries using RNAs isolated from 7-DAG Col-0 (with or without RALF1 treatment for 2 hours), *ebp1-1*, and *fer-4* seedlings with three biological replicates (Fig 6A–6C, S15 Fig). Comparing Col-0 and *ebp1-1*, we found 367 genes with lower transcript levels and 360 genes with higher transcript levels in the *ebp1-1* mutant plant (Fig 6A and 6C). These affected genes mainly function in signal transduction (142 genes), transcription events (78 genes), and stress responses (S15A Fig). In *fer-4*, 3,387 genes were affected, with 1,634 and 1,753 genes showing lower or higher mRNA levels than Col-0, respectively (Fig 6A and 6C). RALF1 treatment affected approximately 4,007 genes (1,112 genes with lower and 2,895 genes with higher mRNA levels than control) in Col-0 background (Fig 6B and 6C). Using DAVID Functional Annotation tools [53], we also analyzed the functional annotations (*P* < 0.001) of differentially expressed genes between *ebp1-1* and *fer-4* (*ebp1-1* versus *fer-4*) and among *ebp1-1*, *fer-4*, and RALF1-treated Col-0 (*ebp1-1* versus *fer-4* versus RALF1-treated Col-0) (S15B and S15C Fig). Most of the genes altered in *ebp1-1* versus *fer-4* were related to signal transduction (84 genes), transcription events (41 genes), and stress responses (S15B Fig). Most of the genes altered in *ebp1-1* versus *fer-4* versus RALF1-treated Col-0 played roles in the transcription process (30 genes) or transcription regulation events (31 genes) (S15C Fig). These results suggested that the EBP1 signaling pathway is related to the RALF1-FER pathway by regulating a subset of overlapping genes.

As a nuclear-localized protein, EBP1 might directly bind to and regulate gene expression. Through RNA-seq analysis, we found that 181 overlapping genes were simultaneously affected among *ebp1-1*, *fer-4*, and RALF1-treated Col-0 (Fig 6C). We speculated that target genes are directly bound by EBP1 and regulated by the RALF1-FER-EBP1 signaling pathway. We tested this idea by examining some of these overlapping genes or related genes that have been reported to be regulated by RALF1 [20,54]. Using chromatin immunoprecipitation (ChIP)–quantitative PCR (qPCR) assay (with RALF1-treated Col-0 seedlings), we screened 17 potential genes (see the Methods) in the context of the information from RNA-seq. We found that DNA fragments from four genes (−213 to +117 of *CML38* gene; −1113 to −844 of *CKX4* gene; −1593 to −1195 of *ERF1B* gene; −2675 to −2397 of *SAUR9* gene) were immunoprecipitated by EBP1 antibody, whereas the adjacent DNA regions were not (S16A–S16H Fig). We further found that RALF1 treatment enhanced the EBP1 association with these four target genes (Fig 6D, S16I–S16K Fig). Notably, *CML38* was recently revealed as a downstream target gene of the RALF1-FER signaling pathway, as the *cml38* mutant becomes insensitive to RALF1 peptide in root elongation assay [54]. Thus, we further analyzed the *CML38*-detailed motif that was regulated by EBP1 using a candidate DNA screen strategy. Fortunately, we found a CCACGTC motif (−201 to −194 of *CML38*) that was located in the EBP1-immunoprecipitated DNA fragments of the *CML38* gene (−213 to +117) and further confirmed that this motif was bound directly by EBP1 protein in vitro using an electrophoretic mobility shift assay (EMSA) (Fig 6E). For performing a transient transcription dual-luciferase assay (Dual-LUC), *proCML38*::LUC (combined with the luciferase [LUC] reporter gene fused to the *CML38* promoter containing the CCACGTC motif) and *35S*::*EBP1* vectors were constructed to express *proCML38*-derived LUC and EBP1 protein, respectively, as described in Methods. Using this LUC assay, we confirmed that EBP1 suppressed *CML38* transcription in the protoplasts isolated from Col-0 plants in a RALF1-dependent manner (Fig 6F). *proCML38*::LUC and *35S*::EBP1 were cotransferred into *ebp1-1* mutant, and decreased *proCML38*::LUC activity was shown after RALF1 treatment (S17 Fig). In the *fer-4* mutant, lower suppression levels were detected with and without RALF1 treatment (Fig 6F). We performed qRT-PCR assay to measure *CML38* expression levels in *ebp1* mutant and in plants overexpressing *EBP1-GFP*, respectively, in response to RALF1 (S18A Fig). Without RALF1 treatment, *CML38* level is higher in *ebp1* mutants as compared to the Col-0 (S18A Fig). After RALF1 treatment, *CML38* expression was up-regulated in *ebp1* but down-regulated in *EBP1-GFP* when compared with Col-0 (S18A Fig), suggesting that EBP1 inhibits *CML38* gene expression. Taken together, these data suggest that EBP1 suppressed *CML38* mRNA expression in response to RALF1. We further detected the relative mRNA expression levels of *CKX4*, *ERF1B*, and *SAUR9* (S18B–S18D Fig). Consistent with the RNA-seq data, *CKX4*, *ERF1B*, and *SAUR9* showed up-regulated expression in the *fer-4* mutant (S18B–S18D Fig). The expression of *ERF1B* and *SAUR9* was also up-regulated in the *ebp1* mutants (S18C and S18D Fig), whereas *CKX4* was down-regulated in *ebp1* mutants (S18B Fig).

## Discussion

The mechanisms by which RLK regulates mRNA translation in plants cells are largely unknown. The NIK-RPL10-LIMYB pathway may be the only example in which an RLK (e.g., nuclear shuttle protein-interacting receptor-like kinase [NIK]) may regulate the translation process in response to viral infection in *Arabidopsis* and tomato. The receptor kinase NIK can interact with the ribosomal protein RPL10 and redirect RPL10 to the nucleus [55]. In the nucleus, RPL10 interacts with L10-interacting MYB domain-containing protein (LIMYB) to down-regulate the expression of genes encoding subunits of the translational machinery, leading to global translation suppression [56]. In this study, we identified a new regulatory mechanism in which RALF1-FER promotes translation of *EBP1* mRNA. A previous study has shown that EBP1 regulates organ growth in a dose-dependent manner [41], suggesting that the protein abundance of EBP1 is critical for its function and that EBP1 protein levels must be strictly regulated to maintain an optimal level. Our results have shown that, although *EBP1* mRNA was transcribed in the absence of RALF1 treatment (S6H and S6K Fig), it was translated at a very low level in the root tip (Fig 2J). The RALF1-FER signal triggered rapid protein synthesis of EBP1 via increasing *EBP1* mRNA translation efficiency (Fig 2). This phenomenon is reminiscent of a transcription–translation feedback loop (TTFL), a mechanism that has been well defined in circadian rhythm control [57] but has rarely been reported in receptor-mediated signaling pathways. The exact mechanism by which RALF1-FER promotes *EBP1* mRNA translation remains unclear. Although we focused on a specific target EBP1 in this report, we cannot exclude the possibility that RALF1-FER may regulate the translation machinery to promote global translation in the cell. In any case, our work provides an example showing that regulation at the level of protein translation may serve as a critical mechanism in signal transduction regulation.

In addition to regulation on translation rate by RALF1-FER pathway, EBP1 protein may be subjected to other control mechanisms to maintain the optimal levels. One such mechanism may involve factors that control the protein stability. An intriguing finding in this study was that EBP1 protein content was higher in the *fer-4* mutant than the WT plants under normal conditions. This seems to contradict the major results that RALF1-FER promotes EBP1 translation, as discussed above. We interpret this result in two possible ways. First, FER may regulate EBP1 protein stability via an unknown mechanism. We performed an in vitro protein degradation assay (S19A Fig) [58] and found that EBP1-His protein was more stable when incubated with total protein extract from *fer-4* mutant than with Col-0 extract. Furthermore, mEBP1-10A-His was more stable than EBP1-His when incubated with total protein extract from Col-0 in the protein degradation assay (S19B Fig). These results suggest that FER might reduce EBP1 protein stability. Secondly, in view of multiple roles of EBP1 in the ABA and salt stress response [44], we suggest that EBP1 protein levels may respond to other environmental cues, such as biotic and abiotic stress conditions. As *fer* mutants show stress phenotypes (such as enhanced ABA response), altered stress responses in *fer* mutants may also regulate EBP1 protein levels.

The complexity of the control mechanisms for EBP1 protein abundance in plant cells corresponds to the multifunctional nature of EBP1. We propose here some possible reasons that may explain the observations that EBP1, like FER, plays different, and in some cases opposite, roles in cell growth in different organs. Firstly, some other *Cr*RLK1L family members, such as ANX1 and CVY1, can interact with EBP1 but result in different consequences when compared with FER functions. There are known examples showing that *Cr*RLK1L members play opposite roles in specific tissues [59]. The interaction between other *Cr*RLK1Ls with EBP1 may also explain the finding that low levels of EBP1 were still detected in the nuclear fraction in RALF1-treated *fer-4* roots (Fig 2I). Secondly, in addition to the response to RALF1 in root growth regulation [20], FER also responds to other RALF family peptides (such as RALF17, RALF23, and RALF33) to regulate different or opposite cellular activities [16]. This assumption is further supported by studies showing that one *Cr*RLK1L can sense distinct RALF members to fulfill an opposite function [60,61].

During signal transduction, RLKs often directly interact with proteins in the plasma membrane or cytoplasm [62]. The mechanisms by which RLKs regulate nuclear events often involve a number of steps through the cytoplasm. BR insensitivity 1 (BRI1)/BRI1-associated receptor kinase 1 (BAK1) RLKs regulate Brassinazole resistant 1 (BZR1)/*bri1*-EMS-suppressor 1 (BES1) phosphorylation status and their accumulation in the nucleus through the *bri1*-suppressor 1 (BSU1)-BR insensitive 2 (BIN2) phosphorylation cascade, serving as a good paradigm [62]. In contrast to this paradigm, however, we found that FER may directly phosphorylate and enhance the nuclear accumulation of EBP1, a transcriptional regulator, in response to RALF1 peptide (Fig 3). We suggest that this novel mechanism of RLKs directly interacting with and phosphorylating a nuclear-cytoplasmic shuttling protein might represent a rapid and effective strategy in response to extracellular signals to control nuclear events, although similar mechanisms have rarely been identified in RLK regulation. In the future, experimental work needs to be done at structural and biochemical levels to address the question of how FER in plants and EGFR in animals share a similar substrate, EBP1, to transmit the signal from the cell surface to the nucleus.

As a nuclear-localized protein, EBP1 may bind to and thus regulate gene transcription in the nucleus. In this study, we showed that EBP1 binds to the promoter region and inhibited the expression of *CML38*, which has been shown to play a role in RALF1-induced inhibition of root elongation [54]. When *CML38* function is disrupted in *Arabidopsis* plants, RALF1 peptide fails to inhibit root growth [54]. However, *CML38* may not be sufficient to count for all RALF-regulated cellular activities. Indeed, except for *CML38*, EBP1 has many other targets (e.g., *CKX4*) that may be relevant in RALF1-regulated processes such as extracellular alkalinization. Additionally, mutations in EBP1 may affect the expression of some signaling components in the RALF1-FER pathway. In support of this assumption, we found that *FER* was up-regulated in *ebp1* mutants but was down-regulated in *EBP1-OE* (Fig 5D). The altered level of *FER* expression may thus, at least in part, explain why *ebp1* plants showed altered responses to RALF1 in nonnuclear events, such as the RALF1-mediated inhibition of flg22-triggered ROS, MAPK phosphorylation, and proton secretion (S14 Fig). Further work is needed to identify EBP1 target genes at the whole-genome level by, for example, ChIP sequencing (ChIP-seq) assays to further address the broad function of EBP1. At the posttranscriptional level, EBP1 has been identified as a part of RNP complexes and can bind to RNA directly in previous studies [34,35]. One example showed that EBP1 binds to mRNA and regulates its translation [39]. In the plant kingdom, whether EBP1 also regulates RNA-related events such as mRNA translation, mRNA alternative splicing, or mRNA decay remains an open question.

## Methods

### Plant materials

The *ebp1-1* (SALK_030408), *ebp1-2* (SALK_052695), and *ebp1-3* (CS854731) T-DNA insertion mutants were obtained from the Salk Institute (http://signal.salk.edu). All three *ebp1* mutants were confirmed regarding their T-DNA insertion locations using genomic DNA PCR amplification (S11A Fig), and the exact insertion sites of *ebp1* lines were identified by sequencing (S11B Fig). The primer sequences used to identify the *ebp1* mutants are shown in S1 Table. The T-DNA was located after the 976th bp in EBP1 CDS (inside the eighth exon) in *ebp1-1*, behind the 915th bp in EBP1 CDS (inside the seventh exon) in *ebp1-2* (S11B Fig), and after the 2,293th bp in EBP1 genomic sequence (inside the eighth intron) in *ebp1-3* (S11B Fig). Immunoblot assay was performed to confirm that all three *ebp1* mutants lacked a detectable level of EBP1 protein (S11C Fig).

The *35S*::EBP1-GFP and *35S*::mEBP1-10A-GFP were constructed using pMD1-GFP vector for obtaining transgenic *Arabidopsis* in Col-0 background. The *35S*::EBP1 (*EBP1-OE*) was constructed using pBI121 vector for obtaining *EBP1-OE* in *rdr6-11* background to reduce the transgene-induced gene silencing [63]. We confirmed the *EBP1* was overexpressed in two transgenic lines, *EBP1-OE-1* (about 7.5 times to WT) and *EBP1-OE-2* (about 10 times to WT), using real-time RT-PCR (S11D Fig). The primer sequences used in this section are provided in S1 Table.

### Plant growth conditions

For plant culture on the agar plates, *Arabidopsis thaliana* seeds were sterilized and then vernalized at 4°C for 3 days before being grown on 1/2 MS with 0.8% (w/v) sucrose solidified with 1% (w/v) agar (A7002, Sigma-Aldrich). For hypocotyl elongation assay, *Arabidopsis* was grown in the agar plates in vertical position in complete darkness in 23°C for 5 days. For ABA treatment assay, *Arabidopsis* was grown on 1/2 MS medium supplemented with ABA in indicated concentrations.

### Protein purification and EBP1 antibody production

The GST-tagged EBP1 protein was purified as described in the manufacturer’s manual using Pierce Glutathione Agarose (16102, Thermo Fisher Scientific, USA). The 6×His-tagged EBP1 and 6×His-tagged RALF1 protein [23] were purified as described in the manufacturer’s manual, using Ni-NTA Purification System (R901-15, Invitrogen, USA).

For EBP1 antibody production, purified EBP1-His protein was used as the antigen to inject (S2C Fig). A 1-month-old ICR mouse (SLAC laboratory animal) was injected with 50 μg EBP1-His protein emulsified with Complete Freund’s adjuvant (F5881, Sigma-Aldrich). Two weeks later, 50 µg EBP1-His protein emulsified with Incomplete Freund’s adjuvant (F5506, Sigma-Aldrich) was injected into the ICR mouse and then once again in the next week. The serum of the immunized mouse was obtained as EBP1 antibody for immunoblot detection. We tested and ensured that the EBP1 antibody was specific by an immunoblot, using protein extracts from *ebp1* mutant lines (S11B Fig), *EBP1-GFP* (S2D Fig), and *EBP1-FLAG* transgenic plants (S2E Fig). To separate the native EBP1 and EBP1-FLAG protein clearly, 20% (V/V) glycerol was added into the PAGE gel, and the electrophoresis was performed for 15 hours under low voltage (60 V).

### Y2H assay

The Y2H assay was performed as described [64]. The coding sequences of FER-KD were fused in-frame with the GAL4 DNA-binding domain of the bait vector pGBKT7. The other *Cr*RLK1Ls subfamily members were constructed into pGBKT7 as described in our previous work [23]. The coding sequence of EBP1 was fused in-frame with the GAL4 DNA-activating domain of the prey vector pGADT7. The bait plasmid FER-BD and the prey plasmids or cDNA library were cotransformed into the yeast strain AH109.

### BiFC assay

The ORF sequences of FER (or HERK2 as negative control) and EBP1 were amplified by PCR and cloned into plasmid pE3308 and pE3449, respectively [17]. Protoplasts were isolated from 5-week-old *Arabidopsis* rosette essentially as described [65]. Leaf strips were incubated in the cell wall–degrading enzyme solution in the dark for 3 hours. Protoplasts were purified [65] and transfected with 20 μg of plasmid DNA and an equal volume of PEG solution. The transfected protoplasts were incubated in the dark at 23°C for 16 hours to allow expression of the BiFC proteins.

### GST pull-down assay

Recombinant FER-KD-His protein was incubated overnight at 4°C with GST beads coupled with GST-EBP1 in the binding buffer (20 mM HEPES [pH 7.5], 40 mM KCl, 5 mM MgCl_2_). The beads were washed five times with the TBS buffer (50 mM Tris [pH 7.5], 150 mM NaCl) and boiled in SDS-PAGE sample buffer, and eluted proteins were analyzed by immunoblot with anti-His (M20001, Abmart) or anti-GST (SC-80998, CMC) antibody.

### Co-IP assay

For performing a Co-IP assay using A/G agarose and FER-antibody, 30 μL A/G beads (20421, Thermo Fisher Scientific) were resuspended and washed three times using NEB buffer (20 mM HEPES [pH 7.5], 40 mM KCl, 5 mM MgCl_2_) before adding 8 µL anti-FER antibody [12,23] (or preimmune serum as negative control) in a total volume of 500 µL NEB buffer and incubating for 3 hours at 4°C. The antibody-beads mixture was centrifuged at 200*g* for 1 minute to remove the supernatant. For protein extraction from plants, 7-DAG seedlings were transferred from the solid medium into liquid 1/2 MS medium and preincubated for 12 hours before RALF1 treatment to avoid manipulation-related effect during plant transfer from solid to liquid medium. Then, seedlings were soaked with 1 μM RALF1 (included in the 1/2 MS liquid medium) or mock control (1/2 MS medium containing RALF1 buffer only) for 30 minutes, and then these seedlings were ground to a fine powder in liquid nitrogen and solubilized with NEBT buffer (20 mM HEPES [pH 7.5], 40 mM KCl, 5 mM MgCl_2_, 0.5% Triton X-100) containing 1 × protease inhibitor cocktail (78430, Thermo Fisher Scientific) and 1 × phosphatase inhibitor (78420, Thermo Fisher Scientific) and incubated for 2 hours at 4°C. The extracts were centrifuged at 16,000*g* at 4°C for 15 minutes, and the resultant supernatant was incubated with prepared antibody beads from the above step. The tube was rotated overnight at 4°C. Then, the agarose gel was washed five times with the NEBT buffer and eluted with elution buffer (0.2 M Glycine, 0.5% Triton X-100 [pH 2.5]). Anti-FER and anti-EBP1 antibody were used for immunoblot assay to detect the immunoprecipitates.

For performing a Co-IP assay using FLAG agarose, *FER-FLAG* protein extract was prepared in a similar manner as described above. The protein extract was incubated with prewashed anti-FLAG M2 agarose gel (A2220, Sigma-Aldrich) overnight at 4°C. Then, the agarose gel was washed five times with the NEBT buffer and eluted with 3 × FLAG peptide (F4799, Sigma-Aldrich). Anti-FLAG (M20008, Abmart) and anti-EBP1 antibody were used for immunoblot assay to detect the immunoprecipitates.

### Phosphorylation and dephosphorylation assays

The ABA-induced phosphorylation coexpression system was established similarly as described previously [49]. Vectors of pACYCDuet-1 (pACYC for short) (71147, Novagen) and pRSFDuet-1 (pRSF for short) (71341, Novagen) were used for this system. For phosphorylation assay, pACYC-PYL1-FER, pACYC-PYL1-FER^K565R^, and pRSF-ABI1-EBP1 were constructed. pRSF-ABI1-EBP1 together with pACYC-PYL1-FER (or pACYC-PYL1-FER^K565R^) were transformed into BL21 *E*. *coli*. The transformed *E*. coli were inoculated into LB medium (containing kanamycin and chloromycetin) and cultured at 37°C until OD_600_ reached 0.6. Then, 500 μM isopropyl-β-d-thiogalactoside (IPTG) was added to induce the protein expression for 2 hours before 50 µM ABA was added into the bacterial culture to release the FER phosphorylation activity for 10 minutes. The dephosphorylation assay was performed as described in the manufacturer’s manual using FastAP Thermosensitive Alkaline Phosphatase (EF0651, Thermo Fisher Scientific, USA). Immunoblot assay was performed to detect the phosphorylation band shift using anti-His antibody. To clearly separate the phosphorylated and dephosphorylated protein, 20% (V/V) glycerol was added into the PAGE gel, and the electrophoresis was performed for about 15 hours under 60 V voltage.

For immunoprecipitation-phosphorylation (IP-phosphorylation) assay, the native EBP1 protein was immunoprecipitated using EBP1 antibody and A/G agarose, as described earlier. Four-week-old Col-0 and *fer-4* plants were soaked in 1/2 MS liquid medium with or without 1 μM RALF1 for 30 minutes. Then, roots were ground to a fine powder in liquid nitrogen for immunoprecipitation assay, as described earlier. FER antibody [12] was used to detect the phosphorylation status of FER. EBP1 antibody was used for analyzing loading control. Antibodies against pSer (ab9332, Abcam) and pThr (9381, Cell Signaling Technology) were used to detect the phosphorylation of EBP1 protein. We adjusted the level of total EBP1 protein to be the same among different plant samples so that the changes in phosphorylation levels of EBP1 can be easily visible.

### EBP1 phosphorylation sites identification using mass spectrometry

SDS-PAGE was performed, and the gel was stained by Coomassie G-250, as described [66]. The interest bands were isolated and placed into tubes. Mass spectrometry was performed as described by Du and colleagues [23]. The band strips were first managed by destaining and dehydration. Then, after reducing and alkylating, protein was enzymolyzed by trypsase (V511A Promega). Then, mass spectrometry was performed using Orbitrap, followed by LTQ. Raw data were analyzed by Xcalibur v.2.1 (Thermo Scientific, Waltham, MA, USA) and Proteome Discoverer v.1.3 beta (Thermo Scientific, Waltham, MA, USA) against the *Arabidopsis* database (in early 2016.10.1, found in UniProt/Swiss-Prot and UniProt/TrEMBL).

### Phylogenetic tree construction

Using an aa sequence of *Arabidopsis* EBP1, genome sequences of different species were searched with the BLAST tool in the National Center for Biotechnology Information (NCBI). Sequences of EBP1-like proteins from different species were downloaded from NCBI and aligned using ClustalX2.1 and Bioedit with default settings. A phylogenetic tree was built with MEGA5 software using the Neighbor-Joining method with the following parameters: Kimura 2-parameter; Bootstrap replications 1000; Random seed 64238. The iTOL [67] software was used to display the phylogenetic tree.

### GUS staining assay

Plant materials of indicated growth stages were collected and incubated in GUS staining solution (50 mM sodium phosphate [pH 7.2], 0.1% Triton X-100, 2 mM potassium ferrocyanide, 2 mM potassium ferricyanide, 10 mM EDTA, 1 mM X-Gluc). The incubated samples were infiltrated under vacuum for 10 minutes. The vacuum was released slowly. Then, the samples were incubated in the GUS staining solution in the dark for 1 hour at 37°C. After removing the GUS staining solution, the materials were incubated in 70% ethanol at room temperature for about 6 hours (the 70% ethanol was renewed several times when the solution turned chromatic). Then, the photos were taken under a dissecting microscope.

### RALF1 treatment

Seedlings were treated with RALF1 in liquid medium. To avoid manipulation-related effect during plant transfer from solid to liquid medium, we transferred the seedlings into liquid 1/2 MS medium and preincubated them for 12 hours before RALF1 treatment. Then, the RALF1 peptide (or same volume of buffer without RALF1 as mock control) was added to the medium for the indicated time period.

Root growth inhibition in response to RALF1 was recorded as described [20,23], with some modifications. Seeds were germinated on 1/2 MS agar plates vertically positioned for 5 days under constant light at 23°C. Then, seedlings were aseptically transferred to 500 μL liquid medium containing 1/2 MS and 1 μM RALF1 peptides in a 24-well cluster plate (3524, Costar). Then, the seedlings were incubated at 23°C for 48 hours with mild shaking (100 rpm) before measuring.

For RALF1-induced mRNA and protein accumulation assay, 7-DAG seedlings were collected and soaked in 1/2 MS liquid medium with or without 1 µM RALF1 for 2 hours or as otherwise indicated for time course. Then, the seedlings were collected for mRNA or protein extraction. Total RNA was extracted using RNAiso Plus (9109, Takara) with the method described in the manufacturer’s manual. The first-strand cDNA was synthesized using a Takara PrimeScript RT reagent Kit with gDNA Eraser (RR047A, Takara).

For total protein extraction, the collected seedlings were ground to a fine powder in liquid nitrogen and then resuspended and extracted in RIPA buffer (50 mM Tris, 150 mM NaCl, 1% NP-40, 0.5% Sodium deoxycholate, 0.1% SDS, 1 mM PMSF, 1 × protease inhibitor cocktail, 1 × phosphatase inhibitor [pH 8.0]) for 1 hour.

### In vivo CRD, MG132, and CHX treatment assay

Generally, to avoid manipulation-related effect during plant transfer from solid to liquid medium, we transferred the seedlings into liquid 1/2 MS medium and preincubated them for 12 hours before treatment.

The procedure for CRD treatment was as described [68]. The 7-DAG seedlings were collected and placed in incubation buffer (1 mM pipes [pH 6.25], 1 mM sodium citrate, 1 mM KCl, 15 mM sucrose), 200 μM CRD (C3394, Sigma-Aldrich), with or without 1 µM RALF1. The samples were infiltrated under vacuum for 3 minutes in incubation buffer, followed by incubation in 23°C for indicated times. Then, plant samples were harvested for RNA extraction.

Treatment of plants with MG132 and CHX was conducted as described [69]. Seven-day-old Col-0 and *fer-4* seedlings grown on 1/2 MS agar plates were collected and transferred to liquid MS medium (with or without 1 µM RALF1) in the presence or absence of 50 μM MG132 (S2619, Selleckchem) or 100 μM CHX (01810, Sigma-Aldrich) for 2 hours. Whole-plant samples were harvested and frozen in liquid nitrogen for protein extraction and immunoblot assay.

### Polysome profiling

The method of polysome profiling was as described [70]. To avoid manipulation-related effect during plant transfer from solid to liquid medium, we transferred the seedlings into liquid 1/2 MS medium and preincubated them for 12 hours. Then, 7-DAG seedlings were treated with or without 1 µM RALF1 for 30 minutes and ground in liquid nitrogen, followed by resuspension in polysome extraction buffer. The extract was loaded onto a 15%–60% sucrose gradient and spun in a Beckman Optima L-100XP centrifuge at 30,000 rpm for 4 hours at 4°C. Sixteen fractions were collected by a gradient fractionator for RNA extraction and reverse transcription. The 40S and 80S of ribosome and polysomes were quantified by OD 260 absorbance profile. RNA was extracted using RNAiso Plus (9109, Takara) with the method described in the manufacturer’s manual. The first-strand cDNA was synthesized using a Takara PrimeScript RT reagent Kit with gDNA Eraser (RR047A, Takara). *Actin* was used as reference gene.

### Immune-fluorescence labeling

The procedure was as described [71], with some modifications. Seven-DAG plant tissues (with or without 1 µM RALF1 treatment for 2 hours) were fixed by paraformaldehyde-based fixative. Fixed plant tissues were incubated with anti-EBP1 antibody at 4 µg/mL overnight at 4°C. The labeled tissues were probed with an IF555 Goat Anti-Mouse IgG (GM200G-37C, Sungene) secondary antibody for confocal observation. DAPI (D9542, Sigma-Aldrich) was used for nucleus staining. The excitation wavelengths for imaging IF555 and DAPI were 561 and 405 nm, respectively.

### Nucleus fractionation

Briefly, 4-week-old rosettes of Col-0, *fer-4* were collected for nucleus fractionation. Rosettes (with or without 1 µM RALF1 treatment for 2 hours) were ground to a fine powder in liquid nitrogen and resuspended in 100 μL fractionation buffer as described [72]. The suspension mixture was centrifuged at 3,000*g* for 10 minutes, and the supernatant was used as cytosolic fraction. The nuclei-containing pellets were resuspended in 500 μL fractionation buffer and centrifuged at 3,000*g* for 5 minutes to wash out the cytosolic residues in the supernatant. This step was repeated five times to obtain the nuclei-containing pellet fraction. The nuclear fractions were resuspended with 100 μL RIPA buffer (containing 1 mM PMSF, 1 × protease inhibitor cocktail, 1 × phosphatase inhibitor) on ice for 1 hour for nucleus protein extraction. Then, aliquots of the cytosolic fraction and nucleus fraction were boiled in SDS-PAGE sample buffer for immunoblot assay.

### Fluorescence microscopy

Subcellular localization of fusion proteins in transgenic *Arabidopsis* was performed in roots from 7-DAG seedlings grown in a vertical position on 1/2 MS medium supplemented with 0.8% sucrose and solidified with 1% (w/v) agar. RALF1 treatment was performed by transferring seedlings to liquid 1/2 MS medium containing 1 μM RALF1 peptide in 23°C for 2 hours. The excitation lines for imaging Hoechst 33258 and GFP were 405 and 488 nm, respectively. Five-DAG etiolated seedlings were collected and immersed into PI staining solution (0.01 M PBS [pH 7.4], 0.01 μM PI) for 2 hours. The excitation lines for imaging GFP, chlorophyll red auto fluorescence, and PI staining were 488, 561, and 488 nm, respectively. The quantification of fluorescent protein signal is performed as described [73].

### ROS burst assay

The method of ROS burst measurement assay was as described [16]. Four leaf discs (4 mm in diameter) per individual genotype were collected into 96-well plates containing sterile water and recovered overnight. The next day, the sterile water was replaced by a solution containing 20 μm/L luminol L-012 (Sigma-Aldrich), 1 μg/mL horseradish peroxidase (HRP, Sigma-Aldrich), and 100 nm/L flg22, 1 μM RALF1 (or without RALF1 in control). Luminescence was measured for the indicated time period using Fluoroskan Ascent FL (Thermo Scientific).

### Proton secretion assay

The proton secretion assay was performed as described [20]. Seedlings were vertically grown for 5 days on 1/2 MS solid plate. To avoid manipulation-related effect during plant transfer from solid to liquid medium, we transferred the seedlings into liquid 1/4 MS medium and preincubated them for 12 hours before reaction. Then, different genotype lines were transferred to a microwell containing 1 μM RALF1, 200 μL 1/4 MS, 1% sucrose (pH 5.8) and a 30 μg/mL pH indicator fluorescein-Dextran conjugate (Sigma-Aldrich). Reaction was terminated at the time points shown in S14E Fig by removing seedlings from wells, and fluorescent intensity (excitation at 485 nm wavelength, emission at 535 nm wavelength) was recorded with Fluoroskan Ascent FL (Thermo Scientific). A standard curve for pH was obtained for each time point and calculated using the 1/4 MS adjusted to pH 5.6, 5.8, and 6.2.

### RNA-seq analysis

This procedure was performed by OE Biotech (Shanghai, People’s Republic of China). Total RNA was extracted using mirVana miRNA Isolation Kit (Ambion) following the manufacturer protocol. RNA integrity was evaluated using the Agilent 2100 Bioanalyzer (Agilent Technologies, Santa Clara, CA, USA). The samples with RNA integrity number (RIN) ≥ 7 were subjected to the subsequent analysis. The libraries were constructed using TruSeq Stranded mRNA LTSample Prep Kit (Illumina, San Diego, CA, USA) according to the manufacturer instructions. The libraries were sequenced on the Illumina sequencing platform (HiSeqTM 2500 or Illumina HiSeq X Ten), and 125 bp/150 bp paired-end reads were generated. Raw data (raw reads) were processed using NGS QC Toolkit [74]. The reads containing poly-N and the low-quality reads were removed to obtain the clean reads. Then, the clean reads were mapped to reference genome using HISAT2 [75]. The FPKM value of each gene was calculated using cufflinks, and the read counts of each gene were obtained by htseq-count [76]. Differentially expressed genes were identified using the DESeq [77] R package functions estimateSizeFactors and nbinomTest. *P* value < 0.05 and foldChange > 2 or foldChange < 0.5 were set as the threshold for significantly differential expression. Genes with more than 2-fold change and *P* value < 0.05 were defined as significantly regulated genes. DAVID Functional Annotation tools [53] was used to analyze the functional annotations (*P* value < 0.001) of differentially expressed genes.

The raw RNA-seq data were uploaded to the NCBI database with the access number SRP151541.

### ChIP assay

The ChIP assay was performed as described [78]. To avoid manipulation-related effect during plant transfer from solid to liquid medium, we transferred the seedlings into liquid 1/2 MS medium and preincubated them for 12 hours. Then, seedlings were soaked with 1 μM RALF1 (included in the 1/2 MS liquid medium) or mock control (1/2 MS medium containing protein elution buffer, which is used for RALF1 purification) for 2 hours and then treated with 1% formaldehyde under vacuum for 15 minutes at room temperature. Glycine was added to a final concentration of 0.125 M to stop cross-linking. The seedlings were washed twice with sterile water, frozen in liquid nitrogen, ground to a fine powder, and homogenized in the nuclear extraction buffer 1 (10 mM Tris-HCL [pH 8.0], 0.4 M sucrose, 10 mM MgCl_2_, 0.1 mM PMSF, and protease inhibitor [78430, Thermo Fisher Scientific]). Nuclei were precipitated by centrifugation in a centrifuge at 4,000*g* for 20 minutes, washed with the nuclear extraction buffer 2 (10 mM Tris-HCl [pH 8.0], 0.25 M sucrose, 10 mM MgCl_2_, 1% Triton X-100, 0.1 mM PMSF, and protease inhibitor [78430, Thermo Fisher Scientific]), and lysed in the nuclei lysis buffer (50 mM Tris-HCl [pH 8.0], 10 mM EDTA, 1% SDS, 0.1 mM PMSF, and protease inhibitor [78430, Thermo Fisher Scientific]). Chromatins were sheared by sonication to approximately 500 bp. The chromatin solution was diluted 10-fold with ChIP dilution buffer (16.7 mM Tris-HCl [pH 8.0], 167 mM NaCl, 1.1% Triton X-100, 1.2 mM EDTA, 0.1 mM PMSF, and protease inhibitor [78430, Thermo Fisher Scientific]). Anti-EBP1 antibody (or preimmune serum as negative control) prebound to the A/G agarose (20421, Thermo Fisher Scientific) was mixed with the chromatin solution and incubated at 4°C overnight. Immunocomplexes were precipitated and washed with four different buffers: low-salt buffer (20 mM Tris-HCl [pH 8.0], 150 mM NaCl, 0.2% SDS, 0.5% Triton X-100, 2 mM EDTA), high-salt buffer (20 mM Tris-HCl [pH 8.0], 500 mM NaCl, 0.2% SDS, 0.5% Triton X-100, 2 mM EDTA), LiCl washing buffer (20 mM Tris-HCl [pH 8.0], 0.25 M LiCl, 1% NP40, 1% sodium deoxycholate, 1 mM EDTA), and TE washing buffer (10 mM Tris-HCl [pH 8.0], 1 mM EDTA). The bound chromatin fragments were eluted with the elution buffer (50 mM Tris-HCl [pH 8.0], 10 mM EDTA, 1% SDS), and the cross-links were reversed by incubating at 65°C overnight. The mixture was treated with protease-K for 1 hour at 45°C to remove proteins. DNA was extracted by phenol/chloroform/isoamyl alcohol and precipitated with 2-fold volume of 100% ethanol at −80°C for 4 hours. To recover the DNA, it was spun at 16,000 rpm for 20 minutes at 4°C. The pellet was dried briefly and resuspended in 25 μL TE buffer for further real-time PCR analysis.

We have screened promoters of the following 17 genes: *CML38*, AT1G76650; *CML40*, AT3G01830; *CKX4*, AT4G29740; *ERF1B*, AT3G23240; *ERF2*, AT5G47220; *ERF098*, AT3G23230; *SAUR9*, AT4G36110; *SAUR41*, AT1G16510; *RVE1*, AT5G17300; *RVE2*, AT5G37260; *RVE3*, AT1G01520; *WAK2*, AT1G21270; *GA2OX1*, AT1G78440; *ANAC036*, AT2G17040; *SIGE*, AT5G24120; *IPS1*, AT3G09922; *EXPA16*, AT3G55500.

### EMSA

The EMSA was performed as described [78], with some modified steps. EBP1-GST protein and GST protein were used for EMSA assay. The primer sequences were synthesized and labeled with FITC fluorescence probe (TsingKe Biological Technology). The DNA-EBP1 binding reaction contained 100 pg probe, 100 ng EBP1-GST protein, 10 mM Tris (pH 7.5), 5% glycerol, 1 mM MgCl_2_, 50 mM KCl, 0.2 mg/mL bovine serum albumin (BSA), 0.5 mM DTT, 0.5 mg/mL polyglutamate, and the indicated amount of unlabeled competitor. The reactions were incubated at room temperature for 20 minutes and fractioned by electrophoresis in a 6% native polyacrylaminde gel (acrylaminde:bisacrylamide, 29:1) containing 10% glycerol, 89 mM Tris (pH 8.0), 89 mM boric acid, and 2 mM EDTA. The FITC signal was detected after electrophoresis using KODAK 4000MM Image Station.

### Dual-LUC

The Dual-LUC was performed as described [78], with modified steps. The putative EBP1-bound sequence of *CML38* promoter was cloned and constructed into pGreen-0800-LUC vector as the reporter plasmid (*proCML38*::LUC). The effector plasmid *35S*::*EBP1* was constructed using pBI121 vector as described above. The reporter plasmid and effector plasmid were transferred into *Arabidopsis* protoplast simultaneously as described above in BiFC assay. Samples were collected for the Dual-LUC using Dual Luciferase Reporter Gene Assay Kit (RG027, Beyotime). The LUC and REN signals were detected using Modulus Microplate Multimode Reader (Turner Biosystem). Three biological repeats were measured for each sample, and similar results were obtained.

For RALF1-treatment, 0.1 μM RALF1 was added into the transfected protoplasts. Then, the cells were incubated in the dark at 23°C for 16 hours for further experimentation.

### In vitro protein degradation assay

The in vitro protein degradation assay was performed as described [58]. Leaves from 4-week-old *Arabidopsis* were ground in liquid N_2_ and resuspended in the proteolysis buffer (20 mM Tris [pH 7.5], 10 mM MgCl_2_, 10 mM NaCl, 10 mM ATP, and 5 mM DTT). After centrifugation, the supernatants from *Arabidopsis* were mixed with EBP1-His protein (S2C Fig) and incubated at room temperature for 30 minutes. The reactions were stopped by boiling in SDS-PAGE sample buffer. Immunoblot assay was performed to detect the protein. Anti-His (M20001, Abmart) or anti-Actin (M20009, Abmart) were used to detect EBP1-His or Actin, respectively.

### Statistical analyses

Statistical significance was determined based on one-way ANOVA analysis using SPSS 23.0 software (SPSS, USA).

## Supporting information

S1 Fig Y2H analysis of interaction between EBP1 and multiple *Cr*RLK1L subfamily members. SD/-Ade/-Leu/-His selection medium containing 20 mM 3-AT was used for screening yeast growth. EBP1 was cloned into the AD vector. *Cr*RLK1L subfamily members were cloned into the BD vector. All assays were performed in four independent experiments, and similar results were obtained. AD, active domain; BD, binding domain; *Cr*RLK1L, *Cr*RLK1L, *C*. *roseus* receptor-like kinase 1-like kinase; EBP1, ErbB3, binding protein 1; Y2H, yeast two-hybrid.
(DOCX)

S2 Fig Purification of EBP1 protein, protein expression in the BiFC assay, and EBP1 antibody analysis. (A) Purification of EBP1-GST. The band of EBP1-GST is indicated by black triangles. M: marker; 1: before induction; 2: after IPTG induction; 3: duplication of lane 2; P: purified protein. (B) Protein expression in the BiFC assay. Mesophyll protoplasts in the BiFC assay were collected, and total protein extract was used for SDS-PAGE–western blot analysis. The proteins expressed in the BiFC assay were detected by GFP antibody. FER-nVenus, HERK2-nVenus, and EBP1-cCFP proteins are indicated. (C) Purification of EBP1-His protein (used for EBP1 antibody production). The band of EBP1-His is indicated by black triangles. M: marker; P: purified protein. (D, E) The specificity of EBP1-antibody was tested by a western blot using protein extracts from Col-0, *EBP1-GFP* (D), and *EBP1-FLAG* (E) plants. Anti-EBP1, anti-GFP, and anti-FLAG antibodies were used for immunoblot assay. All assays were performed in three independent experiments, and similar results were obtained. BiFC, bimolecular fluorescence complementation; cCFP, C-terminal cyan fluorescent protein; EBP1, ErbB3-binding protein 1; FER, FERONIA; GFP, green fluorescent protein; GST, glutathione S-transferase; IPTG, isopropyl-β-d-thiogalactoside.
(DOCX)

S3 Fig Co-IP analysis of EBP1 and FER. Immunoblot assay was performed using FLAG agarose. FLAG antibody and EBP1 antibody were used to detected FER-FLAG and EBP1, respectively. The phosphorylated FER-FLAG and dephosphorylated FER-FLAG are indicated. Three independent experiments were performed, and similar results were obtained. Co-IP, coimmunoprecipitation; EBP1, ErbB3-binding protein 1; FER, FERONIA.
(DOCX)

S4 Fig Phylogenetic analysis of EBP1. Phylogenetic analysis of EBP1 in diverse species. EBP1 homologs in *Arabidopsis* and *Homo sapiens* are indicated. Kingdoms of Animalia, Plantae, Fungi, and Protista are highlighted by a red, green, yellow, and purple background, respectively. The EBP1 homologs in the Plantae kingdom are zoomed in on the right of the sketch, and *At*EBP1 is marked by a red arrow. EBP1, ErbB3-binding protein 1.
(DOCX)

S5 Fig Sequence alignment and conserved motifs and domains of EBP1. EBP1 homologs in *Arabidopsis*, Rice, *Zea mays*, *Glycine*, *Brassica*, *Chlamydomonas*, *Physcomitrella*, and Human were analyzed with the ClustalX and then edited with BioEdit. The secondary structure of Human EBP1 is assigned along the sequence. α-helixes, β-strands, and turns are indicated. Identical residues are highlighted by a red background. Conserved residues are highlighted by red font. The identified *At*EBP1 phosphorylation sites regulated by FER are marked with red diamonds. NLS and NLRs are indicated. Residue numbers are shown on the left of the sequence. EBP1, ErbB3-binding protein 1; FER, FERONIA; NLR, nuclear localization–related region; NLS, nucleus-localization sequence.
(DOCX)

S6 Fig The mRNA expression pattern of *EBP1*. (A-J) The expression patterns of *EBP1*::GUS reporter in different tissues and organs. (A, B) The expression of GUS reporter in 7-DAG nontransgenic Col-0 (A) and *proEBP1*::GUS transgenic plant (B). (C, D) The expression of GUS reporter in 2-DAG seedlings of Col-0 (C) and *proEBP1*::GUS transgenic plant (D). (E, F) The expression of GUS reporter in cotyledons of 7-DAG Col-0 (E) and *proEBP1*::GUS transgenic plant (F). (G, H) The expression of GUS reporter in 7-DAG root tip of Col-0 (G) and *proEBP1*::GUS transgenic plant (H). (I, J) The expression of GUS reporter of 4-week-old rosettes of Col-0 (I) and *proEBP1*::GUS (J). Three independent experiments were performed, and similar results were obtained. (K) Expression profiles of EBP1. The data were collected from Plant eFP at http://bar.utoronto.ca/eplant/. Signal threshold was set to 50%. DAG, day after germination; EBP1, ErbB3-binding protein 1; GUS, β-glucuronidase.
(DOCX)

S7 Fig *EBP1* mRNA content assay, EBP1 protein accumulation in nucleus, and identification of *EBP1-GFP*. (A) *EBP1* mRNA levels in response to RALF1 peptide in WT. *ACTIN* was used as reference gene. Data points are means +/− SD. (B) *EBP1* mRNA decay in response to 1 µM RALF1 peptide. qRT-PCR assay was performed to detect the time courses (0, 1, 2 hours) of relative gene expression level of *EBP1* with CRD treatment. *ATHSPRO2* was used as positive control. *EIF4A1* was used as reference gene. Data points are means +/− SD. Similar results of (A) and (B) were obtained in three independent experiments. (C) Immunoblot analyses of EBP1 in both nuclear and nuclei-depleted soluble fractions from Col-0 treated for 2 hours respectively, with 1 µM PEP1 peptide, 1 µM ABA, 50 nM NAA, and 1 µM RALF1 peptide. Antibody against Histone H3 was used to mark nucleus fraction. Antibody against GAPC was used to mark cytosolic fraction. Data shown are representative of three independent experiments with similar results. (D) PCR identification of *35S*::*EBP1-GFP* and *35S*::*mEBP1-10A-GFP* (*EBP1-GFP* or *mEBP1-10A-GFP* for short) plants was performed using genomic DNA extracted from each plant lines. Primers of EBP1 paired with GFP tag were used for detecting *EBP1-GFP* (or *mEBP1-10A-GFP*) construction. (E) Real-time RT-PCR analysis of *EBP1* mRNA levels in the Col-0, *EBP1-GFP*, and *mEBP1-10A-GFP* plants. *ACTIN* was used as reference gene. Data represent means +/− SD. (F) Immunoblot analyses of EBP1-GFP protein in 7-DAG *EBP1-GFP* and *mEBP1-10A-GFP* transgenic plants (with or without 1 μM RALF1 treatment for 2 hours) using EBP1 antibody. Actin is shown in lower panel to indicate loading control. Data shown are representative of three independent experiments with similar results. (G) EBP1-GFP was detected by GFP fluorescence in the guard cell from 4-week-old *EBP1-GFP* leaves, Bar = 25 μm. Values with different letters are significantly different (*P* < 0.05) from each other, tested by one-way ANOVA. Numerical data used to generate the plot in A, B, and E are provided in S1 Data. ABA, abscisic acid; CRD, cordycepin; DAG, day after germination; EBP1, ErbB3-binding protein 1; GAPC, cytosolic glycolytic GAPDHs; GFP, green fluorescent protein; NAA, 1-naphthaleneacetic acid; qRT-PCR, quantitative reverse transcription PCR; RALF1, rapid alkalinization factor 1; WT, wild type.
(DOCX)

S8 Fig EBP1 protein distribution in response to RALF1. (A) The fluorescence of nucleus-accumulated EBP1-GFP merged with nucleus indicator signal. Nucleus was stained and indicated by Hoechst 33258 nucleus dye. The plant was treated with or without 1 μM RALF1 for 2 hours. (B) Fluorescence distribution of *35S*::GFP in root with or without 1 μM RALF1 treatment for 2 hours. (C) Immune-fluorescent labeling assay. Seven-DAG seedlings (with or without 1 µM RALF1 treatment for 2 hours) were used for immune-fluorescent labeling assay. The signal of EBP1 and DAPI are shown. Preimmune serum (“preim”) was used as negative control. The nucleus fluorescence is indicated by white arrows. Assays of this figure were performed in three independent experiments, and similar results were obtained. DAG, day after germination; EBP1, ErbB3-binding protein 1; RALF1, rapid alkalinization factor 1.
(DOCX)

S9 Fig ESI mass spectrometric spectra analyses of EBP1 phosphorylation sites. (A-J) The identified EBP1 phosphorylation sites of Ser^13^ (A), Thr^15^ (B), Ser^16^ (C), Thr^242^ (D), Thr^243^ (E), Tyr^245^ (F), Ser^378^ (G), Thr^379^ (H), Ser^387^ (I), and Ser^388^ (J). The identified peptide sequences and the phosphorylation site are displayed. y-ion and b-ion are shown upon the sequences. Three independent experiments were performed, and similar results were obtained. EBP1, ErbB3-binding protein 1; ESI, electrospray ionization.
(DOCX)

S10 Fig Subcellular localization of EBP1-GFP and its mutation forms in protoplast. (A) The subcellular localization of EBP1-GFP and mEBP1-10A-GFP before and after 1 μM RALF1 treatment for 1 hour. The nucleus-localized EBP1-GFP is indicated by white arrows. (B) The ratio of nucleus-located EBP1-GFP, mEBP1-N3A-GFP (“N3A”), mEBP1-M3A-GFP (“M3A”), mEBP1-C4A-GFP (“C4A”), and mEBP1-10A-GFP (“10A”) before and after 1 μM RALF1 treatment for 1 hour. *n* > 330. (C) Fluorescence intensity ratio of nucleus/cytoplasm measurements in EBP1-GFP and mEBP1-10A-GFP after RALF1 treatment. *n* = 10. Data of (B) and (C) represent means. Data points are means +/− SD. Values with different letters are significantly different (*P* < 0.05) from each other, tested by one-way ANOVA. At least three independent experiments of (A–C) were performed, and similar results were obtained. (D) The representative EBP1-GFP and mEBP1-10A-GFP cells used for line scan measurement of yielded plot profiles are selected in (A) with white dashed frames. The white lines inside the images (D) show the areas used for line scan measurements that yielded plot profiles shown in (E). The chosen layer for intensity was analyzed using ImageJ. Numerical data used to generate the plot in B, C, and E are provided in S1 Data. C, cytoplasmic signal; EBP1, ErbB3-binding protein 1; GFP, green fluorescent protein; N, nucleus signal.
(DOCX)

S11 Fig Identification of *EBP1* mutant and overexpression lines. (A) PCR analysis of *EBP1* T-DNA insertion lines. (B) The diagram of *EBP1* gene structure. Exon, intron, and UTR are indicated by white frames, black lines, and gray blocks, respectively. The precise sites of the T-DNA insertions are displayed by the framed name of *ebp1* mutant lines. (C) Immunoblot analyses of EBP1 protein in the WT and *ebp1* mutant lines using anti-EBP1. Ponceau S staining is shown as loading control. Anti-EBP1 was used for immunoblot assay. (D) Real-time RT-PCR analysis of *EBP1* mRNA levels in the WT and *EBP1-OE* lines. *ACTIN* was used as reference gene. Data represent means. Data points are means +/− SD. Values with different letters are significantly different (*P* < 0.05) from each other, tested by one-way ANOVA. Numerical data used to generate the plot in D are provided in S1 Data. EBP1, ErbB3-binding protein 1; *EBP1-OE*, *EBP1-overexpression*; RT-PCR, reverse transcription PCR; WT wild type.
(DOCX)

S12 Fig EBP1 regulates hypocotyl growth. Phenotype (A, Bar = 10 mm) and statistical analysis (B, *n* > 10) show that *ebp1* plants have shorter hypocotyls, whereas *EBP1-OE* plants show longer hypocotyls as compared to the WT. (C) Hypocotyl cell size in *fer-4*, Col-0, and *ebp1-1*. Bar = 10 μm. (D) Length of hypocotyl cells from *fer-4*, Col-0, and *ebp1* mutants was measured using ImageJ software. *n* = 12. Data represent means. Data points are means +/− SD. Values with different letters are significantly different (*P* < 0.05) from each other, tested by one-way ANOVA. Data shown in this figure were performed in four biological replicates, and similar results were obtained. Numerical data used to generate the plot in B and D are provided in S1 Data. EBP1, ErbB3-binding protein 1; *EBP1-OE*, *EBP1-overexpression*; WT, wild type.
(DOCX)

S13 Fig Phenotype complement assay. *EBP1-GFP* expressed in *ebp1-1* background rescues the RALF1 sensitivity phenotype in *ebp1-1* mutant. *n* = 25. Data points are means +/− SD. Values with different letters are significantly different from each other, tested by one-way ANOVA. Similar results were obtained in four independent experiments. Numerical data used to generate the plot are provided in S1 Data. EBP1, ErbB3-binding protein 1; RALF1, rapid alkalinization factor 1.
(DOCX)

S14 Fig RALF1 response assays of *ebp1* mutants. (A) flg22-triggered ROS burst in Col-0, *fer-4*, and *ebp1* mutant lines without or with 1 μM RALF1 treatment. Similar results were obtained in three biological replicates. Data points are means +/− SD. Values with different letters are significantly different (*P* < 0.05) from each other, tested by one-way ANOVA. (B) RALF1 activated MAPK cascade immunoblot assay in Col-0, *ebp1* mutant lines. Seedlings with or without 1 μM RALF1-treatment for 2 hours were used for assay. pMAPK antibody (#4370, Cell Signaling Technology) was used to detect pMAPK intensity. Actin was used as loading control. Three independent experiments were performed, and similar results were obtained. (C, D) Proton secretion assay in Col-0, *fer-4*, and *ebp1* mutant lines with or without 1 μM RALF1. Standard curve was analyzed as y = 1.1863x − 26.314, R^2^ = 0.9999. Quantification of proton secretion was performed on three technical replicates. Three independent experiments were performed, and similar results were obtained. Numerical data used to generate the plot in A, C, and D are provided in S1 Data. flg22, 22 amino acid fragment of bacterial flagellin; MAPK, mitogen-activated protein kinase; pMAPK, phoshpo-MAPK; RALF1, rapid alkalinization factor 1; ROS reactive oxygen species.
(DOCX)

S15 Fig Functional annotation of genes affected in RNA-seq analysis. Functional annotation of affected genes in *ebp1-1* mutant versus Col-0 (A), *ebp1-1* versus *fer-4* (B), *ebp1-1* versus *fer-4* versus RALF1-treated Col-0 (C) are shown. *P* value is shown as −log_10_ (*P* value). Numbers of gene in each functional annotation are shown in the brackets beside the annotations. RALF1, rapid alkalinization factor 1; RNA-seq, RNA sequencing.
(DOCX)

S16 Fig EBP1 associates with gene promoters. (A-D) The diagrams of *CML38* (A), *CKX4* (B), *ERF1B* (C), and *SAUR9* (D) gene structures. The black arrows and the frame indicate the promoter and CDS sequences. Promoter fragments (a, b, c, and d) selected for ChIP-qPCR assay are shown with real line or dashed lines. The real line (or dashed line) indicates the fragment associated (or not) with EBP1 in (E-H). (E-H) ChIP-qPCR assay was performed to screen potential promoter fragments associated with EBP1. The selected promoter fragments (a, b, c, and d) of *CML38* (E), *CKX4* (F), *ERF1B* (G), and *SAUR9* (H) are indicated in (A-D). Seven-DAG Col-0 seedlings (with 1 μM RALF1 treatment for 2 hours) were used for ChIP assay. (I, J, K) *CKX4* (I), *ERF1B* (J), and *SAUR9* (K) gene promoters were immune-precipitated by EBP1 protein. ChIP-qPCR results were quantified by normalization of the EBP1-IP signal with the corresponding Input signal (IP/Input). EBP1 antibody was used to immunoprecipitate EBP1 protein. Preimmune serum (“Preim”) was used for negative control. Quantification of IP/Input (E-K) levels was performed on two technical replicates. Data shown in (I–K) are representative of three independent experiments with similar results. Data represent means. Data points are means +/− SD. Values with different letters are significantly different (*P* < 0.05) from each other, tested by one-way ANOVA. Numerical data used to generate the plot in E-K are provided in S1 Data. CDS, coding sequence; ChIP, chromatin immunoprecipitation; DAG, day after germination; EBP1, ErbB3-binding protein 1; qPCR, quantitative PCR; RALF1, rapid alkalinization factor 1.
(DOCX)

S17 Fig *CML38* Dual-LUC. Relative reporter activity (LUC/REN) of indicated genotypes (Col-0 or *ebp1-1*), RALF1-treatment (0.1 μM RALF1) condition, and *proCML38*::LUC (*CML38* for short) and EBP1 protein expression are shown. Quantification of LUC relative to REN levels was performed in three technical replicates. Similar results were obtained in three independent experiments. Data represent means. Data points are means +/− SD. Values with different letters are significantly different (*P* < 0.05) from each other, tested by one-way ANOVA. Numerical data used to generate the plot are provided in S1 Data. Dual-LUC, transient transcription dual-luciferase assay; EBP1, ErbB3, binding protein 1; LUC, luciferase; REN, Renilla luciferase; RALF1, rapid alkalinization factor 1.
(DOCX)

S18 Fig Gene expressions assays. (A) Relative *CML38* gene expression in *ebp1* mutants and *EBP1-GFP* lines. RALF1 treatments (1 μM) were performed for 2 hours. Quantification of *CML38* relative to *Actin* levels was performed. Three independent experiments were performed, and similar results were obtained. Data represent means. Data points are means +/− SD. Values with different letters are significantly different (*P* < 0.05) from each other, tested by one-way ANOVA. (B-D) Gene expression level of *CKX4* (B), *ERF1B* (C), and *SAUR9* (D) in Col-0, *fer-4*, and *ebp1* mutant lines. *ACTIN* was used as reference gene. Similar results were obtained in two independent experiments. Numerical data used to generate the plot are provided in S1 Data. RALF1, rapid alkalinization factor 1.
(DOCX)

S19 Fig EBP1 protein degradation assay in vitro. (A) The EBP1-His protein was incubated with Col-0 or *fer-4* protein extract for 30 minutes, and (B) the EBP1-His or mEBP1-10A-His protein was incubated with Col-0 protein extract for 30 minutes, followed by SDS-PAGE and western analysis using anti-His or anti-Actin antibody. The assay was performed in three biological replicates, and similar results were obtained. EBP1, ErbB3-binding protein 1.
(DOCX)

S1 Data Numerical data for plots shown in Fig 2B, 2G and 2K; Fig 3H–3K; Fig 4B, 4D and 4F; Fig 5A–5D; Fig 6D and 6F; S7A, S7B and S7E Fig; S10B, S10C and S10E Fig; S11D Fig; S12B and S12D Fig; S13 Fig; S14A, S14C and S14D Fig; S16E–S16K Fig; S17 Fig; S18A–S18D Fig.
(XLSX)

S1 Table Primers used in this study. (DOCX)

## Abbreviations

aa: amino acid
ABA: abscisic acid
ABI2: ABA insensitive 2
AD: active domain
AHA2: *Arabidopsis* H^+^-ATPase 2
ANX1: ANXUR 1
AP: alkaline phosphatase
BAK1: brassinosteroid insensitive 1–associated kinase 1
BD: binding domain
BES1: *bri1*-EMS-suppressor 1
BiFC: bimolecular fluorescence complementation
BIN2: BR insensitive 2
BR: brassinolide
BRI1: BR insensitivity 1
BSU1: *bri1*-suppressor 1
BZR1: Brassinazole resistant 1
CBB: Coomassie Brilliant Blue
cCFP: C-terminal cyan fluorescent protein
Cd: cadmium
ChIP: chromatin immunoprecipitation
ChIP-seq: ChIP sequencing
CHX: cycloheximide
C-NLR: C-terminal NLR
Co-IP: coimmunoprecipitation
CRD: cordycepin
*Cr*RLK1L: *Catharanthus roseus* receptor-like kinase 1-like kinase
Cu: copper
CVY1: CURVY 1
DAG: day after germination
DIC: differential interference contrast
Dual-LUC: transient transcription dual-luciferase assay
EBP1: ErbB3-binding protein 1
EBP1-OE: *EBP1-overexpression*
EFR: EF-Tu receptor
EGFR: epidermal growth factor receptor
elf18: 18 amino acid fragment of bacterial elongation factor Tu
EMSA: electrophoretic mobility shift assay
ErbB1: erythroblastic leukemia viral oncogene homolog 1
FER: FERONIA
FER-KD: FER kinase domain
FER-KD-S: FER-KD fused to S-tag
flg22: 22 amino acid fragment of bacterial flagellin
FLR: *FER-like receptor*
FLS2: flagellin-sensing 2
GAPC: cytosolic glycolytic GAPDHs
GEF- ROP/RAC: guanine nucleotide exchange factor–plant RHO-related GTPases
GFP: green fluorescence protein
GST: glutathione S-transferase
GUS: β-glucuronidase
HDAC2: histone deacetylase 2
HERK2: HERCULES2
HRG: heregulin
LIMYB: L10-interacting MYB domain-containing protein
MAPK: mitogen-activated protein kinase
MS: Murashige and Skoog
NAA: 1-naphthaleneacetic acid
N/C: nucleus/cytoplasm
Ni: nickel
NIK: nuclear shuttle protein-interacting receptor-like kinase
NLR: nuclear localization–related region
NLS: nucleus-localization sequence
N-NLR: N-terminal NLR
NRG: neuregulin
Pb: lead
pFER: phosphorylated FER
PI: propidium iodide
PP2C: protein phosphatase 2C
pSer: phosphoserine
pThr: phosphothreonine
PYL1: pyrabactin resistance 1-like 1
qPCR: quantitative PCR
qRT-PCR: quantitative reverse transcription PCR
RALF: rapid alkalinization factor
Rb: retinoblastoma gene
RIPK: RPM1-induced protein kinase
RLK: receptor-like kinase
RNA-seq: RNA sequencing
RNP: ribonucleoprotein
ROS: reactive oxygen species
SnRK2: sucrose nonfermenting 1–related protein kinase 2
TTFL: transcription–translation feedback loop
WT: wild type
Y2H: yeast two-hybrid
Zn: zinc
