# Supplementary figures and images for: EBP1 nuclear accumulation negatively feeds back on FERONIA-mediated RALF1 signaling
Source: PLoS Biol. 2018 Oct 19;16(10):e2006340. doi: 10.1371/journal.pbio.2006340 (PMC6195255; doi:10.1371/journal.pbio.2006340)

**S1 Fig**

**
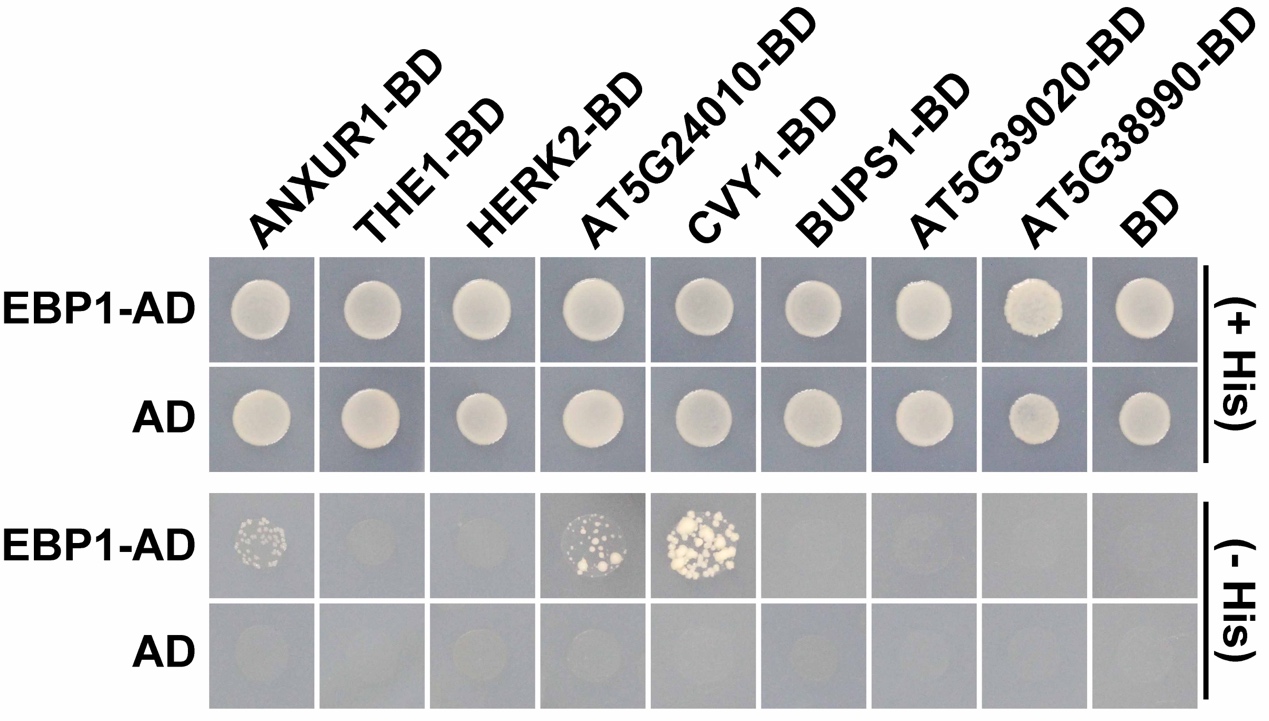
**

Supplement: S1 Fig — SD/-Ade/-Leu/-His selection medium containing 20 mM 3-AT was used for screening yeast growth. EBP1 was cloned into the AD vector. CrRLK1L subfamily members were cloned into the BD vector. All assays were performed in four independent experiments, and similar results were obtained. AD, active domain; BD, binding domain; CrRLK1L, CrRLK1L, C. roseus receptor-like kinase 1-like kinase; EBP1, ErbB3, binding protein 1; Y2H, yeast two-hybrid. (DOCX) [file pbio.2006340.s001.docx]

**S2 Fig**

**
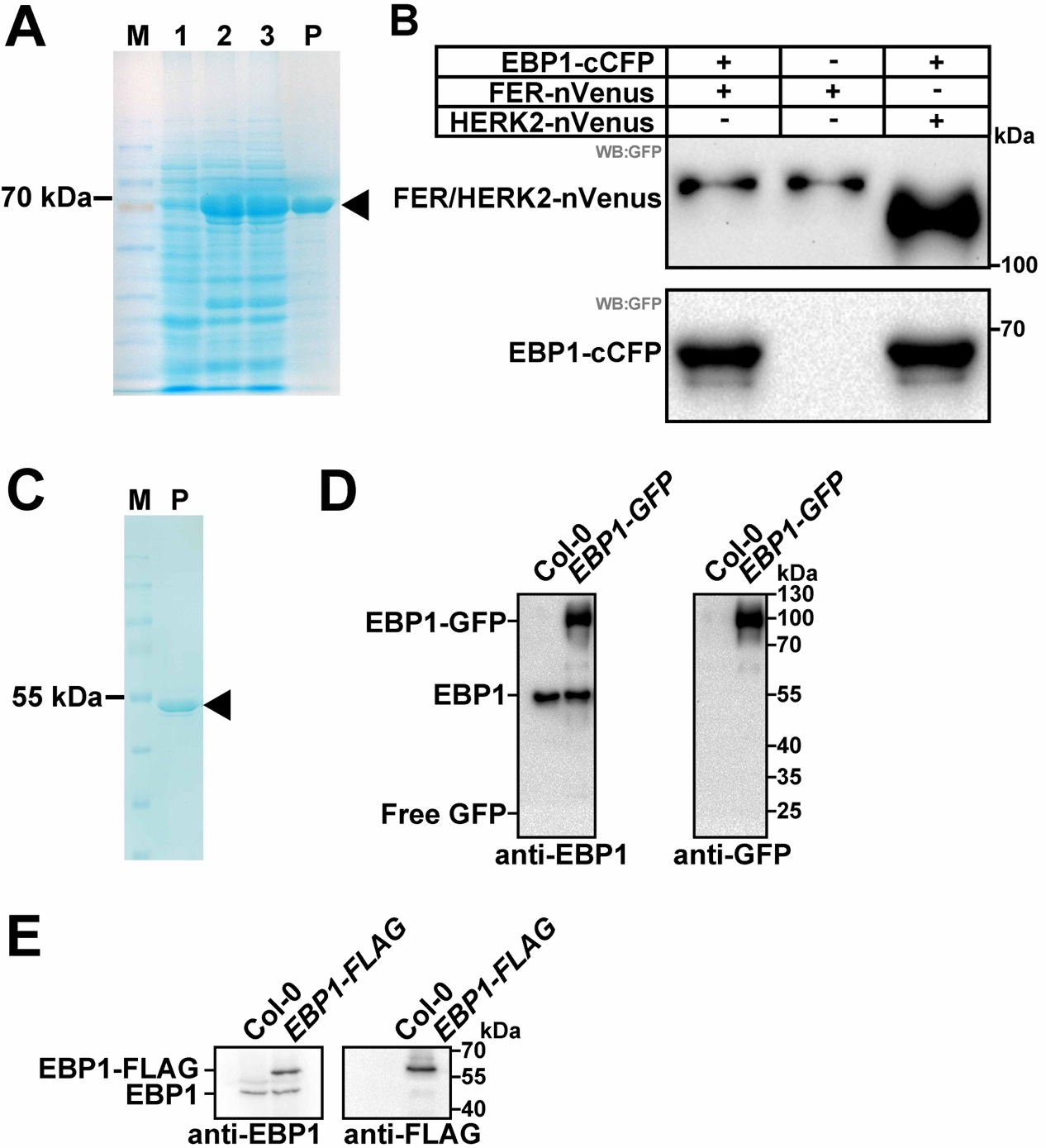
**

Supplement: S2 Fig — (A) Purification of EBP1-GST. The band of EBP1-GST is indicated by black triangles. M: marker; 1: before induction; 2: after IPTG induction; 3: duplication of lane 2; P: purified protein. (B) Protein expression in the BiFC assay. Mesophyll protoplasts in the BiFC assay were collected, and total protein extract was used for SDS-PAGE–western blot analysis. The proteins expressed in the BiFC assay were detected by GFP antibody. FER-nVenus, HERK2-nVenus, and EBP1-cCFP proteins are indicated. (C) Purification of EBP1-His protein (used for EBP1 antibody production). The band of EBP1-His is indicated by black triangles. M: marker; P: purified protein. (D, E) The specificity of EBP1-antibody was tested by a western blot using protein extracts from Col-0, EBP1-GFP (D), and EBP1-FLAG (E) plants. Anti-EBP1, anti-GFP, and anti-FLAG antibodies were used for immunoblot assay. All assays were performed in three independent experiments, and similar results were obtained. BiFC, bimolecular fluorescence complementation; cCFP, C-terminal cyan fluorescent protein; EBP1, ErbB3-binding protein 1; FER, FERONIA; GFP, green fluorescent protein; GST, glutathione S-transferase; IPTG, isopropyl-β-d-thiogalactoside. (DOCX) [file pbio.2006340.s002.docx]

**S3 Fig**


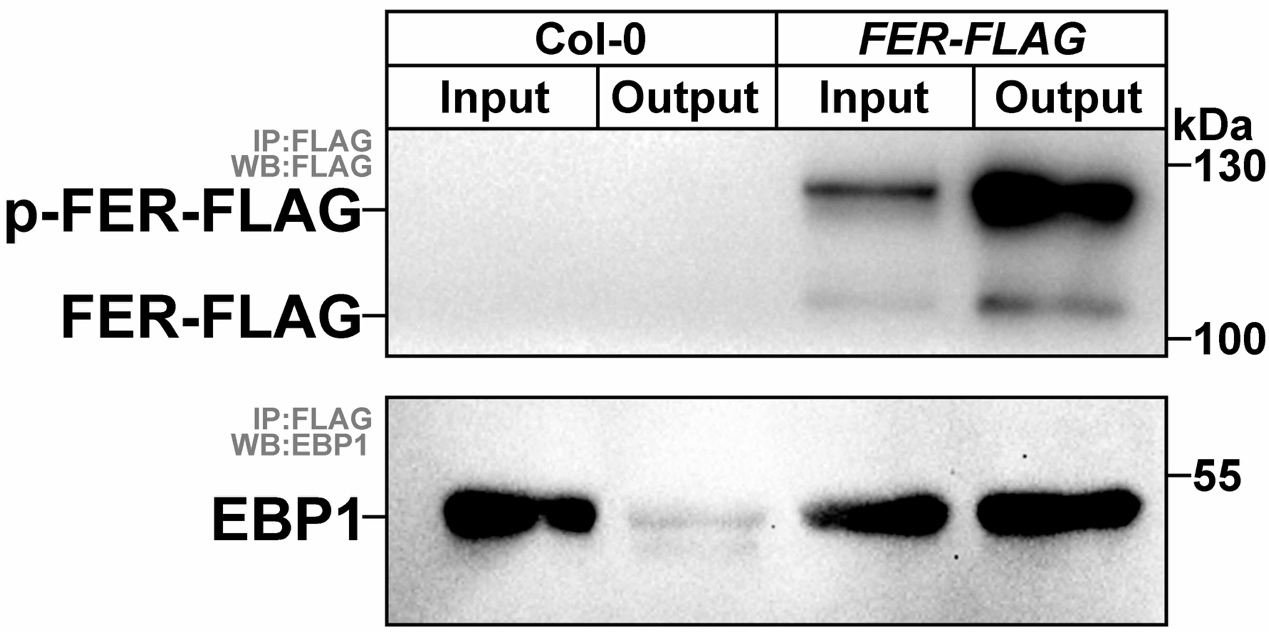

Supplement: S3 Fig — Immunoblot assay was performed using FLAG agarose. FLAG antibody and EBP1 antibody were used to detected FER-FLAG and EBP1, respectively. The phosphorylated FER-FLAG and dephosphorylated FER-FLAG are indicated. Three independent experiments were performed, and similar results were obtained. Co-IP, coimmunoprecipitation; EBP1, ErbB3-binding protein 1; FER, FERONIA. (DOCX) [file pbio.2006340.s003.docx]

**S4 Fig**

**
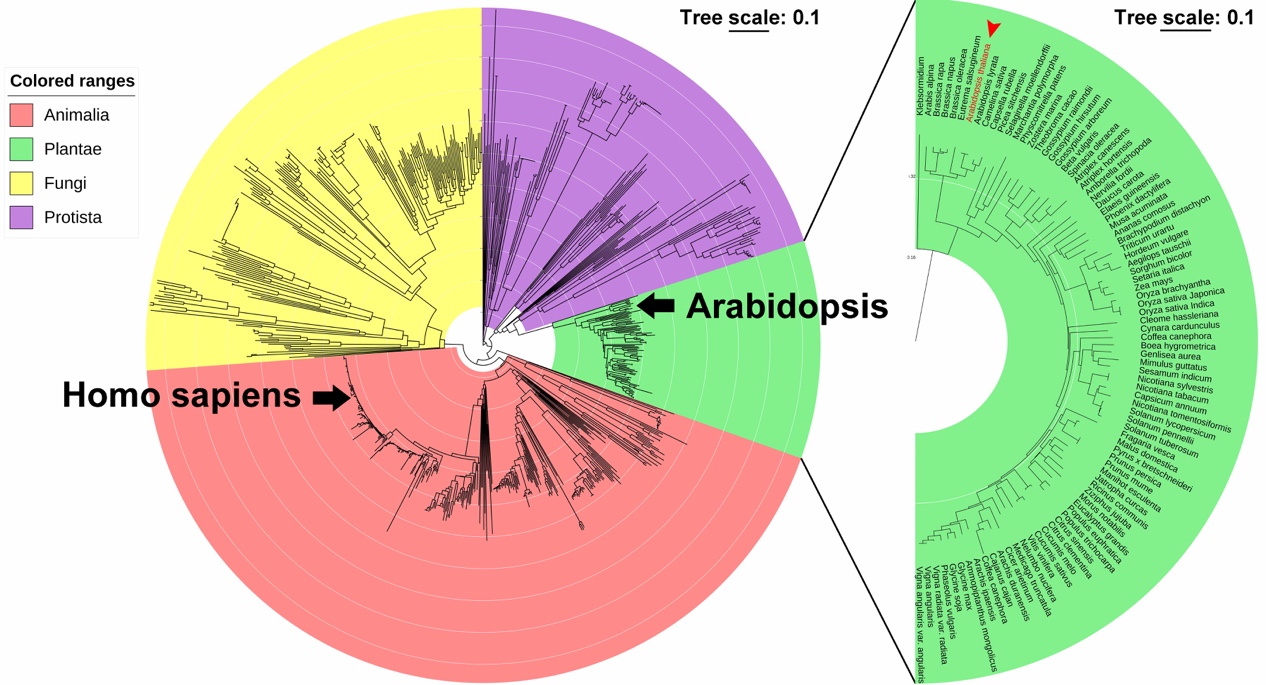
**

Supplement: S4 Fig — Phylogenetic analysis of EBP1 in diverse species. EBP1 homologs in Arabidopsis and Homo sapiens are indicated. Kingdoms of Animalia, Plantae, Fungi, and Protista are highlighted by a red, green, yellow, and purple background, respectively. The EBP1 homologs in the Plantae kingdom are zoomed in on the right of the sketch, and AtEBP1 is marked by a red arrow. EBP1, ErbB3-binding protein 1. (DOCX) [file pbio.2006340.s004.docx]

**S5 Fig**

**
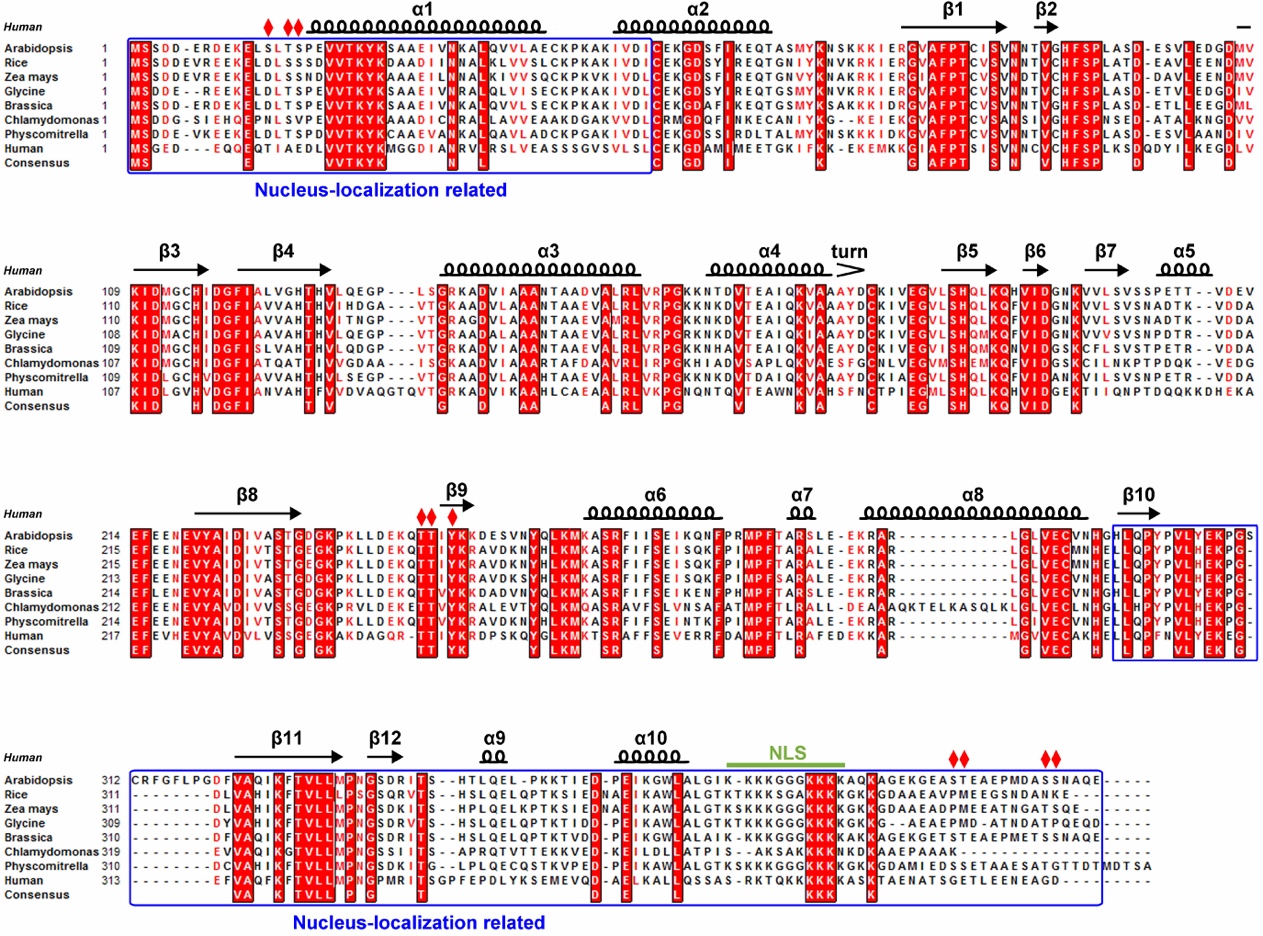
**

Supplement: S5 Fig — EBP1 homologs in Arabidopsis, Rice, Zea mays, Glycine, Brassica, Chlamydomonas, Physcomitrella, and Human were analyzed with the ClustalX and then edited with BioEdit. The secondary structure of Human EBP1 is assigned along the sequence. α-helixes, β-strands, and turns are indicated. Identical residues are highlighted by a red background. Conserved residues are highlighted by red font. The identified AtEBP1 phosphorylation sites regulated by FER are marked with red diamonds. NLS and NLRs are indicated. Residue numbers are shown on the left of the sequence. EBP1, ErbB3-binding protein 1; FER, FERONIA; NLR, nuclear localization–related region; NLS, nucleus-localization sequence. (DOCX) [file pbio.2006340.s005.docx]

**S6 Fig**

**
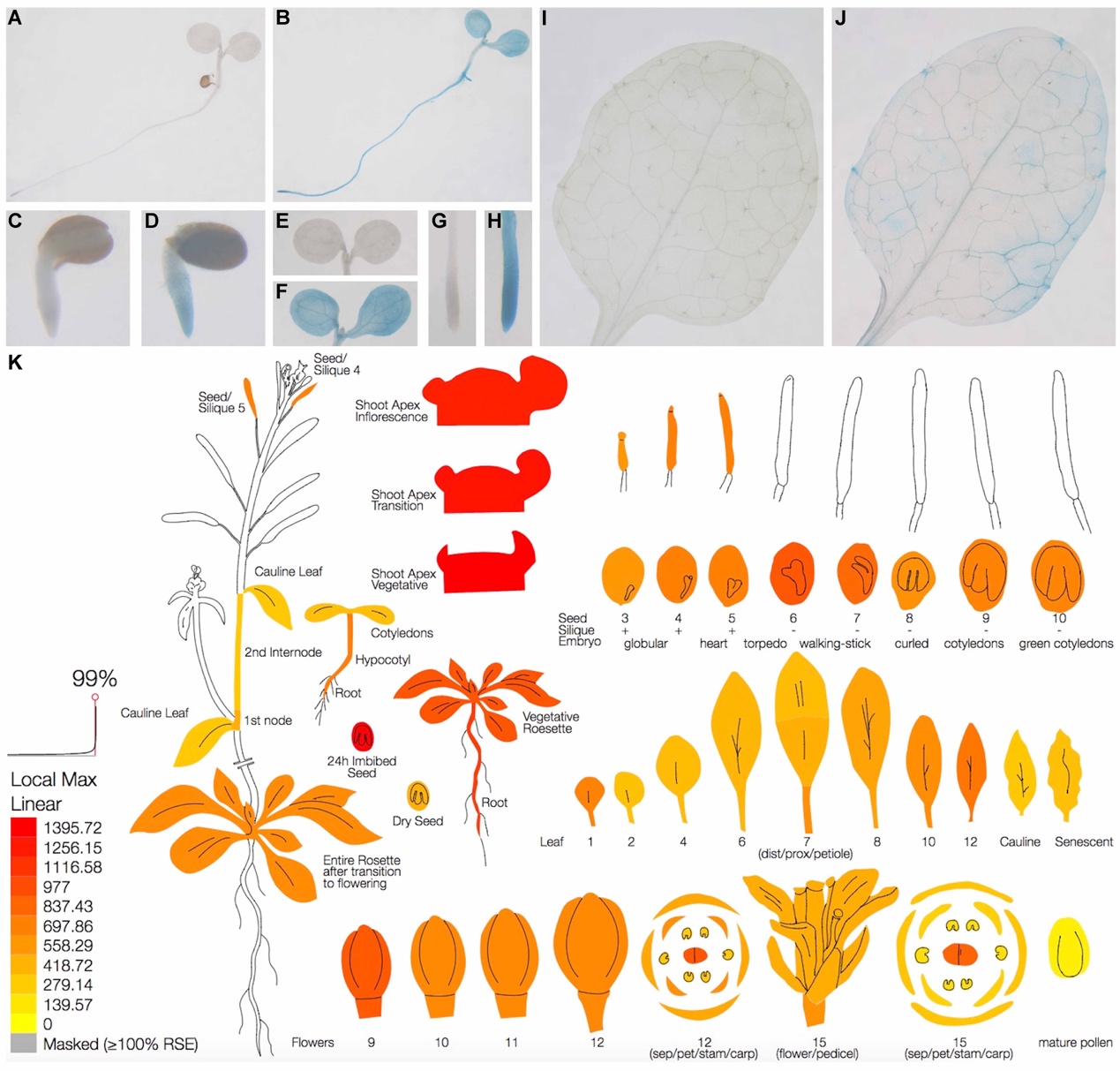
**

Supplement: S6 Fig — (A-J) The expression patterns of EBP1::GUS reporter in different tissues and organs. (A, B) The expression of GUS reporter in 7-DAG nontransgenic Col-0 (A) and proEBP1::GUS transgenic plant (B). (C, D) The expression of GUS reporter in 2-DAG seedlings of Col-0 (C) and proEBP1::GUS transgenic plant (D). (E, F) The expression of GUS reporter in cotyledons of 7-DAG Col-0 (E) and proEBP1::GUS transgenic plant (F). (G, H) The expression of GUS reporter in 7-DAG root tip of Col-0 (G) and proEBP1::GUS transgenic plant (H). (I, J) The expression of GUS reporter of 4-week-old rosettes of Col-0 (I) and proEBP1::GUS (J). Three independent experiments were performed, and similar results were obtained. (K) Expression profiles of EBP1. The data were collected from Plant eFP at http://bar.utoronto.ca/eplant/. Signal threshold was set to 50%. DAG, day after germination; EBP1, ErbB3-binding protein 1; GUS, β-glucuronidase. (DOCX) [file pbio.2006340.s006.docx]

**S7 Fig**

**
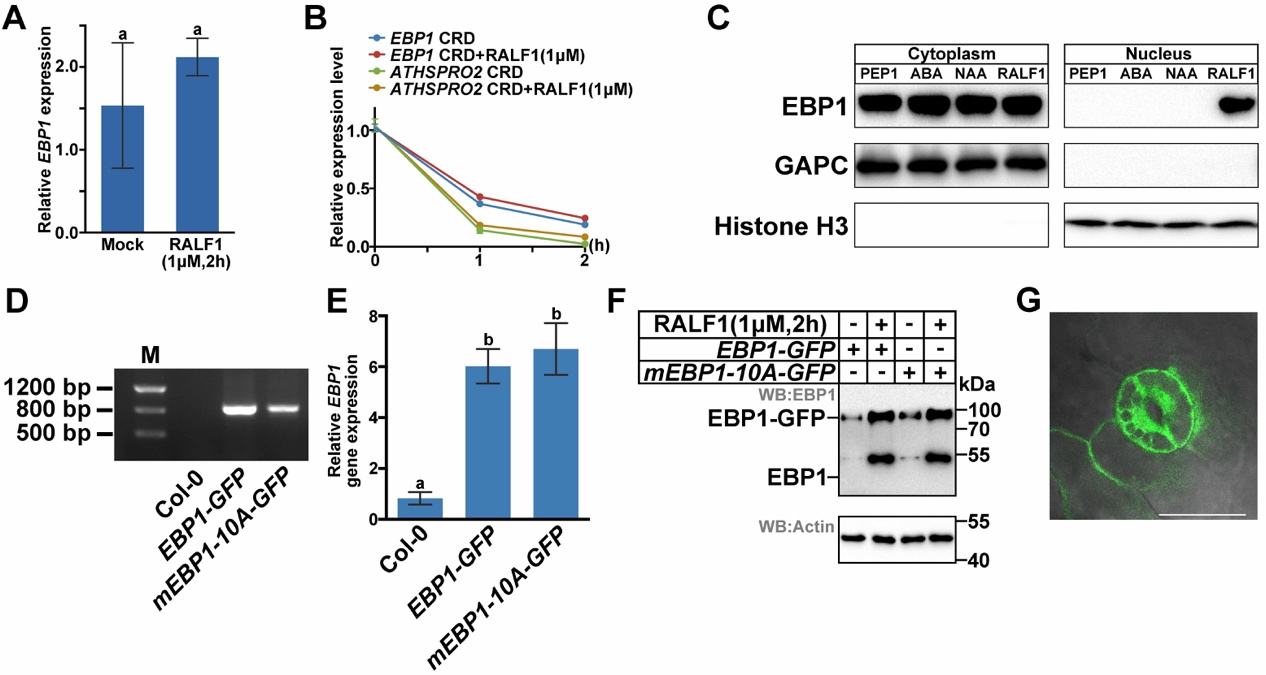
**

Supplement: S7 Fig — (A) EBP1 mRNA levels in response to RALF1 peptide in WT. ACTIN was used as reference gene. Data points are means +/− SD. (B) EBP1 mRNA decay in response to 1 µM RALF1 peptide. qRT-PCR assay was performed to detect the time courses (0, 1, 2 hours) of relative gene expression level of EBP1 with CRD treatment. ATHSPRO2 was used as positive control. EIF4A1 was used as reference gene. Data points are means +/− SD. Similar results of (A) and (B) were obtained in three independent experiments. (C) Immunoblot analyses of EBP1 in both nuclear and nuclei-depleted soluble fractions from Col-0 treated for 2 hours respectively, with 1 µM PEP1 peptide, 1 µM ABA, 50 nM NAA, and 1 µM RALF1 peptide. Antibody against Histone H3 was used to mark nucleus fraction. Antibody against GAPC was used to mark cytosolic fraction. Data shown are representative of three independent experiments with similar results. (D) PCR identification of 35S::EBP1-GFP and 35S::mEBP1-10A-GFP (EBP1-GFP or mEBP1-10A-GFP for short) plants was performed using genomic DNA extracted from each plant lines. Primers of EBP1 paired with GFP tag were used for detecting EBP1-GFP (or mEBP1-10A-GFP) construction. (E) Real-time RT-PCR analysis of EBP1 mRNA levels in the Col-0, EBP1-GFP, and mEBP1-10A-GFP plants. ACTIN was used as reference gene. Data represent means +/− SD. (F) Immunoblot analyses of EBP1-GFP protein in 7-DAG EBP1-GFP and mEBP1-10A-GFP transgenic plants (with or without 1 μM RALF1 treatment for 2 hours) using EBP1 antibody. Actin is shown in lower panel to indicate loading control. Data shown are representative of three independent experiments with similar results. (G) EBP1-GFP was detected by GFP fluorescence in the guard cell from 4-week-old EBP1-GFP leaves, Bar = 25 μm. Values with different letters are significantly different (P < 0.05) from each other, tested by one-way ANOVA. Numerical data used to generate the plot in A, B, and E are provided in S1 Data. ABA, abscisic acid; CRD, cordycepin; DAG, day a [file pbio.2006340.s007.docx]

**S8 Fig**

**
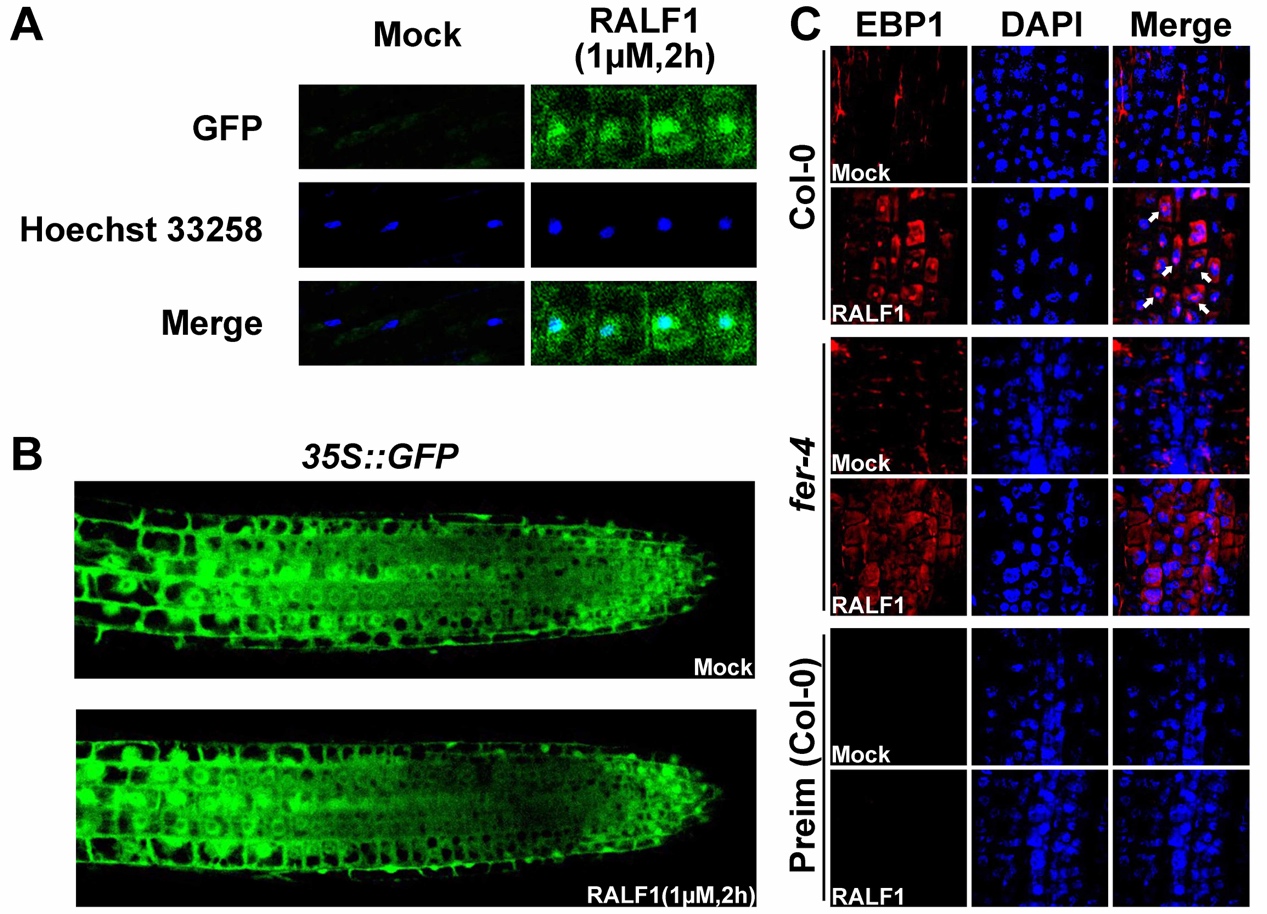
**

Supplement: S8 Fig — (A) The fluorescence of nucleus-accumulated EBP1-GFP merged with nucleus indicator signal. Nucleus was stained and indicated by Hoechst 33258 nucleus dye. The plant was treated with or without 1 μM RALF1 for 2 hours. (B) Fluorescence distribution of 35S::GFP in root with or without 1 μM RALF1 treatment for 2 hours. (C) Immune-fluorescent labeling assay. Seven-DAG seedlings (with or without 1 µM RALF1 treatment for 2 hours) were used for immune-fluorescent labeling assay. The signal of EBP1 and DAPI are shown. Preimmune serum (“preim”) was used as negative control. The nucleus fluorescence is indicated by white arrows. Assays of this figure were performed in three independent experiments, and similar results were obtained. DAG, day after germination; EBP1, ErbB3-binding protein 1; RALF1, rapid alkalinization factor 1. (DOCX) [file pbio.2006340.s008.docx]

**S9 Fig**

**
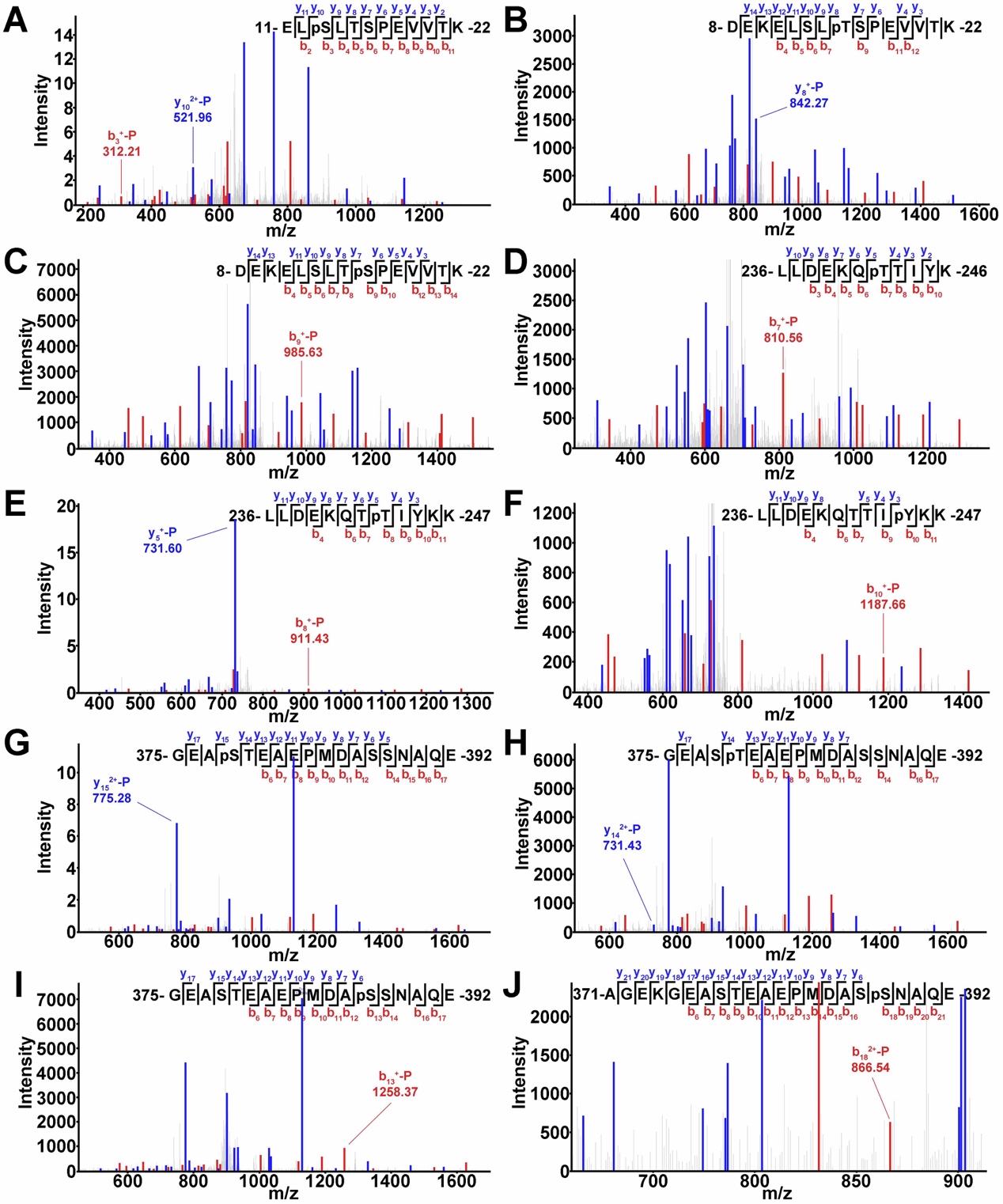
**

Supplement: S9 Fig — (A-J) The identified EBP1 phosphorylation sites of Ser13 (A), Thr15 (B), Ser16 (C), Thr242 (D), Thr243 (E), Tyr245 (F), Ser378 (G), Thr379 (H), Ser387 (I), and Ser388 (J). The identified peptide sequences and the phosphorylation site are displayed. y-ion and b-ion are shown upon the sequences. Three independent experiments were performed, and similar results were obtained. EBP1, ErbB3-binding protein 1; ESI, electrospray ionization. (DOCX) [file pbio.2006340.s009.docx]

**S10 Fig**

**
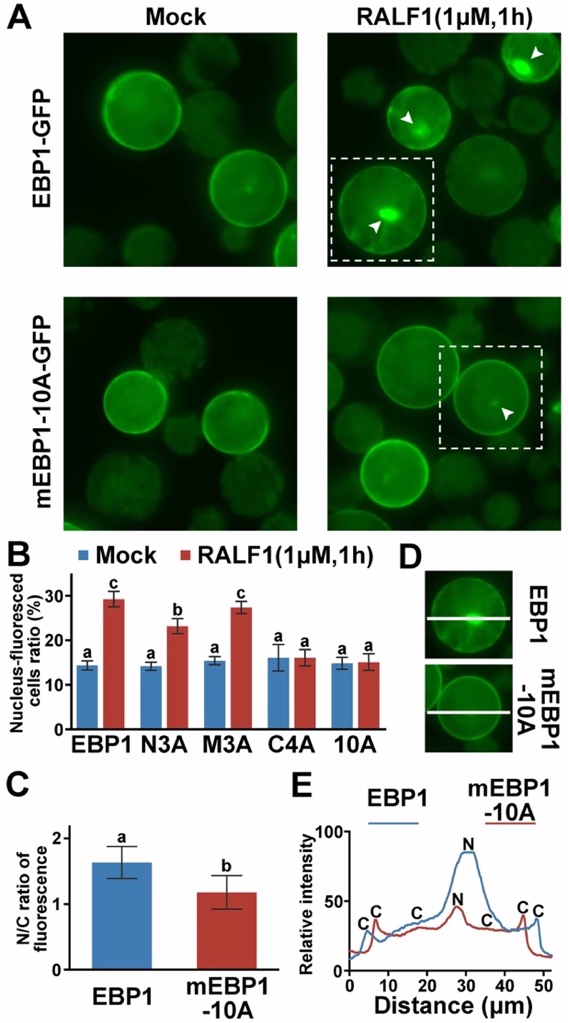
**

Supplement: S10 Fig — (A) The subcellular localization of EBP1-GFP and mEBP1-10A-GFP before and after 1 μM RALF1 treatment for 1 hour. The nucleus-localized EBP1-GFP is indicated by white arrows. (B) The ratio of nucleus-located EBP1-GFP, mEBP1-N3A-GFP (“N3A”), mEBP1-M3A-GFP (“M3A”), mEBP1-C4A-GFP (“C4A”), and mEBP1-10A-GFP (“10A”) before and after 1 μM RALF1 treatment for 1 hour. n > 330. (C) Fluorescence intensity ratio of nucleus/cytoplasm measurements in EBP1-GFP and mEBP1-10A-GFP after RALF1 treatment. n = 10. Data of (B) and (C) represent means. Data points are means +/− SD. Values with different letters are significantly different (P < 0.05) from each other, tested by one-way ANOVA. At least three independent experiments of (A–C) were performed, and similar results were obtained. (D) The representative EBP1-GFP and mEBP1-10A-GFP cells used for line scan measurement of yielded plot profiles are selected in (A) with white dashed frames. The white lines inside the images (D) show the areas used for line scan measurements that yielded plot profiles shown in (E). The chosen layer for intensity was analyzed using ImageJ. Numerical data used to generate the plot in B, C, and E are provided in S1 Data. C, cytoplasmic signal; EBP1, ErbB3-binding protein 1; GFP, green fluorescent protein; N, nucleus signal. (DOCX) [file pbio.2006340.s010.docx]

**S11 Fig**

**
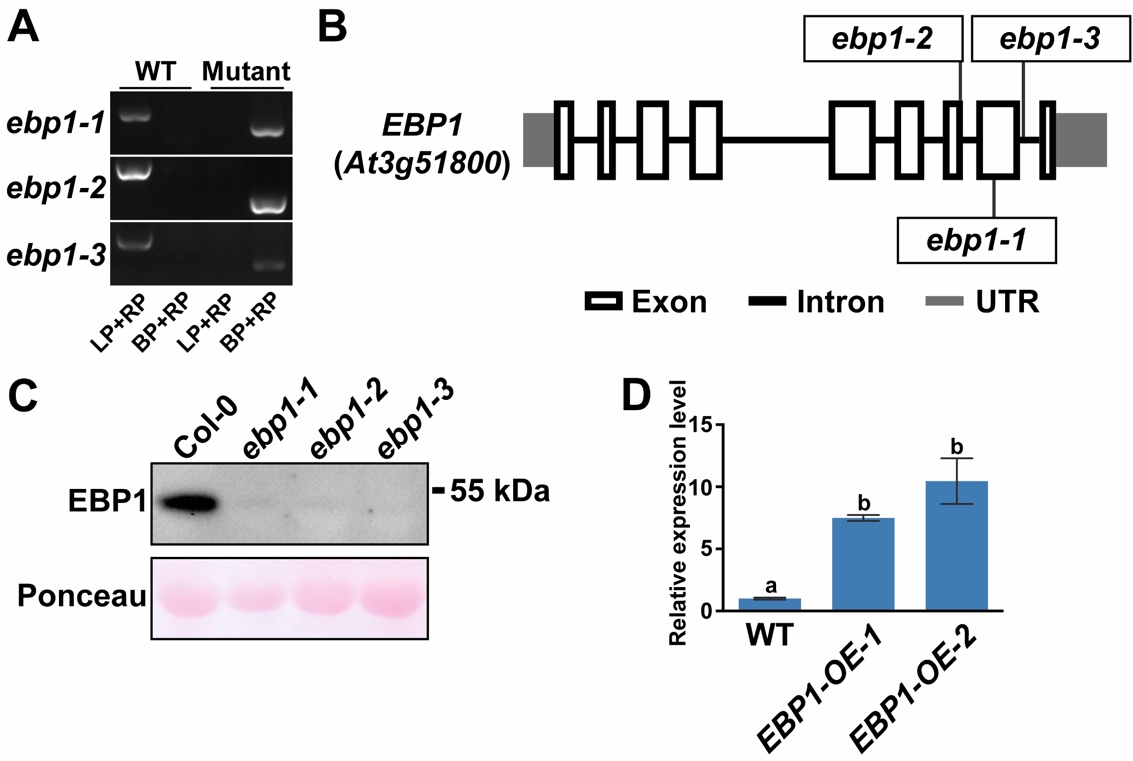
**

Supplement: S11 Fig — (A) PCR analysis of EBP1 T-DNA insertion lines. (B) The diagram of EBP1 gene structure. Exon, intron, and UTR are indicated by white frames, black lines, and gray blocks, respectively. The precise sites of the T-DNA insertions are displayed by the framed name of ebp1 mutant lines. (C) Immunoblot analyses of EBP1 protein in the WT and ebp1 mutant lines using anti-EBP1. Ponceau S staining is shown as loading control. Anti-EBP1 was used for immunoblot assay. (D) Real-time RT-PCR analysis of EBP1 mRNA levels in the WT and EBP1-OE lines. ACTIN was used as reference gene. Data represent means. Data points are means +/− SD. Values with different letters are significantly different (P < 0.05) from each other, tested by one-way ANOVA. Numerical data used to generate the plot in D are provided in S1 Data. EBP1, ErbB3-binding protein 1; EBP1-OE, EBP1-overexpression; RT-PCR, reverse transcription PCR; WT wild type. (DOCX) [file pbio.2006340.s011.docx]

**S12 Fig**

**
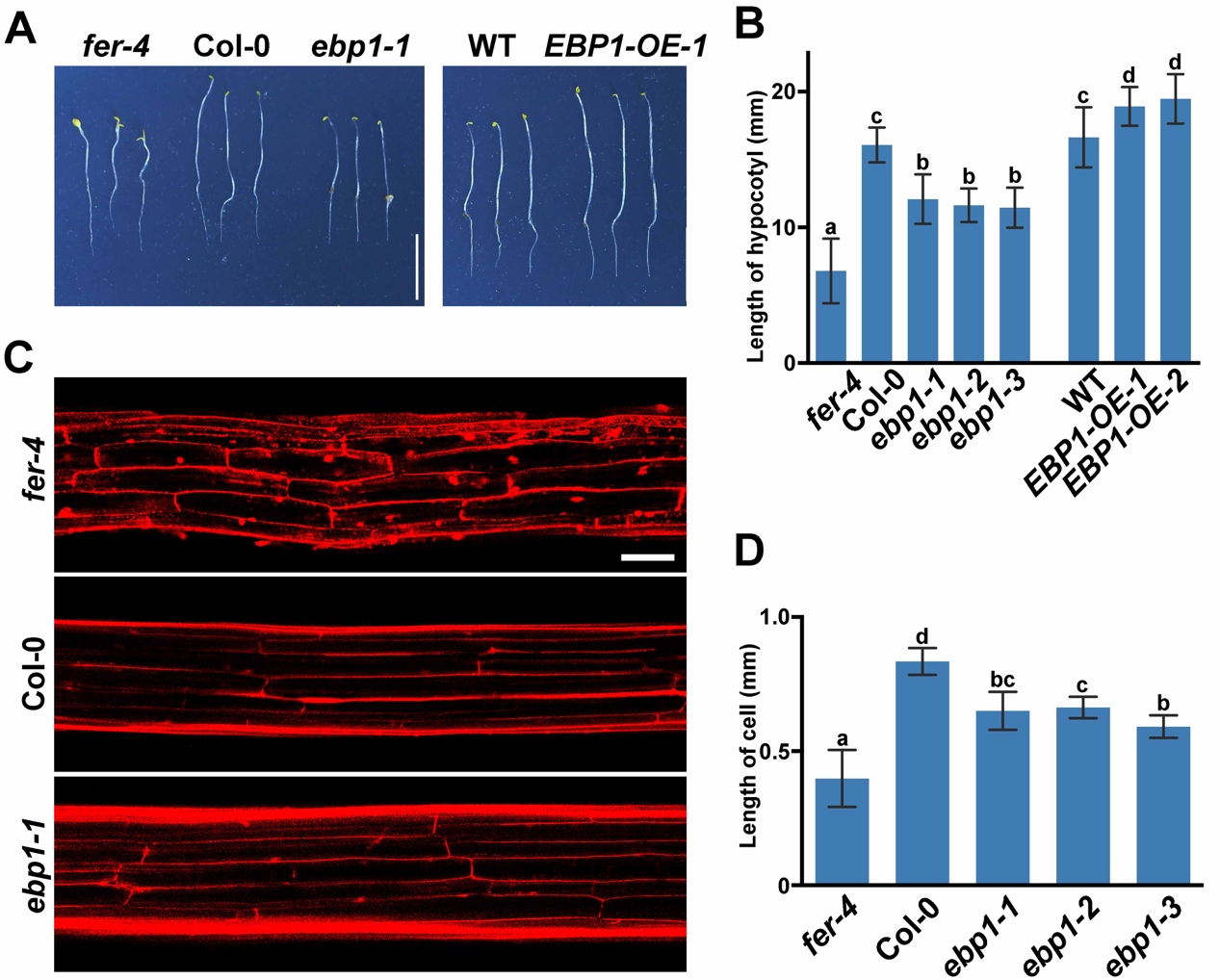
**

Supplement: S12 Fig — Phenotype (A, Bar = 10 mm) and statistical analysis (B, n > 10) show that ebp1 plants have shorter hypocotyls, whereas EBP1-OE plants show longer hypocotyls as compared to the WT. (C) Hypocotyl cell size in fer-4, Col-0, and ebp1-1. Bar = 10 μm. (D) Length of hypocotyl cells from fer-4, Col-0, and ebp1 mutants was measured using ImageJ software. n = 12. Data represent means. Data points are means +/− SD. Values with different letters are significantly different (P < 0.05) from each other, tested by one-way ANOVA. Data shown in this figure were performed in four biological replicates, and similar results were obtained. Numerical data used to generate the plot in B and D are provided in S1 Data. EBP1, ErbB3-binding protein 1; EBP1-OE, EBP1-overexpression; WT, wild type. (DOCX) [file pbio.2006340.s012.docx]

**S13 Fig**

**
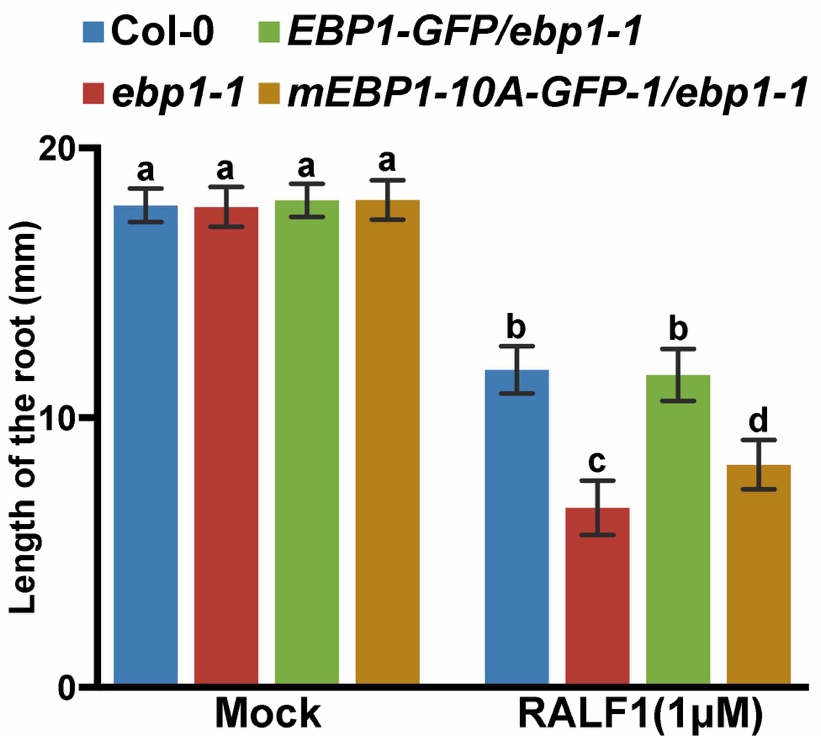
**

Supplement: S13 Fig — EBP1-GFP expressed in ebp1-1 background rescues the RALF1 sensitivity phenotype in ebp1-1 mutant. n = 25. Data points are means +/− SD. Values with different letters are significantly different from each other, tested by one-way ANOVA. Similar results were obtained in four independent experiments. Numerical data used to generate the plot are provided in S1 Data. EBP1, ErbB3-binding protein 1; RALF1, rapid alkalinization factor 1. (DOCX) [file pbio.2006340.s013.docx]

**S14 Fig**

**
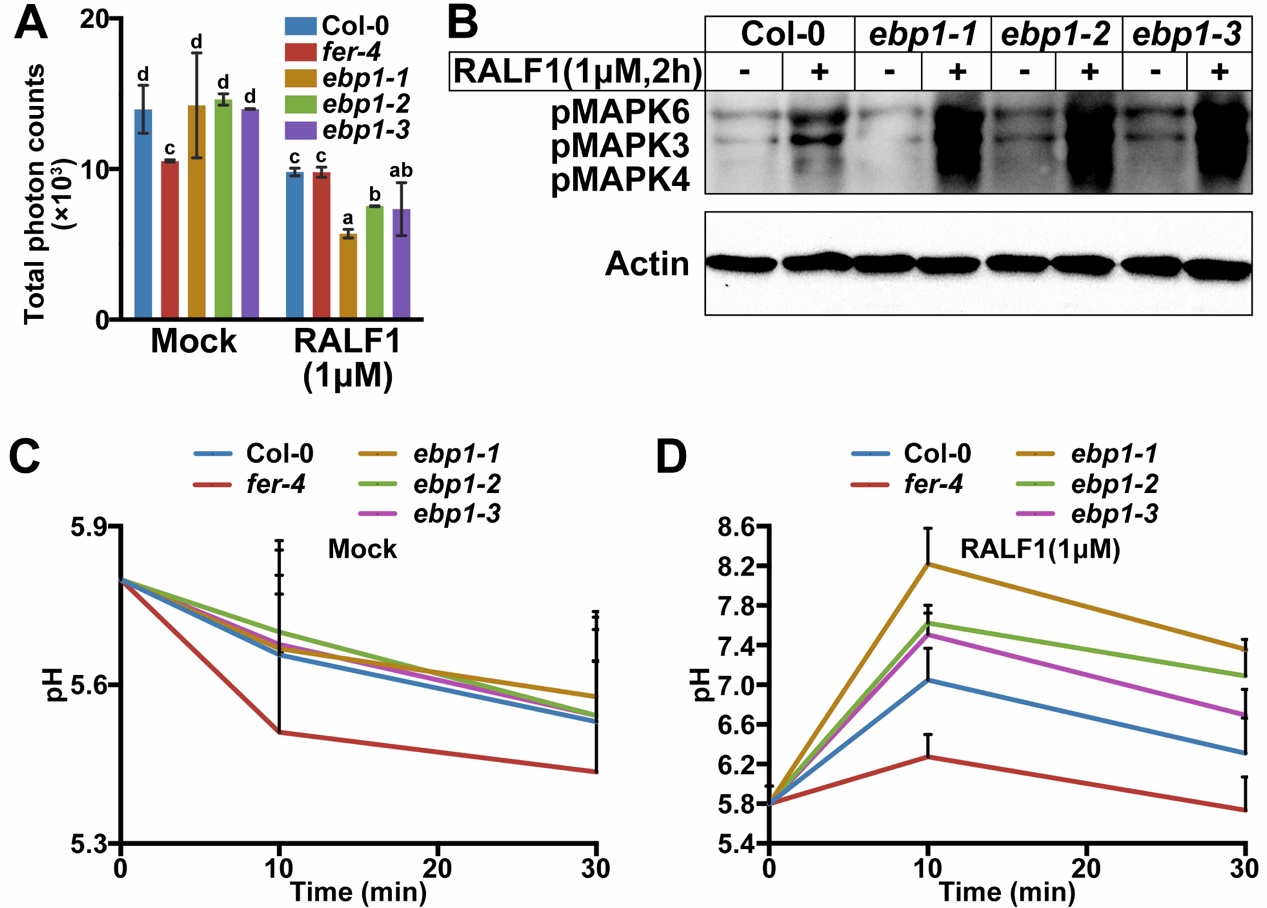
**

Supplement: S14 Fig — (A) flg22-triggered ROS burst in Col-0, fer-4, and ebp1 mutant lines without or with 1 μM RALF1 treatment. Similar results were obtained in three biological replicates. Data points are means +/− SD. Values with different letters are significantly different (P < 0.05) from each other, tested by one-way ANOVA. (B) RALF1 activated MAPK cascade immunoblot assay in Col-0, ebp1 mutant lines. Seedlings with or without 1 μM RALF1-treatment for 2 hours were used for assay. pMAPK antibody (#4370, Cell Signaling Technology) was used to detect pMAPK intensity. Actin was used as loading control. Three independent experiments were performed, and similar results were obtained. (C, D) Proton secretion assay in Col-0, fer-4, and ebp1 mutant lines with or without 1 μM RALF1. Standard curve was analyzed as y = 1.1863x − 26.314, R2 = 0.9999. Quantification of proton secretion was performed on three technical replicates. Three independent experiments were performed, and similar results were obtained. Numerical data used to generate the plot in A, C, and D are provided in S1 Data. flg22, 22 amino acid fragment of bacterial flagellin; MAPK, mitogen-activated protein kinase; pMAPK, phoshpo-MAPK; RALF1, rapid alkalinization factor 1; ROS reactive oxygen species. (DOCX) [file pbio.2006340.s014.docx]

**S15 Fig**

**
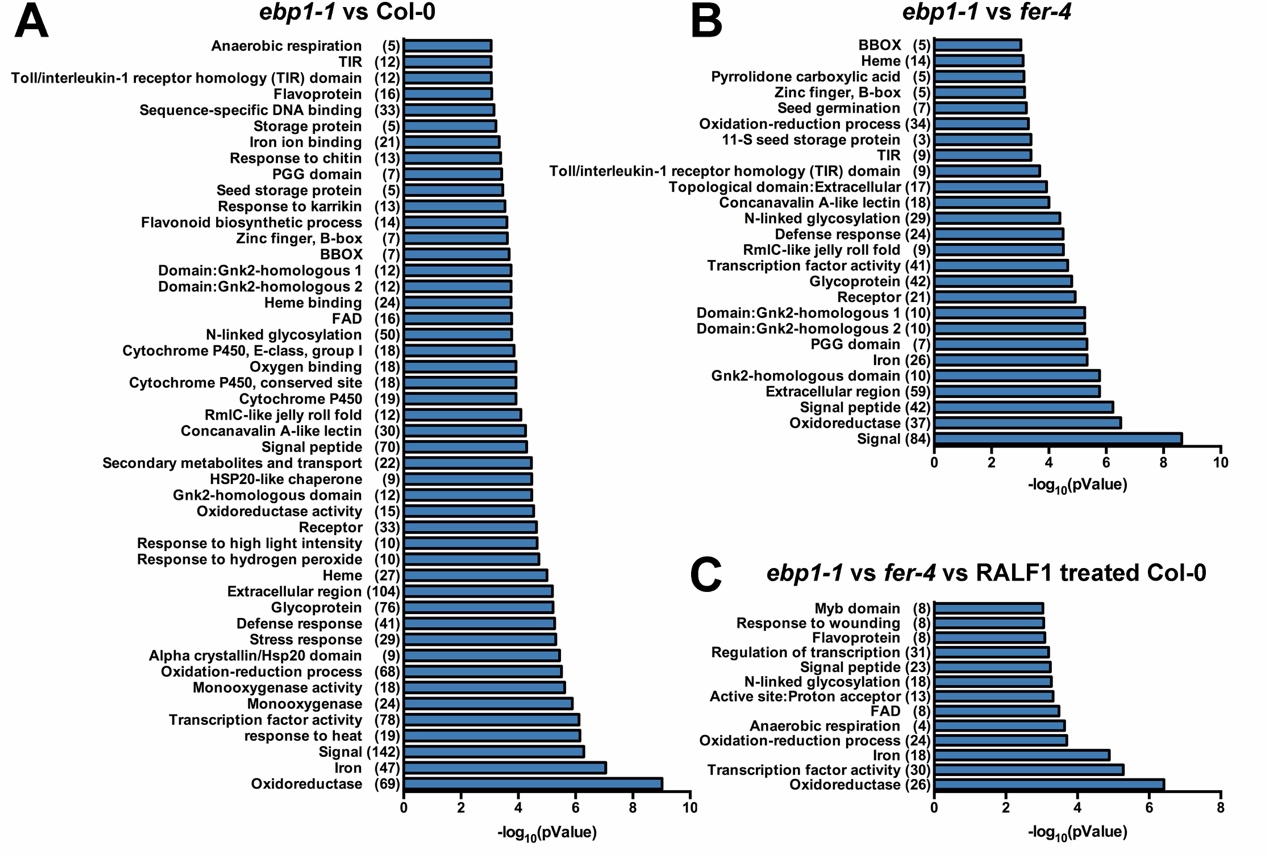
**

Supplement: S15 Fig — Functional annotation of affected genes in ebp1-1 mutant versus Col-0 (A), ebp1-1 versus fer-4 (B), ebp1-1 versus fer-4 versus RALF1-treated Col-0 (C) are shown. P value is shown as −log10 (P value). Numbers of gene in each functional annotation are shown in the brackets beside the annotations. RALF1, rapid alkalinization factor 1; RNA-seq, RNA sequencing. (DOCX) [file pbio.2006340.s015.docx]

**S16 Fig**

**
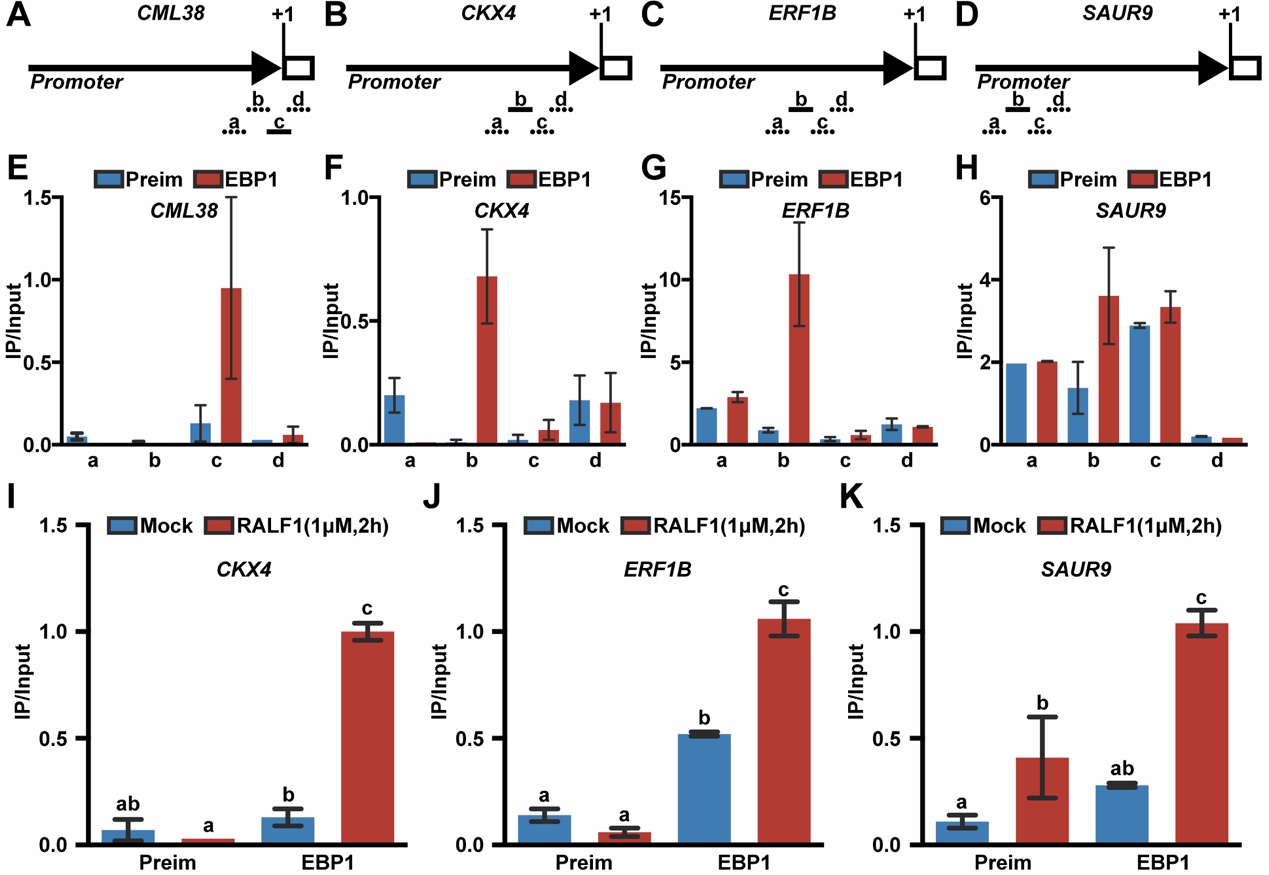
**

Supplement: S16 Fig — (A-D) The diagrams of CML38 (A), CKX4 (B), ERF1B (C), and SAUR9 (D) gene structures. The black arrows and the frame indicate the promoter and CDS sequences. Promoter fragments (a, b, c, and d) selected for ChIP-qPCR assay are shown with real line or dashed lines. The real line (or dashed line) indicates the fragment associated (or not) with EBP1 in (E-H). (E-H) ChIP-qPCR assay was performed to screen potential promoter fragments associated with EBP1. The selected promoter fragments (a, b, c, and d) of CML38 (E), CKX4 (F), ERF1B (G), and SAUR9 (H) are indicated in (A-D). Seven-DAG Col-0 seedlings (with 1 μM RALF1 treatment for 2 hours) were used for ChIP assay. (I, J, K) CKX4 (I), ERF1B (J), and SAUR9 (K) gene promoters were immune-precipitated by EBP1 protein. ChIP-qPCR results were quantified by normalization of the EBP1-IP signal with the corresponding Input signal (IP/Input). EBP1 antibody was used to immunoprecipitate EBP1 protein. Preimmune serum (“Preim”) was used for negative control. Quantification of IP/Input (E-K) levels was performed on two technical replicates. Data shown in (I–K) are representative of three independent experiments with similar results. Data represent means. Data points are means +/− SD. Values with different letters are significantly different (P < 0.05) from each other, tested by one-way ANOVA. Numerical data used to generate the plot in E-K are provided in S1 Data. CDS, coding sequence; ChIP, chromatin immunoprecipitation; DAG, day after germination; EBP1, ErbB3-binding protein 1; qPCR, quantitative PCR; RALF1, rapid alkalinization factor 1. (DOCX) [file pbio.2006340.s016.docx]

**S17 Fig**

**
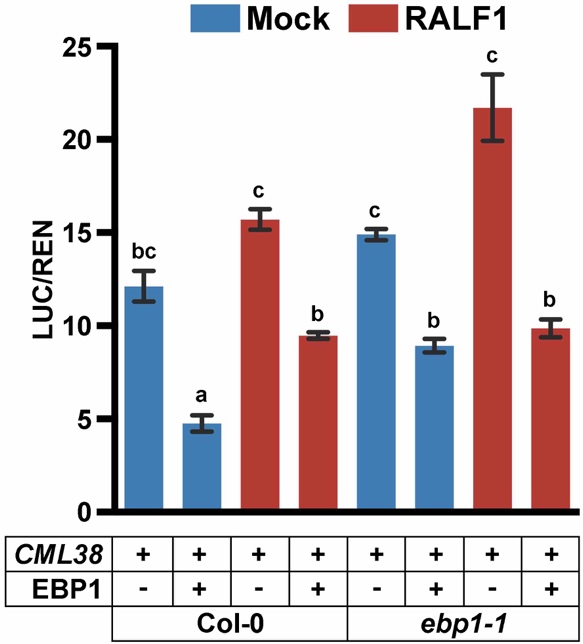
**

Supplement: S17 Fig — Relative reporter activity (LUC/REN) of indicated genotypes (Col-0 or ebp1-1), RALF1-treatment (0.1 μM RALF1) condition, and proCML38::LUC (CML38 for short) and EBP1 protein expression are shown. Quantification of LUC relative to REN levels was performed in three technical replicates. Similar results were obtained in three independent experiments. Data represent means. Data points are means +/− SD. Values with different letters are significantly different (P < 0.05) from each other, tested by one-way ANOVA. Numerical data used to generate the plot are provided in S1 Data. Dual-LUC, transient transcription dual-luciferase assay; EBP1, ErbB3, binding protein 1; LUC, luciferase; REN, Renilla luciferase; RALF1, rapid alkalinization factor 1. (DOCX) [file pbio.2006340.s017.docx]

**S18 Fig**


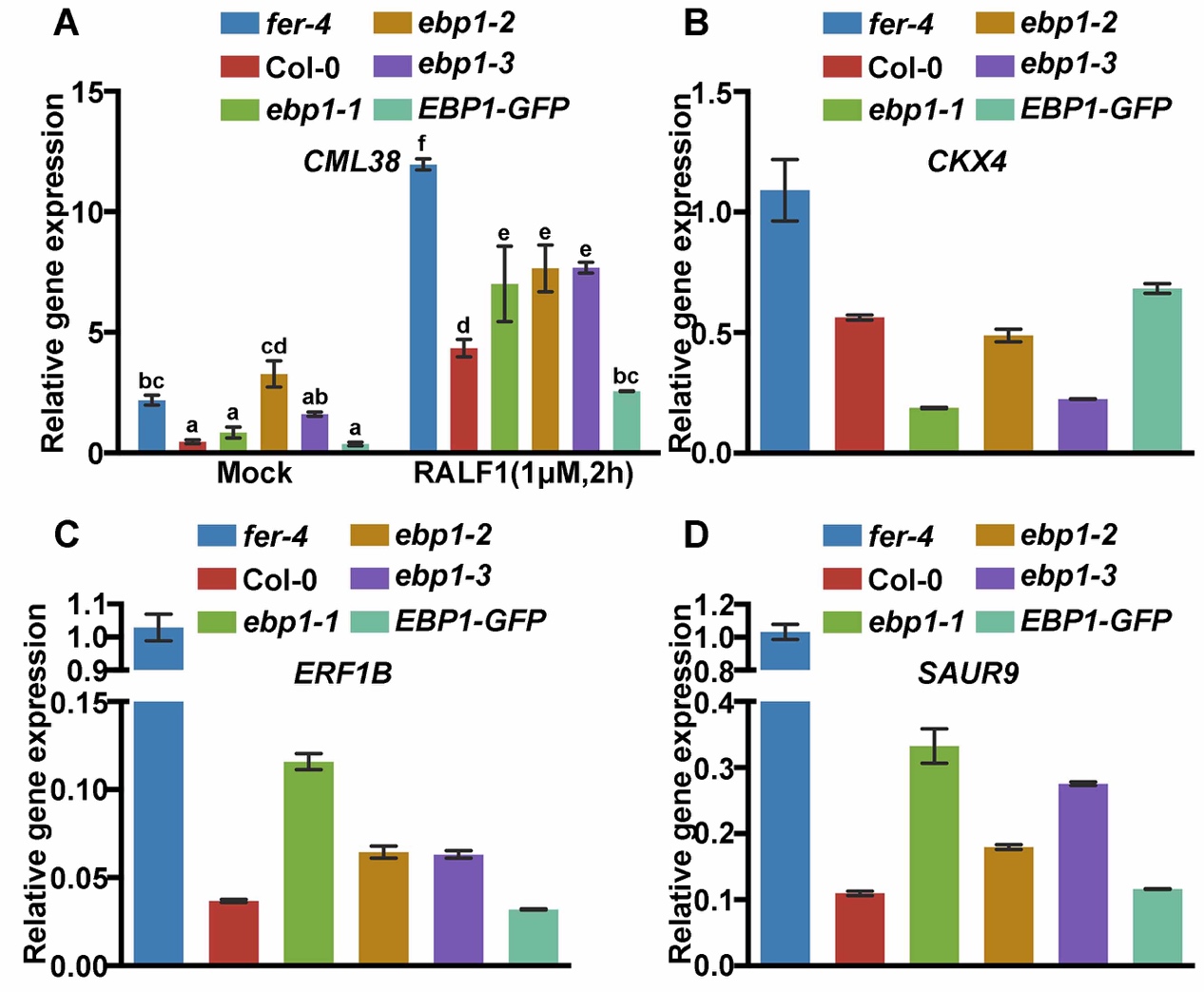

Supplement: S18 Fig — (A) Relative CML38 gene expression in ebp1 mutants and EBP1-GFP lines. RALF1 treatments (1 μM) were performed for 2 hours. Quantification of CML38 relative to Actin levels was performed. Three independent experiments were performed, and similar results were obtained. Data represent means. Data points are means +/− SD. Values with different letters are significantly different (P < 0.05) from each other, tested by one-way ANOVA. (B-D) Gene expression level of CKX4 (B), ERF1B (C), and SAUR9 (D) in Col-0, fer-4, and ebp1 mutant lines. ACTIN was used as reference gene. Similar results were obtained in two independent experiments. Numerical data used to generate the plot are provided in S1 Data. RALF1, rapid alkalinization factor 1. (DOCX) [file pbio.2006340.s018.docx]

**S19 Fig**

**
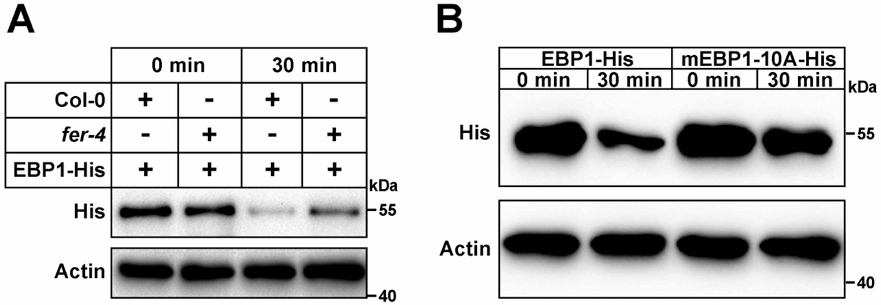
**

Supplement: S19 Fig — (A) The EBP1-His protein was incubated with Col-0 or fer-4 protein extract for 30 minutes, and (B) the EBP1-His or mEBP1-10A-His protein was incubated with Col-0 protein extract for 30 minutes, followed by SDS-PAGE and western analysis using anti-His or anti-Actin antibody. The assay was performed in three biological replicates, and similar results were obtained. EBP1, ErbB3-binding protein 1. (DOCX) [file pbio.2006340.s019.docx]
