# Supplementary material for: EBP1 nuclear accumulation negatively feeds back on FERONIA-mediated RALF1 signaling
Source: PLoS Biol. 2018 Oct 19;16(10):e2006340. doi: 10.1371/journal.pbio.2006340 (PMC6195255; doi:10.1371/journal.pbio.2006340)
Supplement: S1 Table — (DOCX) [file pbio.2006340.s021.docx]

**S1_Table**

**S1_Table. List of primers**

| Name | Primer sequence |
| --- | --- |
| EBP1-Y2H F | 5'- cgacatatgagttcggacgatgagagagacg -3' |
| EBP1-Y2H R | 5'- caggatcctcattcttgagcattactacttgcg -3' |
| EBP1-BiFC F | 5'- acagatcttgagttcggacgatgagagagacg -3' |
| EBP1-BiFC R | 5'- caggatccattcttgagcattactacttgcgtc -3' |
| EBP1-GFP F | 5'- catctagaatgagttcggacgatgagagag -3' |
| EBP1-GFP R | 5'- caggatccttcttgagcattactacttgcg -3' |
| EBP1-GST F | 5’- atggatccagttcggacgatgagagagacg -3’ |
| EBP1-GST R | 5’- atagcggccgctcattcttgagcattactacttg -3’ |
| EBP1-OE F | 5’- gctctagagccaccatgagttcggacgatgagagag -3’ |
| EBP1-OE R | 5’- actggatccattcttgagcattactacttgc -3’ |
| EBP1-FLAG F | 5’- caaaaaagcaggcttcatgagttcggacgatgagagagac -3’ |
| EBP1-FLAG R | 5’- caagaaagctgggtcttcttgagcattactacttgcgtcc -3’ |
| Ebp1 Q-PCR F | 5’- cttgtggagtgtgtgaac -3’ |
| Ebp1 Q-PCR R | 5’- cctgaagtgtatgtgaagtg -3’ |
| pEBP1::GUS pBI101.2 F | 5’- gattacgccaagcttcacagatacggacgacgtaattatt -3’ |
| pEBP1::GUS pBI101.2 R | 5’- cctacccggggatcctcgttaacgatctcagcggcgctcttg -3’ |
| EBP1 T1 LP1 | 5’- tgaaggtgttctttcccacc -3’ |
| EBP1 T1 RP1 | 5’- gatgatgaaaagtgctttgtgc -3’ |
| EBP1 T2 LP1 | 5’- gggatcaaatccttaccacg -3’ |
| EBP1 T2 RP1 | 5’- atccagccctgaaacaactg -3’ |
| pACY FER-KD-S F | 5’- gggaattccatatgcgtggtgattaccagcctgc -3’ |
| pACY FER-KD-S R | 5’- cggggtaccacgtccctttggattcatgatc -3’ |
| pACY-PYL1 F | 5’- catgccatggcgaattcagagtcctcc -3’ |
| pACY-PYL1 R | 5’- cgcggatccttacctaacctgagaagagttgt -3’ |
| pRSF-ABI1 F | 5’- gggaattccatatggaggaagtatctccggc -3’ |
| pRSF-ABI1 R | 5’- cggggtacctcagttcaagggtttgctcttgag -3’ |
| pRSF-EBP1-His F | 5’- cgcggatccgatgagttcggacgatgagagaga -3’ |
| pRSF-EBP1-His R | 5’- cccaagctttcattcttgagcattactacttgcg -3’ |
| RALF1-His-MBP F | 5’- ttcatatggcgaccacaaaatacataagct -3’ |
| RALF1-His-MBP R | 5’- cgcggatccctaactcctgcaacgagcaattt -3’ |
| CML38-a ChIP F | 5’- agcgtaaatcatacctctcaaat -3’ |
| CML38-a ChIP R | 5’- cttcacttccttcgtcttatctat -3’ |
| CML38-b ChIP F | 5’- tgagaaagaaacaagaaagagaga -3’ |
| CML38-b ChIP R | 5’- gttgtggtaatggaatgggtat -3’ |
| CML38-c ChIP F | 5’- aacactaatctaccacgtctatt -3’ |
| CML38-c ChIP R | 5’- gtcctcaccattgctactg -3’ |
| CML38-d ChIP F | 5’- actcaacctcaatcatct -3’ |
| CML38-d ChIP R | 5’- tccatcttcttctcttctt -3’ |
| CKX4-a ChIP F | 5’- atagcagatagaggagacggttta -3’ |
| CKX4-a ChIP R | 5’- gagagtgaagcacacgaatga -3’ |
| CKX4-b ChIP F | 5’- aataaatgaatcttccgactgt -3’ |
| CKX4-b ChIP R | 5’- cgaaacacatacgcaagt -3’ |
| CKX4-c ChIP F | 5’- gctttctcacgccacacta -3’ |
| CKX4-c ChIP R | 5’- cgaccaaactccgactctg -3’ |
| CKX4-d ChIP F | 5’- gcccacagagtcggagtt -3’ |
| CKX4-d ChIP R | 5’- agtcttgttgacaccaaagagaaa -3’ |
| ERF1B-a ChIP F | 5’- tgtgatgatgtgtagtgttctaat -3’ |
| ERF1B-a ChIP R | 5’- gaaaactgatgacccctctaaa -3’ |
| ERF1B-b ChIP F | 5’- ggatggttgaaagatagtttatga -3’ |
| ERF1B-b ChIP R | 5’- ccttgaatcccaaacaacaaat -3’ |
| ERF1B-c ChIP F | 5’- agccaaattatcttctaaaca -3’ |
| ERF1B-c ChIP R | 5’- aatgattaagccatattccaa -3’ |
| ERF1B-d ChIP F | 5’- tgttgagttgatccagcagttat -3’ |
| ERF1B-d ChIP R | 5’- aacctacatgccacattcagat -3’ |
| SAUR9-a ChIP F | 5’- acatcttgaacatacaataacatc -3’ |
| SAUR9-a ChIP R | 5’- ggagaagaaggacagtga -3’ |
| SAUR9-b ChIP F | 5’- tcactgtccttcttctcctt -3’ |
| SAUR9-b ChIP R | 5’- tggcttcaaccgttacatt -3’ |
| SAUR9-c ChIP F | 5’- ccctattaaatgtaacggttgaag -3’ |
| SAUR9-c ChIP R | 5’- tcatcggttgtatgtatagttgt -3’ |
| SAUR9-d ChIP F | 5’- acaactatacatacaaccgatga -3’ |
| SAUR9-d ChIP R | 5’- gcaagttcccatttcattcc -3’ |
| CML38 Q-PCR F | 5’- caatggtgaggacaagaaca -3’ |
| CML38 Q-PCR R | 5’- tacggcttcttcatcagaca -3’ |
| CKX4 Q-PCR F | 5’- aacttctggtgttactctc -3’ |
| CKX4 Q-PCR R | 5’- tgaataaccttgtcgttga -3’ |
| ERF1B Q-PCR F | 5’- ctacgaggatggttgttct -3’ |
| ERF1B Q-PCR R | 5’- cgtcttcttattggtcattctc -3’ |
| SAUR9 Q-PCR F | 5’- cgaggactggtcttcatt -3’ |
| SAUR9 Q-PCR R | 5’- cttggtaataactttgaggatttg -3’ |
| CML38 EMSA F | 5’- aacactaatctaccacgtctattacacaca -3’ |
| CML38 EMSA R | 5’- tgtgtgtaatagacgtggtagattagtgtt -3’ |
| CML38 LUC F | 5’- cgggatccatttgatcgtcatccaattaaaaagg -3’ |
| CML38 LUC R | 5’- catgccatgggagagaaaaataaatggttaagtgtatt -3’ |
| GFP R | 5’- gtgcccattaacatcaccatc -3’ |
